# A chromosome-level genome assembly for the eastern fence lizard (*Sceloporus undulatus*), a reptile model for physiological and evolutionary ecology

**DOI:** 10.1093/gigascience/giab066

**Published:** 2021-10-01

**Authors:** Aundrea K Westfall, Rory S Telemeco, Mariana B Grizante, Damien S Waits, Amanda D Clark, Dasia Y Simpson, Randy L Klabacka, Alexis P Sullivan, George H Perry, Michael W Sears, Christian L Cox, Robert M Cox, Matthew E Gifford, Henry B John-Alder, Tracy Langkilde, Michael J Angilletta, Adam D Leaché, Marc Tollis, Kenro Kusumi, Tonia S Schwartz

**Affiliations:** Department of Biological Sciences, Auburn University, Auburn, AL 36849, USA; Department of Biological Sciences, Auburn University, Auburn, AL 36849, USA; Department of Biology, California State University Fresno, Fresno, CA 93740, USA; School of Life Sciences, Arizona State University, Tempe, AZ 85287, USA; Department of Biological Sciences, Auburn University, Auburn, AL 36849, USA; Department of Biological Sciences, Auburn University, Auburn, AL 36849, USA; Department of Biological Sciences, Auburn University, Auburn, AL 36849, USA; Department of Biological Sciences, Auburn University, Auburn, AL 36849, USA; Department of Biology, Pennsylvania State University, University Park, PA 16802, USA; Department of Biology, Pennsylvania State University, University Park, PA 16802, USA; Department of Anthropology, Pennsylvania State University, University Park, PA 16802, USA; Huck Institutes of the Life Sciences, Pennsylvania State University, University Park, PA 16802, USA; Department of Biological Sciences, Clemson University, Clemson, SC 29634, USA; Department of Biology, Georgia Southern University, Statesboro, GA 30460, USA; Department of Biological Sciences, Florida International University, Miami, FL 33199, USA; Department of Biology, University of Virginia, Charlottesville, VA 22904, USA; Department of Biology, University of Central Arkansas, Conway, AR 72035, USA; Department of Ecology, Evolution, and Natural Resources, Rutgers University, New Brunswick, NJ 08901, USA; Department of Biology, Pennsylvania State University, University Park, PA 16802, USA; School of Life Sciences, Arizona State University, Tempe, AZ 85287, USA; Department of Biology, University of Washington, Seattle, WA 98195, USA; Burke Museum of Natural History and Culture, University of Washington, Seattle, WA 98195, USA; School of Life Sciences, Arizona State University, Tempe, AZ 85287, USA; School of Informatics, Computing, and Cyber Systems, Northern Arizona University, Flagstaff, AZ 86011, USA; School of Life Sciences, Arizona State University, Tempe, AZ 85287, USA; Department of Biological Sciences, Auburn University, Auburn, AL 36849, USA

**Keywords:** genome, transcriptome, squamate, reptile

## Abstract

**Background:**

High-quality genomic resources facilitate investigations into behavioral ecology, morphological and physiological adaptations, and the evolution of genomic architecture. Lizards in the genus *Sceloporus* have a long history as important ecological, evolutionary, and physiological models, making them a valuable target for the development of genomic resources.

**Findings:**

We present a high-quality chromosome-level reference genome assembly, SceUnd1.0 (using 10X Genomics Chromium, HiC, and Pacific Biosciences data), and tissue/developmental stage transcriptomes for the eastern fence lizard, *Sceloporus undulatus*. We performed synteny analysis with other snake and lizard assemblies to identify broad patterns of chromosome evolution including the fusion of micro- and macrochromosomes. We also used this new assembly to provide improved reference-based genome assemblies for 34 additional *Sceloporus* species. Finally, we used RNAseq and whole-genome resequencing data to compare 3 assemblies, each representing an increased level of cost and effort: Supernova Assembly with data from 10X Genomics Chromium, HiRise Assembly that added data from HiC, and PBJelly Assembly that added data from Pacific Biosciences sequencing. We found that the Supernova Assembly contained the full genome and was a suitable reference for RNAseq and single-nucleotide polymorphism calling, but the chromosome-level scaffolds provided by the addition of HiC data allowed synteny and whole-genome association mapping analyses. The subsequent addition of PacBio data doubled the contig N50 but provided negligible gains in scaffold length.

**Conclusions:**

These new genomic resources provide valuable tools for advanced molecular analysis of an organism that has become a model in physiology and evolutionary ecology.

## Data Description

### Context

Genomic resources, including high-quality reference genomes and transcriptomes, facilitate comparisons across populations and species to address questions ranging from broad-scale chromosome evolution to the genetic basis of key adaptations. Squamate reptiles, the group encompassing lizards and snakes, have served as important models in ecological and evolutionary physiology owing to their extensive metabolic plasticity [1]; diverse reproductive modes including obligate and facultative parthenogenesis [2]; repeated evolution of placental-like structures [2, 3]; shifts among sex-determining systems, with XY, ZW, and temperature-dependent systems represented often in closely related species [4, 5]; loss of limbs and elongated body forms [6]; and the ability to regenerate tissue [7, 8].

Despite having evolved greater phylogenetic diversity than mammals and birds, 2 major vertebrate groups with extensive genome sampling, genomic resources for squamates remain scarce and assemblies at the chromosome level are even more rare [7, 9–13]. While squamates are known to have a level of karyotypic variability similar to that of mammals [14], the absence of high-quality genome assemblies has led to their exclusion from many chromosome-level comparative genome analyses. In comparative studies, non-mammalian amniotes are often represented only by the chicken, which is divergent from squamate reptiles by almost 280 million years [15], or the green anole (*Anolis carolinensis*), whose genome is only 60% assembled into chromosomes and is lacking assembled microchromosomes [14, 16]. However, recent analyses have identified key differences that distinguish the evolution of squamate genomes from patterns found in mammals and birds [17], underscoring the need for additional high-quality genome assemblies for lizards and snakes. The development of additional squamate genomes within and across lineages will facilitate investigations of the genetic basis for many behavioral, morphological, and physiological adaptations in comparisons of organisms from the population up to higher-order taxonomic ranks.

Our goal was to develop high-quality genomic and transcriptomic resources for the spiny lizards (*Sceloporus*) to further our ability to address fundamental ecological and evolutionary questions within this taxon, across reptiles, and across vertebrates. The genus *Sceloporus* includes ∼100 species extending throughout Central America, Mexico, and the United States [18]. Researchers have used *Sceloporus* for decades as a model system in the study of physiology [19, 20], ecology [21, 22], reproductive ecology [23–25], life history [26–28], and evolution [25, 29–31]. The long history of research on *Sceloporus* species, applicability across multiple fields of biology, and the extensive diversity of the genus make this an ideal group to target for genomic resource development.

We focus on the eastern fence lizard, *Sceloporus undulatus* (NCBI:txid8520), which is distributed in forested habitats east of the Mississippi River [32]. Recently, *S. undulatus* has been the focus of studies on the development of sexual size dimorphism [33, 34], as well as experiments testing the effects of invasive species [35–37] and climate change [22, 38–40] on survival and reproduction as a model to better understand the consequences of increasing anthropogenic disturbance. The development of genomic resources for *S. undulatus*, particularly a high-quality genome assembly, will support its role as a model species for evolutionary and ecological physiology and will have immediate benefits for a broad range of comparative studies in physiology, ecology, and evolution.

To this end, we developed a high-quality chromosome-level reference genome assembly and transcriptomes from multiple tissues for *S. undulatus*. We apply this genome reference to datasets on 3 scales: (i) to address how assembly quality influences mapping of RNA sequencing (RNAseq) and low-coverage whole-genome sequencing (WGS) data, (ii) to improve upon the genomic resources for the *Sceloporus* genus by creating reference-based assemblies of draft genomes for 34 other *Sceloporus* species, and (iii) to draw broad comparisons in chromosome structure and conservation with other recently published squamate chromosome-level genomes through large-scale synteny analysis.

## Methods and Analyses

### Sequencing and assembly of the *Sceloporus undulatus* genome

Genome sequence data were generated from 2 male *S. undulatus* collected at Solon Dixon Forestry Education Center, in Andalusia, AL (31 09.49 N, 86 42.10 W). The animals were euthanized and tissues were dissected, snap-frozen in liquid nitrogen, and stored at −80°C. Procedures were approved by the Pennsylvania State University Institutional Animal Care and Use Committee (Protocol No. 44595-1).

We developed 3 *S. undulatus* genome assemblies using increasingly more data with correspondingly greater cost: (i) a SuperNova assembly containing data from 10X Genomics Chromium; (ii) a HiRise assembly containing the 10X Genomics data with the addition of Hi-C data; and (iii) a PBJelly Assembly containing the 10X Genomics data and Hi-C data, with the addition of Pacific Biosciences (PacBio) data. These assemblies are provided as supplemental files and their summary statistics are provided in Table 1.

**Table 1:** Summary statistics across genome assemblies for *Sceloporus undulatus*

Metric	Supernova Assembly (10X Chromium)	HiRise Assembly (10X Chromium + Hi-C)	PBJelly Assembly (SceUnd1.0) (10X Chromium + Hi-C + PacBio)
Coverage	46×	4,859×	4,859×
Contig N50	0.049 Mb	0.073 Mb	0.193 Mb
Scaffold N50	2.55 Mb	265.4 Mb	275.6 Mb
Scaffold N90	0.241Mb	35.4 Mb	37.1 Mb
Scaffold L50	218 Scaffolds	3 Scaffolds	3 Scaffolds
Scaffold L90	987 Scaffolds	9 Scaffolds	9 Scaffolds
Tetrapoda BUSCO (n = 3,950)			
On whole genome	89.5% Complete; 6.4% Fragmented; 4.1% Missing	90.2% Complete; 5.5% Fragmented; 4.3% Missing	90.9% Complete; 5.0% Fragmented; 4.1% Missing
On top 24 scaffolds			90.7% Complete; 4.9% Fragmented; 4.4% Missing
On predicted proteins from top 24 scaffolds			79.1% Complete; 13.7% Fragmented; 7.2% Missing
Assembly size	1.61 Gb	1.836 Gb	1.9056 Gb with gaps; 1.8586 Gb without gaps
			Annotation: 21,050 of our predicted proteins had hits in ENSEMBL

N50 (N90): The contig or scaffold length such that the sum of the lengths of all scaffolds of this size or larger is equal to 50% (90%) of the total assembly length; L50 (L90): The smallest number of scaffolds that make up 50% (90%) of the total assembly length.

In the fall of 2016, we sequenced DNA from snap-frozen brain tissue of a single juvenile male *S. undulatus* using 10X Genomics Chromium Genome Solution Library Preparation with SuperNova Assembly [41] through HudsonAlpha. The library was sequenced on 1 lane of Illumina HiSeqX (Illumina HiSeq X Ten, RRID:SCR_016385), resulting in 774 million 150-bp paired-end reads that were assembled using the SuperNova pipeline. We refer to this assembly with 46× coverage as the SuperNova Assembly.

**Figure 1:**
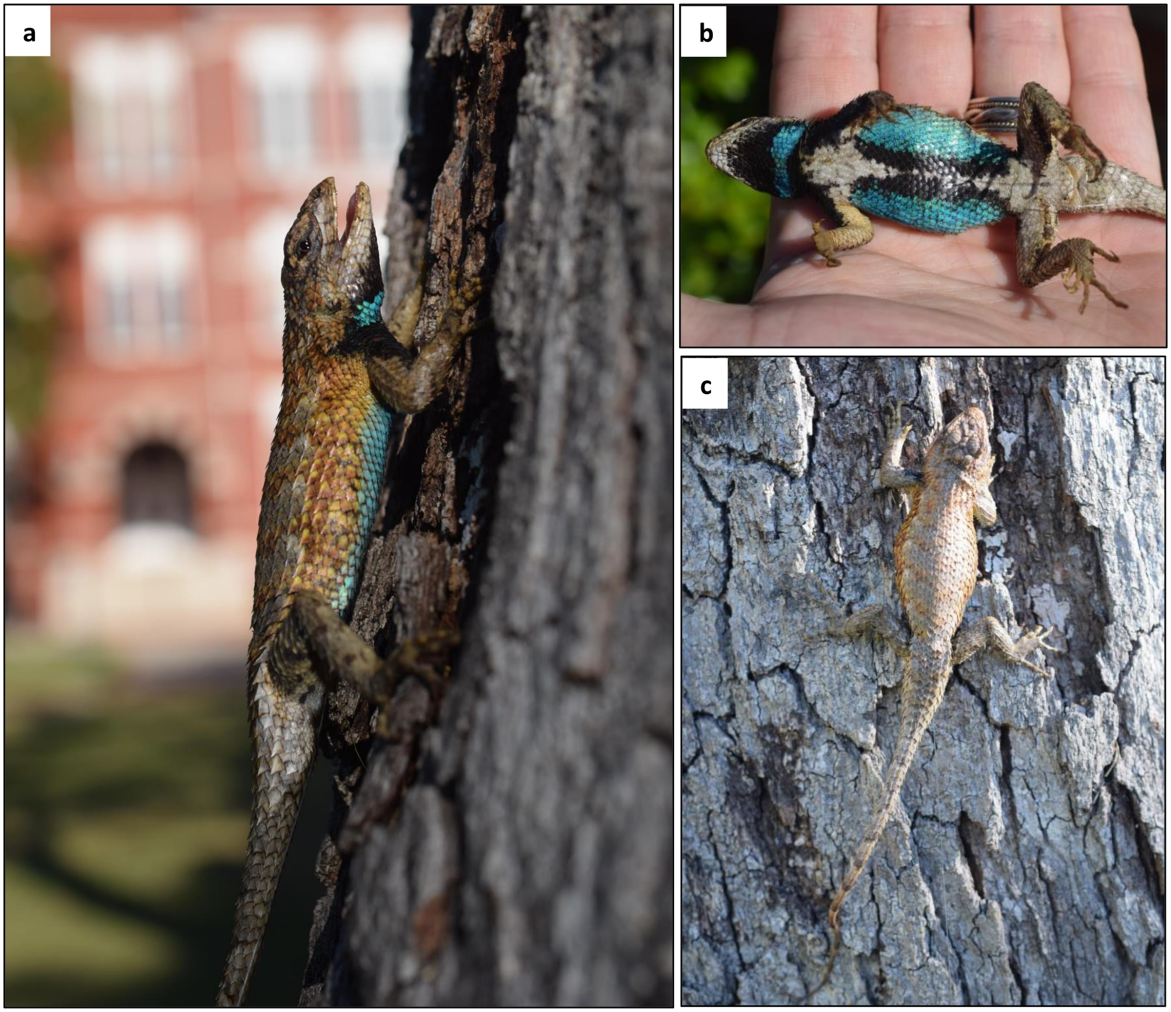
Adult male *Sceloporus undulatus* (eastern fence lizard) from Andalusia, AL, pictured outside of Samford Hall at Auburn University, (a) profile, (b) ventral, (c) dorsal view. This specimen was used for genome sequencing at DoveTail Genomics. Photo credit: R. Telemeco.

In the fall of 2017, we sequenced a second male (Fig. 1) from the same population using a Hi-C library with Illumina sequencing through Dovetail Genomics prepared from blood, liver, and muscle tissue. We used this second individual because the remains from the individual used for SuperNova Assembly were insufficient for Hi-C library preparation, which required 100 mg of tissue. Dovetail Genomics developed 2 Hi-C libraries that were sequenced on an Illumina HiSeqX to produce 293 million and 289 million (total 582 million) 150-bp paired-end reads. The data from both Hi-C and 10X Genomics were used for assembly via the HiRise software (v2.1.3-5ce4af34ac25) pipeline at DoveTail Genomics [42, 43]. This pipeline excludes contigs/scaffolds <1 kb and only uses MQ >50 reads for scaffolding, and the model fitting step uses a 10 Mb maximum. The reads were aligned with a modified SNAP pipeline. We refer to this assembly with 4,859× coverage as the HiRise Assembly.

Finally, also in fall of 2017, DNA extracted from the second adult male was used by Dovetail Genomics to generate 1,415,213 PacBio reads with a mean size of 12,418.8 bp (range, 50–82,539 bp). These PacBio data were used for gap filling to further improve the lengths of the scaffolds of the HiRise Assembly using the program PBJelly (PBJelly, RRID:SCR_012091) [44], with the following parameters: –minMatch 8 –sdpTupleSize 8 –minPctIdentity 75 –bestn 1 –nCandidates 10 –maxScore -500 –nproc 36 –noSplitSubreads. We refer to this final assembly containing all 3 types of sequencing data as the PBJelly Assembly and the final SceUnd1.0 reference genome assembly.

**Figure 2:**
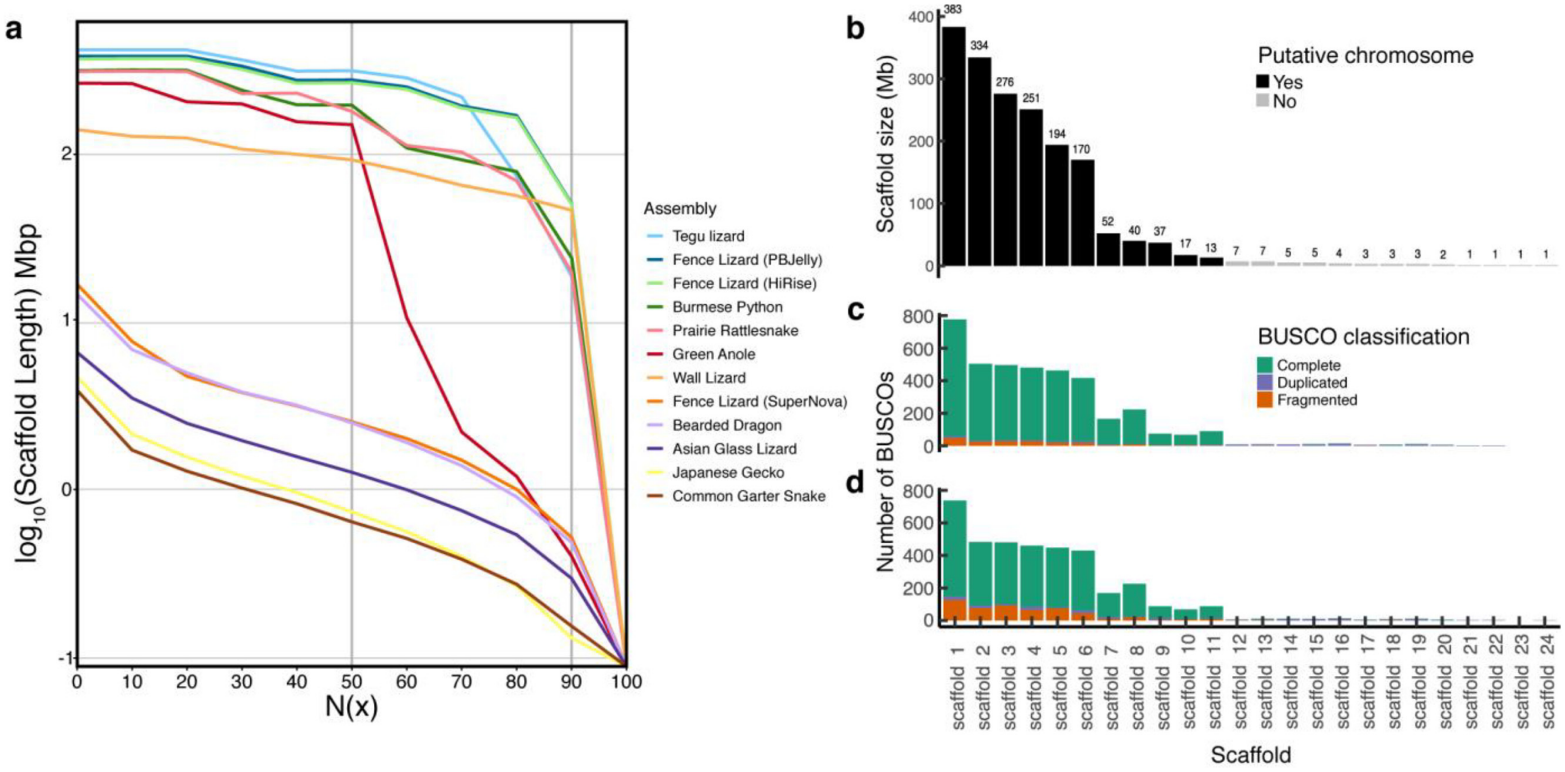
An evaluation of *Sceloporus undulatus* genome assembly quality. (a) Comparison of the contiguity of the 3 *S. undulatus* genome assemblies (fence lizard) relative to other squamate genome assemblies based on the log10 of the scaffold length. The X axis is the N(x) with the N50 and the N90 emphasized with a vertical line, representing the scaffold size that contains 50% or 90% of the data, respectively. The legend lists the assemblies in the order of the lines from most contiguous (top) to least contiguous (bottom). Note the Fence Lizard PBJelly (dark blue, SceUnd1.0) and Fence Lizard HiRise (light green) assemblies are the second and third from the top and are nearly indistinguishable. (b–d) Scaffold size distribution of SceUnd1.0 and the number of BUSCO genes that mapped to each scaffold. (b) The length of the first 24 scaffolds, where the first 11 scaffolds likely represent the haploid N = 11 chromosomes (6 macrochromosomes and 5 microchromosomes). The numbers above each bar represent scaffold length to the nearest Mb. The number of BUSCO genes that mapped to each scaffold based on (c) the genome assembly, and (d) the predicted proteins from the annotation. The 11 large scaffolds inferred to correspond to chromosomes have many unique and complete BUSCO genes (green), whereas the smaller contigs have duplicated BUSCOs (purple), suggesting that they are the result of reads not mapping correctly to the chromosomes.

For a visual comparison of our 3 *S. undulatus* assemblies and other squamate genomes, we graphed genome contiguity for these 3 assemblies with other squamate reptile genomes, building on the graph by Roscito et al. [45]. The *S. undulatus* SuperNova Assembly (containing only the 10X Genomics data) is as contiguous as the bearded dragon genome assembly (Fig. 2a). The addition of the HiRise data brought a large increase in continuity. The HiRise and PBJelly *S. undulatus* assemblies are nearly indistinguishable from each other and are among the most contiguous squamate genome assemblies to date (Fig. 2a).

The SceUnd1.0 assembly contains 45,024 scaffolds (>850 bp, without gaps) containing 1.9 Gb of sequence, with an N50 of 275 Mb. Importantly, 92.6% (1.765 Gb) of the assembled sequence is contained within the first 11 scaffolds. Chromosomal studies have determined that the *S. undulatus* karyotype is 2N = 22 with a haploid genome of N = 11 (6 macrochromosomes + 5 microchromosomes) [31, 46]. Sorting the top 11 scaffolds by size (Fig. 2b) suggests that scaffolds 1–6 are the macrochromosomes (170–383 Mb in size) and scaffolds 7–11 are the 5 microchromosomes (13–52 Mb in size) (Fig. 2b). These results suggest that the first 11 scaffolds represent the 11 chromosomes, although the assembly also produces 45,000 smaller scaffolds between 0.85 kb and 7 Mb that may still contain relevant chromosomal segments that could not be assembled. Estimated genome size of the closely related species *Sceloporus occidentalis* is 2.36 Gb on the basis of Feulgen densiometry [14]. Assuming that *S. undulatus* is similar, the 1.9 Gb of sequence in our SceUnd1.0 assembly is likely either missing some data, or repeat regions have been condensed, representing redundancies. To assess the level of contamination in our SceUnd1.0 genome assembly, we used Blobtools v1 (Blobtools, RRID:SCR_017618) [47] workflow A to estimate contamination based on GC content differences that exist between taxa. To visualize depth by GC content for taxa represented in the assembly, we created a blobDB using a BAM file to infer coverage and sequence similarity hits based on the DIAMOND blast and the SceUnd1.0 assembly fasta file. Plots were produced for 2 taxonomic ranks, phylum and order, with taxonomic annotation based on the “bestsum” taxrule. The majority of the represented taxa in the assembly were annotated as belonging to Chordata (phylum level) and Squamata (order level). There is a smaller, but visible, proportion of reads that are associated with order Testudines, which is likely due to regions of sequence similarity across reptiles. Overall, the plot demonstrates negligible contamination of other taxa (Supplementary Fig. S1).

To assess the completeness of our 3 genome assemblies, we used the BUSCO (BUSCO, RRID:SCR_015008) Tetrapoda dataset (3,950 genes) [48, 49]. For all 3 assemblies we found >89% of BUSCO genes complete (Table 1) with only minor differences in BUSCO genes between the SuperNova, HiRise, and PBJelly Assemblies (89.5%, 90.2%, 90.9% complete). This suggests that the initial SuperNova Assembly captured nearly all the genomic content despite having considerably shorter scaffolds (Table 1). The small increase in success with the more contiguous assemblies seems to result from a reduction in fragmented BUSCO genes with increasing data. In the SuperNova Assembly, 6.4% of BUSCO genes were present as fragments whereas only 5.5% and 5.0% were present as fragments in the HiRise and PBJelly Assemblies, respectively, thus explaining the 1.4% difference in complete BUSCO genes present. Interestingly, there was a 0.2% (i.e., 8 genes) increase in missing BUSCO genes from the SuperNova to the HiRise Assembly. In the PBJelly Assembly (SceUnd1.0), the BUSCO genes are almost all found on the largest 11 scaffolds (Fig. 2c), as we would predict if those scaffolds corresponded to chromosomes. Most of the BUSCO genes on the smaller scaffolds were duplicated. Even so, there are a small number of complete and fragmented BUSCO genes present on a handful of the tiny scaffolds (Fig. 2c), suggesting that these scaffolds contain pieces of the chromosomes that were not properly assembled.

### *De novo* assembly and annotation of the *Sceloporus undulatus* transcriptome

Samples used for the *de novo* transcriptome were obtained from 3 gravid female *S. undulatus* collected in Edgefield County, SC (33.7°N, 82.0°W), and transported to Arizona State University. These animals were maintained under conditions described in previous publications [50, 51], which were approved by the Institutional Animal Care and Use Committee (Protocol No. 14-1338R) at Arizona State University. Approximately 2 days after laying eggs, each lizard was killed by injecting sodium pentobarbital into the coelomic cavity. Whole-brain and skeletal muscle samples were removed and placed in RNA-lysis buffer (mirVana miRNA Isolation Kit, Ambion) and flash-frozen. Additionally, 3 early-stage embryos from each clutch were dissected, pooled together, homogenized in RNA-lysis buffer, and also flash-frozen.

Total RNA was isolated from the embryo and 3 tissue samples from each adult female (whole brain, skeletal muscle) using the mirVana miRNA Isolation Kit (Ambion) total RNA protocol. Samples were checked for quality on a 2100 Bioanalyzer (Agilent). One sample from each tissue was selected for RNAseq based on the highest RNA Integrity Number (RIN), with a minimum cut-off of 8.0. For each selected sample, 3 μg of total RNA was sent to the University of Arizona Genetics Core (Tucson, AZ) for library preparation with TruSeq v3 chemistry for a standard insert size. RNA samples were multiplexed and sequenced using an Illumina HiSeq 2000 (Illumina HiSeq 2000, RRID:SCR_020132) to generate 100-bp paired-end reads. Publicly available raw Illumina RNAseq reads from *S. undulatus* liver (juvenile male) were also added to our dataset [52, 53]. After removing adapters, raw reads from the 4 tissues were evaluated using FastQC [54] and trimmed using Trimmomatic v-0.32 [55], filtering for quality score (≥Q20) and using HEADCROP:9 to minimize nucleotide bias. This procedure yielded 179,374,469 quality-filtered reads. Table 2 summarizes read-pair counts from whole brain, skeletal muscle, whole embryos, and liver.

**Table 2:** *Sceloporus undulatus de novo* transcriptome assembly statistics

Assembly	1 Tissue [51]	3 Tissues	4 Tissues
Total of Trinity transcripts	158,323	492,249	547,370
Total of Trinity “genes"	138,031	422,687	467,658
GC%	43.81	42.85	42.76
Contig N50	1,720	1,648	1,438
Contig E90N50	2,254	2,640	2,550
Mean contig length (bp)	833.0	822.4	781.5
Transcripts with the longest ORFs	86,630 (54.7%)	212,172 (43.1%)	217,756 (39.8%)

The 4 tissues comprise 3 tissues first reported in this study (brain, skeletal, and embryos) from gravid females collected in Edgefield County, SC, plus liver tissue previously reported by McGaugh et al. 2015 [51].

All trimmed reads were pooled and assembled *de novo* using Trinity v-2.2.0 with default *k*-mer size of 25 [56]. From the final transcriptome, a subset of contigs containing the longest open reading frames (ORFs), representing 123,323 transcripts, was extracted from the *de novo* transcriptome assembly using TransDecoder v-3.0.0 (TransDecoder, RRID:SCR_017647) [57] with homology searches against the databases UniProtKB/SwissProt [58] and PFAM [59]. The transcriptome was annotated using Trinotate v-3.0 (Trinotate, RRID:SCR_018930) [60], which involved searching against multiple databases (as UniProtKB/SwissProt, PFAM, signalP, GO) to identify sequence homology and protein domains, as well as to predict signaling peptides. This pooled Tissue-Embryo Transcriptome and annotation are provided as supplemental files.

The most comprehensive transcriptome, obtained using reads from 4 tissues, consists of 547,370 contigs with a mean length of 781.5 nucleotides (Table 2)—shorter than other assemblies because of the range of contig sizes that varied among datasets (1, 3, and 4 tissues; Supplementary Table S1, Fig. S2). The N50 of the most highly expressed transcripts that represent 90% of the total normalized expression data (E90N50) was lowest in the assembly based on 1 tissue (Table 2). To validate the *de novo* transcriptome data, trimmed reads from the 4 tissues used for RNA sequencing (brain, skeletal muscle, liver, and whole embryos) were aligned back to the Trinity-assembled contigs using Bowtie2 v2.2.6 (Bowtie2, RRID:SCR_016368) [61]. From the 176,086,787 reads that aligned, 97% represented proper pairs (Supplementary Table S2), indicating good read representation in the *de novo* transcriptome assembly. To assess quality and completeness of the assemblies, we first compared the *de novo* assembled transcripts with the BUSCO Tetrapoda dataset, with BLAST+ v2.2.31 [62] and HMMER v3.1b2 (HMMER, RRID:SCR_005305) [63] as dependencies. This procedure revealed that the *de novo* transcriptome assembly captured 97.1% of the expected orthologues (sum of completed and fragmented), a result comparable to the 97.8% obtained for the green anole transcriptome using 14 tissues [64] (Table 3). Next, nucleotide sequences of *de novo* assembled transcripts with the longest ORFs were compared to the protein set of *A. carolinensis* (AnoCar2.0, Ensembl) using BLASTX (BLASTX, RRID:SCR_001653) (e-value = 1e−20, max_target_seqs = 1). This comparison showed that 11,223 transcripts of *S. undulatus* have nearly full-length (>80%) alignment coverage with *A. carolinensis* proteins (Supplementary Table S3). Predicted proteins of *S. undulatus* were also used to identify 13,422 one-to-one orthologs with proteins of *A. carolinensis* through reciprocal BLAST (e-value = 1e−6, max_target_seqs = 1). Table 4 summarizes the *de novo* transcriptome annotation results.

**Table 3:** BUSCO results for transcriptomes of 2 lizard species

Parameter	*Sceloporus undulatus*			*Anolis carolinensis*
	1 tissue	3 tissues	4 tissues	14 tissues
Complete genes (%)	72.5	91.7	92.3	96.7
Duplicated genes (%)	25	43.8	43.9	37.9
Fragmented genes (%)	9.2	4.8	4.8	1.1
Missing genes (%)	18.3	3.5	2.9	2.2
Reference	McGaugh et al. 2015 [51]	This study	This study	Eckalbar et al. 2013 [59]

For *Sceloporus undulatus*, the 4 tissues are the 3 tissues (brain, skeletal muscle, and embryos) first reported here with the addition of 1 tissue (liver) from McGaugh et al. 2015 [51]. For *Anolis carolinensis*, see Eckalbar et al. 2013 [59] for the complete list of tissues used.

**Table 4.** Annotation of *Sceloporus undulatus de novo* transcriptome assembly using 4 tissues

Annotation	Value
Annotated genes	467,658
Annotated transcript isoforms	547,370
Annotated isoforms/genes	1.17
Transcripts with Swiss-Prot annotation	(71,944)
Transcripts with PFAM annotation	51,018 (46,432)
Transcripts with KEGG annotation	65,694 (21,520)
Transcripts with GO annotation	73,936 (66,554)

Parentheses indicate unique annotation numbers.

### Genome assembly annotation

Using the 24 largest scaffolds of the SceUnd1.0 assembly (we refer to this set as SceUnd1.0_top24), we used the Funannotate v1.5.0 pipeline [65] for gene prediction and functional annotation. Funannotate uses RNAseq data and the Tetrapoda BUSCO [48] dataset to train the *ab initio* gene prediction programs Augustus [66] and GeneMark-ET [67]. Evidence Modeler is used to generate the consensus from Augustus and GeneMark-ES/ET. In the training step, we used 4 raw RNAseq datasets described in Table 5 that contained a total of 68 sequenced libraries. tRNAscan-SE (tRNAscan-SE, RRID:SCR_010835) [68] was used to predict transfer RNA (tRNA) genes. Finally the genes were functionally annotated via InterProScan (InterProScan, RRID:SCR_005829) [69], eggNOG (eggNOG, RRID:SCR_002456) [70], Pfam (Pfam, RRID:SCR_004726) [59], UniProtKB [58], MEROPS (MEROPS, RRID:SCR_007777) [71], CAZyme, and GO ontology. We also used DIAMOND blastp [72] to compare the predicted proteins to ENSEMBL human, chicken, mouse, and green anole lizard databases (Data archived files: SceUnd1.0_top24.gff3; SceUnd1.0_top24_CompiledAnnotation.csv). Our annotation pipeline predicted 54,149 genes, 15,472 of which were attributed meaningful functional annotation beyond “hypothetical protein.” Through BLAST of the predicted protein-coding genes, we found 21,050 (39%) had hits in ENSEMBL. We then quantified the number of BUSCO genes identified in the predicted proteins from the Funannotate pipeline and found 79.1%, which corresponds to an 11.6% decrease from the number of complete BUSCO genes in the SceUnd1.0 genome assembly. Because there were more BUSCOs fragmented or missing from the predicted proteins (the annotation) than the actual genomic sequence itself, we attribute those to annotation errors, not errors in the assembly, which suggests that this first version of annotation can be improved. SceUnd1.1 (a slightly updated version of SceUnd1.0 based on NCBI requirements) was submitted to NCBI for annotation. The SceUnd1.1 is version JAGXEY010000000, GenBank accession GCA_019175285.1. This Whole Genome Shotgun project has been deposited at DDBJ/ENA/GenBank under the accession JAGXEY000000000.

**Table 5.** RNAseq datasets used for training the genome annotation pipeline

Dataset	Data type	NCBI SRA Accession No.	Tissue	Age	Sex	Treatment/Condition
1. This article	100 bp PE	SAMN06312743	Skeletal muscle	Adult	Female	Post-reproductive
		SAMN06312741	Brain	Adult	Female	Post-reproductive
		SAMN06312742	Whole embryo	Embryo	N/A	
2. McGaugh et al. 2015 [53]	100 bp PE	SRR629640	Liver	Juvenile	Male	Control lab
3. Cox et al. submitted for publication	125 bp PE	SAMN14774299–321	Liver	Juvenile	Female	Blank
					Male	Castrated
					Male	Control
					Female	Testosterone
					Male	Testosterone
4. Simpson et al. in preparation	150 bp PE	SAMN08687228–45	Liver	Adult	Male	Control lab
						Acute heat stress
						Fire ant bitten

Datasets 1 and 2 were also used in the *de novo* transcriptome assembly. Data are accessible through NCBI BioProjects: 1. PRJNA371829; 3. PRJNA629371; 4. PRJNA437943.

We used annotation and sequence homology to identify the X chromosome. Sex chromosomes are highly variable among *Sceloporus* species, and the genus seems to have evolved multiple variations of XY systems [31]. However, some species, including *S. undulatus*, do not seem to have morphologically distinct sex chromosomes [73]. While the ancestral condition is heteromorphic chromosomes with a minute Y, many species within the genus demonstrate multiple sex chromosome heteromorphisms (i.e., multiple forms of the X chromosome) or have evolved indistinct sex chromosomes, such as the *undulatus* species group [18]. These heteromorphisms are likely the result of other chromosomes’ fusions to the X, as *Sceloporus* are among the large portion of iguanian lizards with conserved sex chromosomes, and another *Sceloporus* species within the same broad 2n = 22 radiation, *Sceloporus malachiticus*, has an X chromosome homologous to the green anole X but fused to several microchromosomes [74]. Given this observed homology, we used known X chromosome genes from the green anole to identify the scaffold likely representing the X chromosome within *S. undulatus*. We blasted 16 X-linked genes from the green anole downloaded from Ensembl (AnoCar2.0: ACAD10, ADORA2A, ATP2A2, CCDC92, CIT, CLIP1, CUX2, DGCR8, FICD, MLEC, MLXIP, ORAI1, PLBD2, PUS1, TMEM119, ZCCHC8) [75, 76] to SceUnd1.0. They almost exclusively map to the tenth largest scaffold (Fig. 2b), indicating that it is likely the X chromosome. The Y chromosome could not be independently identified from the assembly, most likely owing to the homomorphic nature of *S. undulatus* sex chromosomes; higher sequence homology may have caused the Y chromosome to assemble with the X chromosome [31]. This result, that the fourth predicted microchromosome is the putative X chromosome, is further supported by a separate synteny analysis described below.

### Repeat annotation and evolutionary analysis

To estimate the repetitive landscape of the *S. undulatus* genome, we modeled repeats *de novo* by running RepeatModeler v1.0.8 (RepeatModeler, RRID:SCR_015027) [77] on the SceUnd1.0 assembly. We then annotated repeats in the assembly using RepeatMasker v4.0.7 (RepeatMasker, RRID:SCR_012954) [78] with the *de novo* consensus repeat library. To estimate evolutionary divergence within repeat families in the *S. undulatus* genome, we generated repeat-family–specific alignments and calculated the average Kimura-2-parameter divergence from consensus within each family, correcting for high mutation rates at CpG sites with the calcDivergenceFromAlign.pl RepeatMasker tool. We compared the divergence profiles of *S. undulatus* and *A. carolinensis* by completing parallel analyses. We annotated repeats in the *A. carolinensis* genome (AnoCar2.0) with RepeatMasker and the “anolis” repeat library from RepBase release 20170127 [79].

**Figure 3:**
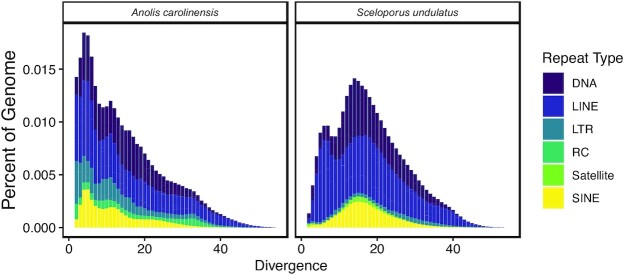
Age distributions of the major repetitive elements found in the *Anolis carolinensis* (AnoCar2.0) and *Sceloporus undulatus* (SceUnd1.0) genome assemblies. The repeat landscapes depict the relative abundance of repeat types in the genome vs their Kimura divergence from their consensus. DNA: DNA transposons; LINE: long interspersed nuclear element; LTR: long terminal repeat retrotransposon; RC: rolling circle Helitron; SINE: short interspersed nuclear element.

The *S. undulatus* assembly contained a diverse repertoire of repeats including transposable elements, the most abundant of which are the long interspersed nuclear elements (LINEs, Supplementary Table S4) comprising ∼15% of the genome. Relative proportions of LINEs, short interspersed nuclear repeats (SINEs), long terminal repeat (LTR) retrotransposons, and DNA transposons were similar to those of *A. carolinensis*. The diversity of repeat elements in *S. undulatus* mirrors that of the *Anolis* genome [80], as well as that of other squamates [17]. However, the age distribution of elements between the 2 genomes was vastly different (Fig. 3). For instance, a much larger proportion of the *Anolis* genome was comprised of transposable element insertions ≤10% from their family consensus. This indicates an overabundance of inserts resulting from recent activity in *A. carolinensis* relative to *Sceloporus*. In particular, the *Anolis* genome contained far more recent SINEs (Kruskal-Wallis test; *P* = 9.374e−05). The distribution of recent LINEs was significantly different between the 2 genomes (*P* = 2.824e−06), and *Anolis* contained more recent insertions from the L1 family (*P* = 0.0001571), as well as RTE-BovB (*P* = 0.001152) and R4 (*P* = 0.0001571). The *Anolis* genome also contained more recent LTR retrotransposons (*P* = 1.153−e07), as well as Mariner (*P* = 0.0002122), Tigger (*P* = 0.01017), and Chapaev (*P* = 0.001152) DNA transposons.

### Mitochondrial genome assembly

The mitochondrial genome was not captured by the genome sequencing approaches, likely owing to how these types of libraries are prepared. However, mitochondrial sequence data obtained via RNAseq can be effectively assembled into whole mitochondrial DNA (mtDNA) genomes [81–84]. We used RNAseq reads from 18 *S. undulatus* individuals from the RNAseq Dataset 4 (Table 5), which are from the same population as the individuals used for the genome sequencing. We used Trimmomatic v0.37 (Trimmomatic, RRID:SCR_011848) [55] to clean the raw reads and then mapped the clean reads to a complete *S. occidentalis* mtDNA genome [85] using BWA v0.7.15 (BWA, RRID:SCR_010910) [86]. Of the 632,987,330 total cleaned reads, 9.73% mapped to the *S. occidentalis* mtDNA genome with an average read depth of 5,164.42 reads per site per individual. After sorting and indexing mapped reads with SAMTOOLS v1.6 (SAMTOOLS, RRID:SCR_002105) [87], we used the mpileup function in SAMTOOLS to build a consensus mitochondrial genome (mtGenome) excluding the reference and filling the no-coverage regions with “N” to generate 100% coverage of the mtGenome based on the consensus across the 18 individuals. We mapped the consensus genome to the well-annotated *A. carolinensis* mtGenome with MAFFT v1.3.7 (MAFFT, RRID:SCR_011811) [88] and transferred the annotation using the “copy annotation” command in GENEIOUS v.11.1.5 (GENEIOUS, RRID:SCR_010519) [89]. Annotations from the *A. carolinensis* mtGenome (17,223 bp) transferred well to the newly assembled *S. undulatus* mtGenome (17,072 bp), with 13 protein-coding genes, 22 tRNA regions, 2 ribosomal RNA regions, and a control region (see full list in Supplemental Results File). While this genome is useful for understanding sequence variation and comparative genomics and phylogenetic analyses, this mitochondrial genome should not be used for examination of mitochondrial genome structure. The mitochondrial genome and the annotation are provided as supplemental data.

### Addressing reference assembly quality using population-level transcriptomic and genomic data

In developing the high-quality reference genome for *S. undulatus*, we produced 3 assemblies using increasing amounts of data, for correspondingly greater costs. To assess the utility of each of the assemblies for addressing ecological genomic questions, we use 2 datasets: RNAseq and whole-genome resequencing.

First, we used RNAseq Dataset 4 (Table 5) from n = 18 males that were sampled from the same population (Alabama) as the individuals that were used to develop the reference assemblies; we then used these data to test whether the percentage of reads that mapped to the reference varied depending on which assembly we used as the reference. RNAseq data were cleaned with Trimmomatic v0.37 [55] and mapped with HISAT2 v2.1.0 [90] to each of the 3 *S. undulatus* genome assemblies. The percentages of reads that mapped were calculated using SAMTOOLS v1.6 flagstat [87]. We found negligible differences in mapping the RNAseq data to the SuperNova, HiRise, and PBJelly assemblies, where 81.49%, 82.37%, and 82.28% of cleaned reads mapped, respectively (Table 6).

**Table 6.** Comparison of each genome assembly type as a reference for population-level analyses for RNAseq and WGS of *Sceloporus undulatus* individuals from Alabama (AL, either low or high coverage), Tennessee (TN), and Arkansas (AR)

Assembly	Parameter	RNAseq, AL	Low coverage, AL	High coverage, AL	High coverage, TN	High coverage, AR
SuperNova	QC-passed reads	3.28E7 ± 6.83E6	5.11E7 ± 3.36E7	3.33E8 ± 2.66E7	3.47E8 ± 9.39E7	3.33E8 ± 6.14E7
	Reads mapped					
	No.	2.68E7 ± 6.19E6	5.07E7 ± 3.34E7	3.30E8 ± 2.65E7	3.43E8 ± 9.13E7	3.23E8 ± 6.69E7
	%	81.49 ± 0.09	99.29 ± 0.11	99.29 ± 0.08	98.80 ± 0.60	96.84 ± 4.75
	Whole-genome (×)	NA	3.56 ± 2.95	23.02 ± 10.52	23.33 ± 11.25	22.27 ± 10.81
	HET SNP sensitivity	NA	0.58	0.93	0.91	0.91
HiRise	QC-passed reads	3.30E7 ± 6.86E6	5.11E7 ± 3.36E7	3.33E8 ± 2.66E7	3.47E8 ± 9.39E7	3.33E8 ± 6.14E7
	Reads mapped					
	No.	2.71E7 ± 6.30E6	5.07E7 ± 3.34E7	3.30E8 ± 2.65E7	3.43E8 ± 9.13E7	3.23E8 ± 6.69E7
	%	82.37 ± 0.09	99.29 ± 0.11	99.29 ± 0.08	98.80 ± 0.60	96.84 ± 4.75
	Whole genome (×)	NA	3.56 ± 2.95	23.02 ± 10.52	23.33 ± 11.25	22.27 ± 10.81
	HET SNP sensitivity	NA	0.58	0.93	0.91	0.91
PBJelly	QC-passed reads	3.29E7 ± 6.84E6	5.09E7 ± 3.35E7	3.31E8 ± 2.64E7	3.45E8 ± 9.29E7	3.31E8 ± 6.09E7
	Reads mapped					
	No.	2.71E7 ± 6.25E6	5.06E7 ± 3.33E7	3.29E8 ± 2.63E7	3.41E8 ± 9.05E7	3.22E8 ± 6.66E7
	%	82.28 ± 0.09	99.46 ± 0.11	99.47 ± 0.08	98.97 ± 0.61	97.00 ± 4.78
	Whole-genome (×)	NA	3.36 ± 2.97	21.75 ± 11.46	22.04 ± 12.14	21.04 ± 11.64
	HET SNP sensitivity	NA	0.55	0.88	0.87	0.86

Datasets were mapped to either the SuperNova Assembly containing only the 10X Genomics Chromium data, the HiRise Assembly containing 10X Genomics Chromium and Hi-C data, or the PBJelly assembly (SceUnd1.0) containing 10X Genomics Chromium, Hi-C, and PacBio data. Mean SAMTOOLS QC-passed reads, reads mapped, and percentage of mapped QC-passed reads for every sequencing depth and population are shown along with mean whole-genome coverage and theoretical HET SNP sensitivity for every assembly and population. Data are available in NCBI BioProject: PRJNA656311.

Second, we prepared genomic DNA libraries for massively parallel sequencing for n = 10 *S. undulatus* individuals (6 females, 4 males) from the same Alabama population as the individuals that were used to develop the reference assemblies. We also prepared libraries for n = 5 *S. undulatus* individuals (1 female, 4 males) from Edgar Evins, TN, and for n = 5 individuals (2 females, 3 males) from St. Francis, AR. This Arkansas population is at the borders of the *S. undulatus* and *Sceloporus consobrinus* geographic distributions, making its taxonomic status uncertain [18]. Specifically, we followed standard protocols for tissue DNA extraction from toe and/or tail clips with OMEGA EZNA Tissue spin-column kits. We then prepared sequencing libraries using the Illumina TruSeq Nano kit. We multiplexed these libraries with other individuals not included in this analysis and sequenced the library pool across 2 Illumina NovaSeq 6000 S4 sequencing runs. Five individuals from each of the 3 populations were sequenced to ∼20× average read coverage; the remaining 5 individuals from Alabama were sequenced to lower coverage (∼3×). Raw sequence read data were trimmed with Trimmomatic [55] and mapped separately to each of the 3 *S. undulatus* assemblies with bwa_mem [86]. SAMTOOLS flagstat [87] was used to calculate the total number of alignments in the .sam files generated during mapping and the number of shotgun reads that mapped to each assembly. The CollectWgsMetrics tool from the Picard Toolkit [91] was used to calculate genome-wide coverage of the mapped reads for each individual and assembly, and theoretical HET SNP sensitivity (a metric based on coverage and base-quality distribution that estimates the probability of calling a true heterozygote SNP) as a way to predict the utility of each assembly as a reference for calling SNPs at high and low coverage. For all sequencing depths and populations, we observed fewer total alignments to the PBJelly Assembly than to either the HiRise or Supernova Assemblies (Table 6). Even though there were <0.5% fewer total reads that passed quality control (QC) with the PBJelly Assembly/ SceUnd1.0, a higher percentage of the QC-passed reads mapped to this assembly than to either the HiRise or Supernova Assemblies (Table 6). We also determined that individuals from the same population as the *S. undulatus* individuals used to create these reference assemblies had a higher percentage of reads map to the assemblies than individuals from the Tennessee or Arkansas populations (Table 6). Those reads had lower whole-genome coverage and lower theoretical HET SNP sensitivity when mapped to the PBJelly/SceUnd1.0 Assembly than either the HiRise or Supernova Assemblies (Table 6). This may be due to repetitive regions being added to the assembly by the PacBio data, making it slightly less mappable. Both the RNAseq and the whole-genome resequencing datasets support the conclusion that the 10X Chromium data that were used for the SuperNova Assembly covered the genome sufficiently to be a good reference for mapping RNAseq and WGS data and that the HiC data (included in the HiRise Assembly) and the PacBio data (included in the final PBJelly Assembly) did not increase the amount of sequence information. Rather, the use of the HiC data and PacBio data resulted in larger scaffolds, which will aid in understanding the genomic context of expression data and sequence variants.

### Assembly and refinement of genomic data for 34 additional *Sceloporus* species

**Figure 4:**
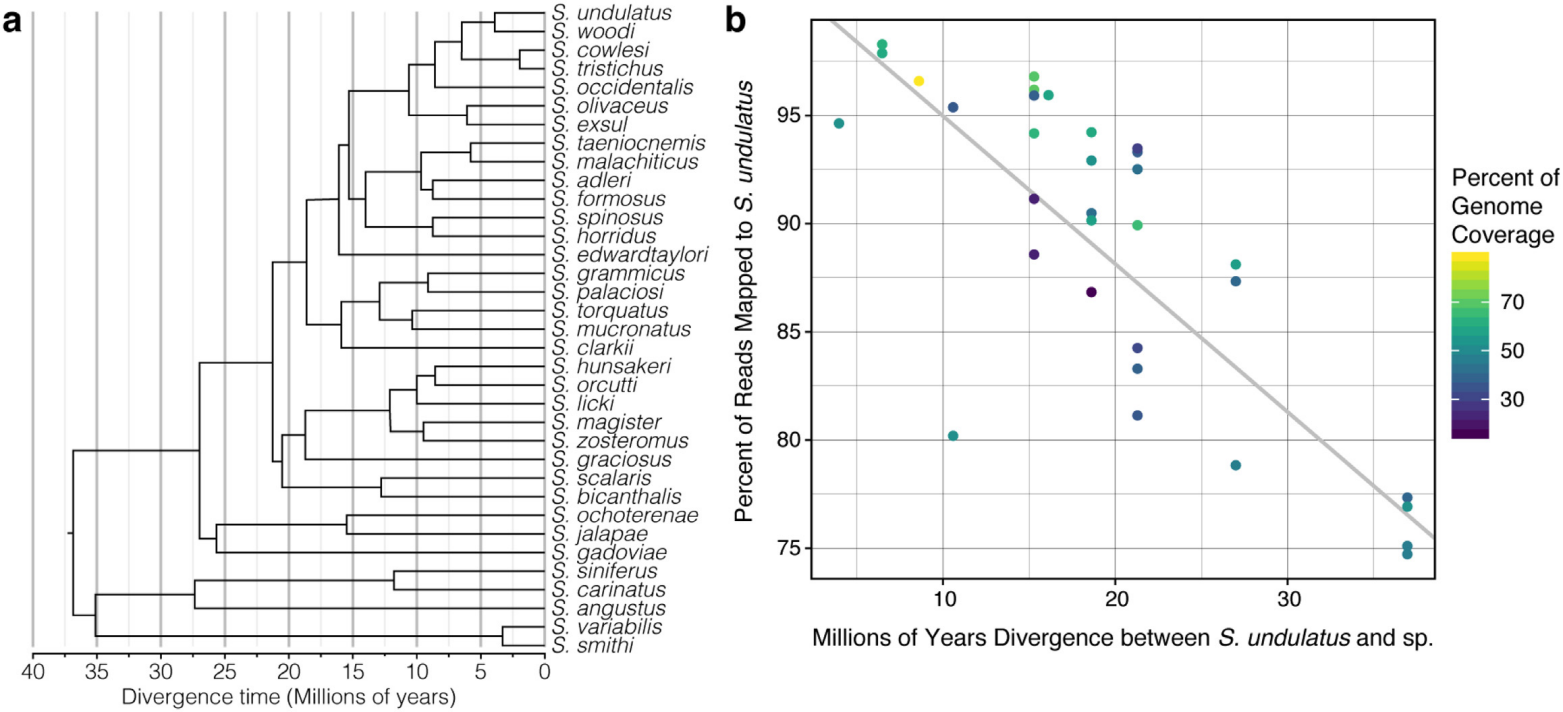
Relationship between divergence time and effectiveness of using the *Sceloporus undulatus* assembly for reference-based mapping. (a) Phylogenetic relationships and divergence times of selected *Sceloporus* species, according to Leaché et al. [99]. For the purpose of illustration only the species used in our analysis are shown. (b) Relationship between percent reads mapped to the *S. undulatus* reference genome (SceUnd1.0) and time of divergence from *S. undulatus* with a linear regression. The color of the dots represents the percent of the genome that is covered, which was affected by the number of redundant sequences in the reduced representation library for a particular species.

Draft reduced-representation genomes are available for 34 species within *Sceloporus* [92, 93] (phylogeny in Fig. 4a). We downloaded the raw genomic reads for these 34 *Sceloporus* species from the SRA (Study Accession SRP041983; Table 7). Genomic resources for 33 of the species were obtained using reduced-representation libraries (yielding ∼5 Gb per species), while 1 species, *S. occidentalis*, was sequenced using whole-genome shotgun sequencing (40.88 Gb; Table 7) [92]. To improve the draft assemblies for these 34 species, we mapped these raw reads to the final assembly, SceUnd1.0, using BWA-MEM [94]. Only the 11 longest, putative chromosome scaffolds from the SceUnd1.0 were used. The GATK version 3 [95–97] RealignerTargetCreator and IndelRealigner tools were used for local realignment, and HaplotypeCaller was used to identify insertion/deletion (INDEL) and single nucleotide polymorphism (SNP) variants. These sequence variants were separated and filtered with the SelectVariants and VariantFiltration tools using the GATK base settings. BEDTools [98] “genomecov" tool was used to calculate coverage and identify regions with no coverage. We generated consensus sequences for each species by writing variants back over the reference fasta and replacing nucleotides with no coverage with “N”, using BCFtools [87] “consensus" for SNPs and BEDTools “maskfasta" for indels and regions with no mapping coverage (Supplemental Code File).

**Table 7.** *Sceloporus* species with partial genomic sequence assemblies updated using SceUnd1.0 as a reference

Species	SRA Accession	Gigabases	Original *de novo* assembly, %			Reference-based assembly, %			
			Coverage	BUSCO Comp	BUSCO Frag	Mapped	Coverage	BUSCO Comp	BUSCO Frag
*S. occidentalis*	SRX545583	40.88	61.01	16.2	32.8	96.59	88.68	90.2	5.7
*S. adleri*	SRX542351	6.14	0.88	0	0	94.18	63.2	25.8	23.3
*S. angustus*	SRX542352	5.9	1.18	0.1	1.1	74.73	46.43	33.0	27.7
*S. bicanthalis*	SRX542353	5.1	1.74	0.2	1.6	92.52	42.26	7.0	19.5
*S. carinatus*	SRX542354	7.96	1.38	0.2	1.2	75.11	46.47	31.7	31.1
*S. clarkii*	SRX542380	3.92	0.08	0.0	0.0	86.84	15.71	0.8	3.0
*S. cowlesi*	SRX542355	4.93	3.78	0.2	3.1	97.88	60.17	13.7	21.6
*S. edwardtaylori*	SRX542356	4.57	1.37	0.1	1.4	95.94	58.21	13.8	20.8
*S. exsul*	SRX542357	3.57	0.04	1.7	0.3	80.2	52.16	6.0	16.3
*S. formosus*	SRX542358	6.5	1.81	0.1	1.7	96.19	70.49	39.1	27.1
*S. gadoviae*	SRX542359	5.82	1.06	0.2	0.9	87.34	40.13	4.4	14.8
*S. graciosus*	SRX542383	4.53	NA	0.1	0.4	84.72	7.13	0.1	0.4
*S. grammicus*	SRX542360	4.76	1.81	0.1	1.7	92.92	52.8	12.2	20.7
*S. horridus*	SRX542361	3.74	0.17	0.2	0.9	95.92	37.49	1.6	7.0
*S. hunsakeri*	SRX542362	4.42	1.14	1.8	0.9	83.3	38.41	2.8	10.6
*S. jalapae*	SRX542363	6.96	1.5	0.0	0.0	88.12	56.49	34.4	31.0
*S. licki*	SRX542364	3.38	0.95	1.4	1.0	93.31	36.81	2.1	9.1
*S. magister*	SRX542365	3.5	0.8	1.7	0.7	84.26	31.74	1.2	5.6
*S. malachiticus*	SRX542384	4.55	0.11	0.1	0.4	91.15	22.27	0.9	4.2
*S. mucronatus*	SRX542366	5.54	1.25	0.2	1.4	94.23	60.02	20.9	25.3
*S. ochoterenae*	SRX542367	6.63	1.57	0.3	2.5	78.84	46.78	17.6	21.6
*S. olivaceus*	SRX542368	3.14	1.11	1.2	0.9	95.38	35.89	1.4	8.2
*S. orcutti*	SRX542369	3.88	0.99	1.8	0.9	81.14	35.79	1.9	8.8
*S. palaciosi*	SRX542370	6.59	1.58	0.1	1.5	90.49	42.11	3.4	11.3
*S. scalaris*	SRX542371	6.56	1.04	0.2	1.8	89.93	65.53	47.0	24.9
*S. smithi*	SRX542373	4.75	1.18	0.1	0.8	77.35	39.47	7.7	16.8
*S. spinosus*	SRX542374	5.91	1.51	0.1	1.1	96.8	69.15	36.0	26.9
*S. taeniocnemis*	SRX542382	3.68	0.14	0.1	0.4	88.58	22.35	0.9	3.7
*S. torquatus*	SRX542375	6.78	1.75	0.3	2.2	90.15	57.36	20.1	21.4
*S. tristichus*	SRX542376	5.36	4.67	0.3	3.4	98.29	62.09	17.4	22.8
*S. utiformis*	SRX542381	4.13	0.06	0.0	0.3	63.97	17.42	1.1	3.7
*S. variabilis*	SRX542377	7.59	1.5	0.2	1.2	76.93	52.22	38.8	30.2
*S. woodi*	SRX542378	3.52	0.7	1.7	0.8	94.64	52.36	6.4	17.9
*S. zosteromus*	SRX542379	2.71	0.62	1.3	0.9	93.48	29.39	0.7	5.3
Mean (excluding *S. occidentalis*)			1.23				44.4		

Genomic resources for 34 of the species were obtained using reduced representation libraries [93], while 1 species, *S. occidentalis*, was sequenced using whole-genome shotgun sequencing [92]. The data were downloaded from the SRA (Study Accession SRP041983 [93]). Gigabases refer to the amount of sequence data for each library.

Mapping the reduced representation genome data from the 33 additional *Sceloporus* species improved the assemblies for each species. It seems there was a considerable amount of by-catch in many of the reduced-representation sequences that is normally filtered out when those reduced representation data are analyzed. For the species with ∼5 Gb of sequencing data, we improved the genome coverage from a mean of 1.23% to a mean of 44.4% coverage at low depth (1–3×) (Supplementary Fig. S3). For *S. occidentalis* with ∼41 Gb of data, coverage improved from 61.0% to 88.7% (Table 7), at an average ∼20× depth (Supplementary Fig. S3). Across the 33 species with ∼5 Gb of data, the BUSCO genes identified (complete and fragmented) in the reference-based assemblies ranged from 0.5% to 71.9% (complete and fragmented), whereas *S. occidentalis* had 95.9% BUSCO genes (complete and fragmented) identified, similar to our *S. undulatus* SuperNova Assembly (Table 7). Notably, across the *Sceloporus* genus, the percentage of the raw data that mapped to the reference was negatively correlated with divergence time to the reference, *S. undulatus* (*P* < 0.0001, *r* = 0.779; Fig. 4b). For species that are less than ∼20 million years diverged from *S. undulatus*, >90% of reads mapped; the percentage of reads mapped declined to 75% when divergence was >35 million years (Fig. 4b).

It is important to note that the reference-based assemblies produced for these 34 species will correspond 1:1 with the synteny of the *S. undulatus* scaffolds. However, *Sceloporus* is notable among squamates for remarkable chromosome rearrangements with karyotypes ranging from 2N = 22 to 2N = 46 [31]. Therefore, the genome assemblies for species with karyotypes other than 2N = 22 (the *S. undulatus* reference) or with large chromosomal inversions will not be reliable for addressing questions related to genomic architecture or structural variation [100]. However, these draft genomes contain a substantial amount of data that can be used for comparative genomic analyses. Supplementary Fig. S4 demonstrates the overlap in coverage of SceUnd1.0 by the reference-based genome assemblies. These distributions estimate that 50% of the genome would be covered by a subset of 16 species. Focusing on 1 gene of interest to our group, *IGF1*, we found that 16 of the 34 species had >75% coverage across the protein-coding region of this gene and 24 of them had >50% coverage (Supplementary Fig. S4). Thereby, this dataset should prove useful for analyses of protein and gene sequence evolution to understand behavioral ecology, physiology, developmental biology, and more.

### Analysis of synteny with other squamate chromosome-level genomes

**Figure 5:**
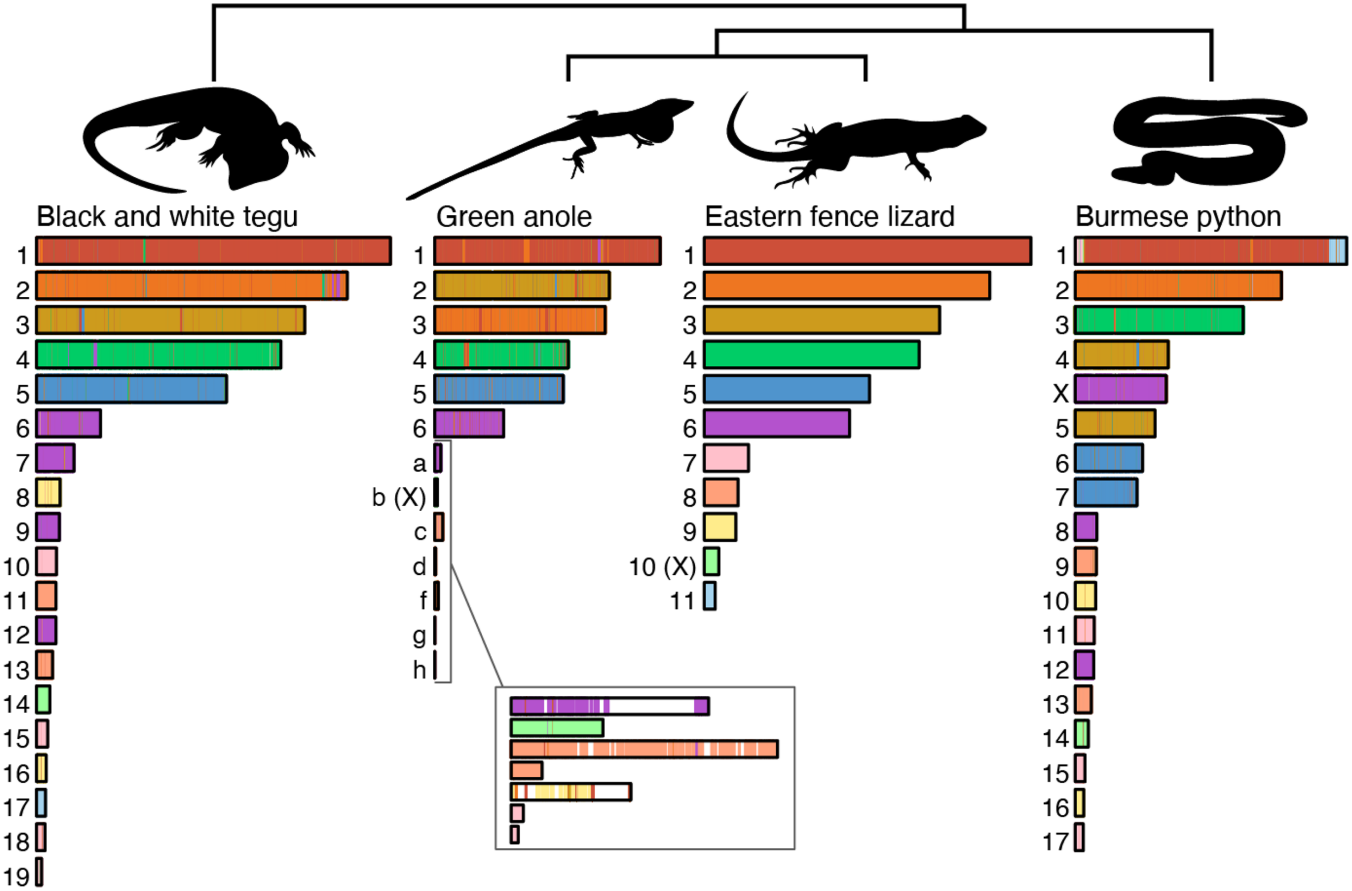
Marker-based synteny painting of fence lizard (*Sceloporus undulatus*) scaffolds/chromosomes onto the tegu (*Salvator merianae*), green anole (*Anolis carolinensis*), and python (*Python bivittatus*) assemblies. The color indicates synteny for that scaffold. The linkage groups representing microchromosomes in the green anole are lettered and expanded to visualize the colors. The white areas did not have a high-confidence match between the anole and the fence lizard to paint. Putative sex chromosomes are indicated with uppercase letters.

As another benchmark of genome completeness, and to generate an initial look at chromosome evolution among squamates, we performed synteny analysis of the eastern fence lizard (*S. undulatus*) SceUnd1.0 assembly with the green anole (*A. carolinensis*, AnoCar2.0) and with recently published chromosome-level assemblies for the Burmese python (*Python bivittatus*) [101] and the Argentine black and white tegu lizard (*Salvator merianae*) [45] (available at NCBI BioProject: PRJNA473319). The SceUnd1.0 scaffolds representing the 11 putative chromosomes were each divided into 1,000-bp-long sequences that excluded gapped regions to serve as markers. Using BLAST, these markers were compared to the predicted chromosomes from the python and tegu HiC assemblies. BLAST hits for each were filtered to only include unique hits that had >80% identity and were ≥500 bp long and part of 4 consecutive hits from the same eastern fence lizard chromosome, a method previously used for synteny analysis for the prairie rattlesnake [102]. Using these results, the eastern fence lizard chromosomes were painted onto the anole, python, and tegu chromosomes to visualize large-scale synteny (Fig. 5).

The decreased chromosome number in the *S. undulatus* species group compared to other *Sceloporus* lineages and the Iguanian group has long driven a hypothesis that a high number of fusions occurred in chromosomes in this species group, which is evident in the marker-based synteny painting of the *S. undulatus* genome. While the incomplete nature of the green anole genome, especially the lack of microchromosomes, makes many *Sceloporus* lineage-specific fusions difficult to identify, the inclusion of the tegu and python genomes provides guidance. For example, tegu chromosomes 6, 7, 9, and 12 are all syntenic to fence lizard chromosome 6. However, the tegu chromosomes 6 and 7 occur in a single block as the python X chromosome, and we cannot discern whether this was a fusion in a lineage preceding Iguanians and snakes or a fission in the tegu. The tegu chromosomes 9 and 12 are syntenic to python chromosomes 8 and 12, which may have been fused in the fence lizard, considering the considerable size difference between fence lizard chromosome 6 and the syntenic green anole chromosome 6. Similarly, tegu chromosomes 8 and 16 are syntenic to python chromosomes 10 and 16, fusing to form the fence lizard chromosome 9 but almost completely absent from the green anole assembly. These synteny results further support that the tenth largest chromosome in the SceUnd1.0 assembly is syntenic to the anole X chromosome (Fig 5). However, it is not syntenic to the python X chromosome, which is syntenic to the Z chromosome in other snakes. The tegu sex chromosome has not been identified. On the basis of the blast hits from the anole X-linked genes and this synteny analysis we define the tenth largest chromosome in the SceUnd1.0 assembly as the putative X chromosome, but functional data are needed to confirm this assignment.

## Discussion

For the advancement of reptilian genomic and transcriptomic resources, we provide a high-quality, chromosome-level genome assembly for the eastern fence lizard, *S. undulatus, de novo* transcriptomes for *S. undulatus* encompassing multiple tissues and life stages, and improved draft genome assemblies from 34 additional *Sceloporus* species. In the final reference assembly, SceUnd1.0, the largest 11 scaffolds contain 92.6% (1.765 of 1.905 Gb) of the genome sequence; these 11 scaffolds likely represent the 6 macro- and 5 microchromosomes of *S. undulatus*, based on karyotype, genome size, BUSCO analysis, and synteny with other squamate genomes. The remaining small scaffolds may contain some chromosome segments that could not be assembled, misassembled regions, or duplicated genes.

In comparing the 3 levels of reference genome assemblies, we found that the first level using only the 10X Genomics and the SuperNova Assembly contained all, or very nearly all, of the protein-coding regions of the genome within its contigs (based on BUSCO and mapping of RNAseq and whole-genome resequencing data). By including the Hi-C data, the contiguity of the HiRise Assembly dramatically improved, joining contigs into chromosome-length scaffolds, but had minimal effect on mapping percentages for either RNAseq or WGS. The inclusion of the PacBio data in the final PBJelly Assembly to produce SceUnd1.0 closed some gaps but yielded a relatively small improvement after the already dramatic improvements from the Hi-C data.

While it is now becoming possible to obtain a reference genome assembly for almost any organism, the quality and cost of reference genome assemblies vary considerably depending on the technologies used. This presents researchers with an important question: what levels of sequencing effort and assembly quality are required for a particular ecological genomics study? Important factors that must be considered include the sequencing depth, sequence contiguity, and thoroughness of annotation. Our study demonstrates that the SuperNova Assembly was sufficient for mapping RNAseq and whole-genome resequencing, while the more expensive data from HiC and PacBio were necessary to achieve high-level continuity and chromosome-level scaffolding in the HiRise and PBJ Assemblies.

Genome assemblies of high quality and contiguity are critical for understanding organismal biology in a wide range of contexts that includes behavior, physiology, ecology, and evolution, on scales ranging from populations to higher-level clades. From RNAseq to ChIPseq (chromatin immunoprecipitation sequencing) and epigenetics, large-scale sequencing is rapidly becoming commonplace in ecological genomics to address fundamental questions of how organisms directly respond to their environment and how populations evolve in response to environmental variation. Many advanced molecular tools are typically reserved for traditional model organisms, but with the large foundation of ecological and physiological data available for *S. undulatus*, a high-quality reference genome opens the door for these molecular techniques to be used in this ecological model organism. For example, with the recent demonstration of CRISPR-Cas9 gene modification in a lizard, the brown anole [103], a genome reference will facilitate the application of gene drive technologies for functional genomic studies in *Sceloporus* lizards. This reference will provide a foundation for whole-genome studies to elucidate speciation and hybridization among closely related species utilizing low-coverage re-sequencing, or as a point of comparison with more distantly related species relative to the chromosomal inversions and large-scale genome architectural changes common in the clade. *Sceloporus undulatus* and other lizards in the genus *Sceloporus* exhibit evolutionary reversals in sexual size dimorphism and dichromatism and they have been used to demonstrate that androgens such as testosterone can inhibit growth in species (such as *S. undulatus*) in which females are the larger sex [19, 104–106]. This SceUnd1.0 chromosome-level genome assembly would support ChIPseq or *in silico* analyses to identify sex hormone response elements. In addition, this assembly will facilitate the identification of signatures of exposure to environmental stressors in both gene expression and epigenetic modification [107] to evaluate pressing questions on how climate change and invasive species affect local fauna. All of these uses for a chromosome-level genome assembly provide valuable extensions to ongoing work in the *Sceloporus* genus.

## Data Availability

All raw data are available on NCBI. The BioProject for the Genome Sequencing is PRJNA612440. The Whole Genome Shotgun project has been deposited at DDBJ/ENA/GenBank under the accession JAGXEY000000000. The assembly Sceloporus undulatus AU_SceUnd_v1.1 (a slightly updated version of SceUnd1.0 based on NCBI requirements) is version JAGXEY010000000, GenBank accession GCA_019175285.1. These NCBI BioProjects contain RNAseq data associated with this article: PRJNA371829, PRJNA437943, PRJNA629371, along with SRA SRR629640. The NCBI BioProject with the raw data for heterozygosity estimates associated with this article is PRJNA656311. All supporting data and materials are available in the *GigaScience* GigaDB database [108] and the Auburn University Scholarly Repository, AUora [109], including the following:

1. All 3 genome assemblies and their BUSCO results.
  - SuperNova assembly containing data from 10X Genomics Chromium: GenomeAssembly_SuperNova_Sceloporus_undulatus_pseudohap.fasta.gz
  - HiRise assembly containing the 10X Genomics data with the addition of the Hi-C data: GenomeAssembly_HiRise_Sceloporus_undulatus.fasta.gz
  - PBJelly Assembly (SceUnd1.0) containing the 10X Genomics data and the Hi-C data, with the addition of PacBio data: GenomeAssembly_SceUnd1.0_PBJELLY.fasta.gz
2. Tissue-Embryo Transcriptomes and annotation are provided as supplemental data.
  - TranscriptomeAssemblyAnnotation.zip folder containing
    - Transcriptome File: TranscriptomeAssembly_Tissues-Embryo_Trinity.fasta
    - Annotation File: TranscriptomeAssembly_Tissues-Embryo_Transdecoder.gff3
3. Truncated assembly used for the Funannotate annotation pipeline (SceUnd1.0_top24), and the annotation results are supplied as supplemental data.
  - SceUnd1.0_top24.fasta. This file contains only the longest 24 scaffolds and they have been renamed 1–24 from longest to shortest.
  - SceUnd1.0_top24_Annotation_FunnanotateResults.zip folder containing the following files:
    - SceUnd1.0_top24.gff3
    - SceUnd1.0_top24.proteins.fa
    - SceUnd1.0_top24.transcripts.fa
    - SceUnd1.0_top24.annotations.txt
    - SceUnd1.0_top24_CompiledAnnotation.csv
    - SceUnd1.0_top24.proteins.fa.report_EnsembleCombined.top.txt
4. The mitochondrial genomes and the annotation are provided as supplemental data.
  - MitoGenomeAssembly_Sceloporus_undulatus.fasta
  - MitoGenomeAssembly_Sceloporus_undulatus_Annotation.gff
5. The reference-based assemblies for the 34 Sceloporus species are provided as supplemental data.
  - GenomeAssemblies_34Sceloporus.tar.gz
  - Code for generating consensus sequences for each species: mkgenome_AW-AC.sh

## Additional Files

**Supplemental Results**.

## Abbreviations

BLAST: Basic Local Alignment Search Tool; bp: base pairs; BUSCO: Benchmarking Universal Single Copy Orthologues; ChIPseq: chromatin immunoprecipitation sequencing; E90N50: N50 of the most highly expressed transcripts that represent 90% of the total normalized expression data; GATK: Genome Analysis Toolkit; Gb: gigabase pairs; HET SNP: heterozygote single-nucleotide polymorphism; INDEL: insertion/deletion; kb: kilobase pairs; KEGG: Kyoto Encyclopedia of Genes and Genomes; L50 (L90): The smallest number of scaffolds that make up 50% (90%) of the total assembly length; LINE: long interspersed nuclear element; LTR: long terminal repeat; Mb: megabase pairs; mtDNA: mitochondrial DNA; N50 (N90): The contig or scaffold length such that the sum of the lengths of all scaffolds of this size or larger is equal to 50% (90%) of the total assembly length; NCBI: National Center for Biotechnology Information; ORF: open reading frame; PacBio: Pacific Biosciences; QC: quality control; RIN: RNA Integrity Number; RNAseq: RNA sequencing; SceUnd1.0: *Sceloporus undulatus* genome assembly including data from 10X Genomics Chromium library with Illumina sequencing, Hi-C library with Illumina sequencing, and PacBio sequencing assembled using the program PBJelly. Also referred to as the PBJelly assembly; SceUnd1.0_top24: *Sceloporus undulatus* genome assembly including only the longest 24 scaffolds from SceUnd1.0; SINE: short interspersed nuclear element; SNP: single-nucleotide polymorphism; SRA: Sequence Read Archive; tRNA: transfer RNA; WGS: whole-genome sequencing.

## Conflict of Interest

The authors declare that they have no conflict of interest.

## Funding

This work was supported by National Science Foundation Graduate Research Fellowship Program (DGE 1414475 to A.C.; DGE 1255832 to A.P.S.); National Science Foundation BCS-1554834 to GHP; National Science Foundation IOS-PMB 1855845 to A.D.L.; National Science Foundation IOS-1456655 to T.L.; Clemson University lab funds to M.S.; Georgia Southern Startup Funds to C.L.C.; University of Virginia start-up funding to R.M.C.; Hatch Multistate W3045 project no. NJ17240 to H.P.J.A.; Grant for Postdoctoral Interdisciplinary Research in the Life Sciences from the School of Life Sciences at Arizona State University to M.T.; Auburn University Start-up Funds to T.S.S.

## Authors’ Contributions

A.K.W.: Data curation; Formal analysis; Investigation; Validation; Visualization; Writing—original; Writing—review & editing

R.S.T.: Conceptualization; Data curation; Formal analysis; Investigation; Validation; Visualization; Writing—review & editing

M.B.G.: Data curation; Formal analysis; Investigation; Validation; Visualization; Writing—original; Writing—review & editing

D.S.W.: Data curation; Formal analysis; Software; Validation; Visualization; Writing—original; Writing—review & editing

A.D.C.: Data curation; Formal analysis; Methodology; Software, Validation, Visualization; Writing—review & editing

D.Y.S.: Formal analysis; Software; Writing—original; Writing—review & editing

R.L.K.: Methodology; Formal analysis; Writing—original draft; Writing—review & editing

A.P.S.: Formal analysis; Writing—original draft; Writing—review & editing

C.L.C.: Conceptualization; Data Curation; Investigation; Funding Acquisition; Writing—review & editing

G.H.P.: Funding acquisition; Supervision, Writing—review & editing

M.T.: Data curation; Formal analysis; Methodology; Funding acquisition; Writing—original draft; Writing—review & editing

T.L.: Conceptualization; Funding acquisition; Resources; Writing—review & editing

K.K.: Conceptualization; Funding acquisition; Resources; Writing—review & editing

M.W.S.: Resources; Funding Acquisition; Writing- review & editing

A.D.L.: Conceptualization; Data curation; Funding acquisition; Methodology; Writing—original; Writing—review & editing

M.J.A.: Conceptualization; Funding acquisition; Writing—review & editing

M.E.G.: Conceptualization; Writing—review & editing

H.P.J.A.: Investigation; Funding acquisition; Writing—review & editing

R.M.C.: Conceptualization; Funding acquisition; Investigation; Writing—review & editing

T.S.S.: Conceptualization; Data curation; Funding acquisition; Formal analysis; Investigation; Project Administration; Resources; Supervision; Writing—original; Writing—review & editing.

All authors have read and approved the final version of the manuscript.

## Supplementary Material

giab066_GIGA-D-20-00171_Original_Submission

giab066_GIGA-D-20-00171_Revision_1

giab066_GIGA-D-20-00171_Revision_2

giab066_Response_to_Reviewer_Comments_Original_Submission

giab066_Response_to_Reviewer_Comments_Revision_1

giab066_Reviewer_1_Report_Original_Submission Jeramiah J Smith -- 7/31/2020 Reviewed

giab066_Reviewer_1_Report_Revision_1 Jeramiah J Smith -- 5/25/2021 Reviewed

giab066_Reviewer_2_Report_Original_Submission Shanlin Liu -- 8/4/2020 Reviewed

giab066_Reviewer_2_Report_Revision_1 Shanlin Liu -- 5/5/2021 Reviewed

giab066_Supplemental_File
