# Supplementary material for: A chromosome-level genome assembly for the eastern fence lizard (Sceloporus undulatus), a reptile model for physiological and evolutionary ecology
Source: Gigascience. 2021 Oct 1;10(10):giab066. doi: 10.1093/gigascience/giab066 (PMC8486681; doi:10.1093/gigascience/giab066)
Supplement: giab066_GIGA-D-20-00171_Revision_1 [file giab066_giga-d-20-00171_revision_1.pdf]

GigaScience

A chromosome-level genome assembly for the Eastern fence lizard (Sceloporus undulatus), a reptile model for physiological and evolutionary ecology

--Manuscript Draft--

**Manuscript Number:**	GIGA-D-20-00171R1
**Full Title:**	A chromosome-level genome assembly for the Eastern fence lizard (Sceloporus undulatus), a reptile model for physiological and evolutionary ecology
**Article Type:**	Data Note

Funding Information:

National Science Foundation (DGE 1414475)	Ms. Amanda D. Clark
National Science Foundation (DGE 1255832)	Ms. Alexis P. Sullivan
National Science Foundation (BCS-1554834)	Dr. George H. Perry
National Science Foundation (1855845)	Dr. Adam D. Leaché
National Science Foundation (1456655)	Dr. Tracy Langkilde
Clemson University (Start-up Funds)	Dr. Michael W. Sears
Georgia Southern Univsersity (Start-up Funds)	Dr. Christian L. Cox
University of Virginia (US) (Start-up Funds)	Dr. Robert M. Cox
Auburn University (Start-up Funds)	Dr. Tonia S Schwartz
School of Life Sciences at Arizona State University (Postdoctoral Interdisciplinary Research in the Life Sciences)	Dr. Marc Tollis
Hatch Multistate W3045 (NJ17240)	Dr. Henry B. John-Alder

Abstract:

High-quality genomic resources facilitate population-level and species-level comparisons to answer questions about behavioral ecology, morphological and physiological adaptations, as well as the evolution of genomic architecture. Squamate reptiles (lizards and snakes) are particularly diverse in characteristics that have intrigued evolutionary biologists, but high-quality genomic resources for squamates are relatively sparse. Lizards in the genus Sceloporus have a long history as important ecological, evolutionary, and physiological models, making them a valuable target for the development of genomic resources. We present a high-quality chromosome-level reference genome assembly, SceUnd1.0, (utilizing 10X Genomics Chromium, HiC, and PacBio data) and tissue/developmental stage transcriptomes for the Eastern Fence Lizard, Sceloporus undulatus . We performed synteny analysis with other available squamate chromosome-level assemblies to identify broad patterns of chromosome evolution including the fusion of micro- and macrochromosomes in S. undulatus . Using this new S. undulatus genome assembly we conducted reference-based assemblies for 34 other Sceloporus species to improve draft nuclear genomes assemblies from 1% coverage to 43% coverage on average. Across these species, typically >90% of reads mapped for species within 20 million years divergence from S. undulatus , this dropped to 75% reads mapped for species at 35 million years divergence. Finally we use RNAseq and whole genome resequencing data to compare the three assemblies as references, each representing an increased level of sequencing, cost and assembly efforts: Supernova Assembly with data from10X Genomics Chromium library; HiRise Assembly that added data from HiC library; and PBJelly Assembly that added data from PacBio sequencing. We found that the Supernova Assembly contained the full genome and was a suitable reference for RNAseq, but the chromosome-level scaffolds provided by the addition of the HiC data allowed the reference to be used for other whole genome analysis, including synteny and whole genome association mapping analyses. The addition of PacBio data provided negligible gains. Overall, these new genomic resources provide valuable tools

Powered by Editorial Manager® and ProduXion Manager® from Aries Systems Corporation

	for advanced molecular analysis of an organism that has become a model in physiology and evolutionary ecology.
**Corresponding Author:**	Tonia S Schwartz, Ph.D.<br>Auburn University<br>Auburn, Alabama UNITED STATES
**Corresponding Author Secondary Information:**	
**Corresponding Author's Institution:**	Auburn University
**Corresponding Author's Secondary Institution:**	
**First Author:**	Aundrea K. Westfall, M.S.
**First Author Secondary Information:**	
**Order of Authors:**	Aundrea K. Westfall, M.S.
	Rory S. Telemeco
	Mariana B. Grizante
	Damien S. Waits
	Amanda D. Clark
	Dasia Y. Simpson
	Randy L. Klabacka
	Alexis P. Sullivan
	George H. Perry
	Christian L. Cox
	Robert M. Cox
	Matthew E. Gifford
	Henry B. John-Alder
	Michael W. Sears
	Michael J. Angilletta
	Marc Tollis
	Adam D. Leaché
	Tracy Langkilde
	Kenro Kusumi
	Tonia S Schwartz, Ph.D.
**Order of Authors Secondary Information:**	
**Response to Reviewers:**	Dear Editors and Reviewers,<br><br>Thank you for the review of our manuscript. We found the reviews insightful and responding to the comments have allowed us to improve the manuscript considerably. Below we have addressed every comment in Blue, and added or edited text in Blue italics, and when we have made changes in the manuscript we indicate the page and line numbers as well as use tracked changes in the manuscript. We think you will find these revisions have improved the manuscript.<br><br>Thank you,<br>Tonia<br><br>Reviewer #1: The manuscript "A chromosome-level genome assembly for the Eastern fence lizard (Sceloporus undulatus), a reptile model for physiological and evolutionary

ecology" by Westfall et al, reports a genome assembly that is likely to be of use to labs working in this and other reptilian systems. There are a few aspects of the presentation that are a bit confusing or are otherwise in need of revision, but I suspect these can be resolved. Specific comments are outlined below.

1) It in the context of the way the manuscript is laid out it would have been interesting to see the results of a PacBio only assembly, and/or an assembly that intergrated PacBio at an earlier stage, similar to the VGP assembly pipeline (https://vertebrategenomesproject.org/phase-one). Given the timescale of resource development and use of DoveTail (see also comment 2) I can understand why the reported approach was used, though it may not have been optimal.

Response: Thank you for this comment. We agree that comparing different combinations of the order in which we used the data types would be interesting. Unfortunately, because of how our current data was collected these would not be valid comparisons, particularly because the low level of coverage with the PacBio reads were collected specifically to complement the previous assembly and not sufficient for de novo assembly.

2) The use of HiRise (Dovetail) in the assembly pipeline raises some issues with reproducibility as that program is maintained as closed source code. As such it will be impossible for anyone to independently replicate the published assembly using the same methods reported in the paper. This may change in the future if the code is released, and I encourage the authors to request its release. If this request is not granted the authors should make sure to include the software version used for this assembly and all relevant assembly/filtration parameters, as well as a .agp (or similar) file that relays mapping evidence and weights that were used in the scaffolding process.

Response: As correctly indicated by the reviewer the HiRise Pipeline is proprietary to DoveTail Genomics, who did the sequencing and assembly for that portion of this project. Unfortunately, upon contacting DoveTail Genomics they notified us that the production files older than 3 years are purged from their system so the specific run files are not available. Therefore, we are not able to provide those requested files. We have included the HiRise version number and parameters in the main text (see below) for the readers to evaluate.

Page 5, line 127, text added:
“The data from both Hi-C and the 10X Genomics were used for assembly in the HiRise software (v2.1.3-5ce4af34ac25) pipeline at DoveTail Genomics. The pipeline excludes contigs/scaffolds < 1kb and only uses MQ>50 reads for scaffolding. The reads were aligned with a modified SNAP pipeline.”

3) It is unclear to me if the assignment of the sex chromosome is valid. It appeals that the only evidence used to support this is homology to the anole sex chromosome. In a lineage with variable sex chromosomes it would seem that this would provide scant evidence in favor of a particular chromosomes being the sex chromosome. Is there any other information that can be leveraged here? If not I would recommend dropping this section and the paragraph immediately preceeding the discussion.

Response: Thank you for this comment, we now see how this section can be clarified, and we have added text to clarify this point (see below). We have included additional information in this section for why we think this approach would likely identify the sex chromosome. But, we have also added “putative” in multiple places including Figure 5 to emphasize that this finding needs to be empirically confirmed. The paragraph above the discussion now describes the results from an additional, separate synteny analysis that further supports our original inference that the fourth predicted microchromosome is the putative X chromosome.
Page 9, line 266, the following text has been added to the paragraph describing the annotation of the putative X chromosome:
“These heteromorphisms are likely the result of other chromosomes’ fusions to the X, as Sceloporus are among the large portion of iguanian lizards with conserved sex

Powered by Editorial Manager® and ProduXion Manager® from Aries Systems Corporation

chromosomes, and another Sceloporus species within the same broad 2n=22 radiation, Sceloporus malachiticus, has an X chromosome homologous to the green anole X, but fused to several microchromosomes [68]. Given the observed homology, we used known X chromosome genes from the green anole to identify the scaffold likely representing the X chromosome within S. undulatus independently from other synteny analysis."
Page 9, line 279:
"This result, that the fourth predicted microchromosome is the putative X chromosome, is further supported by a separate synteny analysis described below"
Page 14, line 457, we have edited this text in the last paragraph above the discussion to read:
"These synteny results further support that the fourth largest microchromosome in the SceUnd1.0 assembly is syntenic to the anole X chromosome (Figure 3, Figure 5). However, it is not syntenic to the python X chromosome, which is syntenic to the Z chromosome in other snakes. The tegu sex chromosome has not been identified. Based on the blast hits from the anole X-linked genes and this synteny analysis we define the fourth largest microchromosome in the SceUnd1.0 assembly as the putative X chromosome, but functional data are needed to confirm this assignment."

We have also edited the paragraph at the top of page 14 that further discusses the fusion and fission of chromosomes in squamates.

4) The presentation of data from the 34 additional species could also use some shoring up, particularly given the fact that these mostly rely on reduced representation data that should subsample ~2% of the genome (notably, the guess of 2% may be wrong here, can the authors provide an estimate). How much overlap is there in the physical regions sampled in each of the 34 species? Is it nearly the same 2% or are they sampling wildly different intervals? This is likely to be of interest to several groups that are using reduced representation sequencing for comparative studies. The new genome assembly uniquely positions the authors to address this and they seem to already have the necessary data in hand.

Response: We have included additional analyses for the reader to better understand the overlap among the 34 Sceloporus reference-based assemblies. We used bedtools to count (1) the number of bases that have coverage by a specific number of reference-based genome assemblies (1 to 34 of the assemblies), and (2) the accumulating fraction of the genome that is covered by 1 to 34 reference-based assemblies. We provide these as bar graphs in Supplemental Figure 4 (Figure S4). We also provide an example of how these data can be used by focusing on one gene of interest to our group, IGF1. Using the coordinates in the annotation in the .gff3 from the SceUnd1.0 we pulled out the exons for the target gene, IGF1. These were aligned and visualized in Geneious. We found 16 of the 34 species had >75==% coverage across the protein coding region of this gene, 24 of them had >50% coverage. This is sufficient coverage to calculate estimates of genetic variation, selection analysis, and gene tree comparisons.

Page 13, Line 421 we edited the text to read:
"However, together these draft genomes contain a substantial amount of data that can be used for comparative genomic analyses. Figure S4 demonstrates the overlap in coverage of SceUnd1.0 by the reference-based genome assemblies. These distributions estimate that 50% of the genome would be covered by a subset of 16 species. Focusing on one individual gene of interest to our group, IGF1, we found that 16 of the 34 species had >75% coverage across the protein coding region of this gene and 24 of 34 had >50% coverage (Figure S4). Therefore, this dataset should prove useful for analyses of protein and gene sequence evolution to understand behavioral ecology, physiology, developmental biology, and more."

5) Related to this, for cases where substantially more than 2% of the genome is corrected. How is this distributed? In repetitive elements? Single-copy sequence? And why does this deviate from the expected ~2% in the cases that is does (e.g. 44% in one case).

Powered by Editorial Manager® and ProduXion Manager® from Aries Systems Corporation

Response: The 2% estimate is based on in silico experiments. It seems there was considerable by-catch in much of the reduced-representation sequencing, which is normally filtered out when those reduced representation data are being analyzed. With this by-catch, we were able to recover a reasonable amount (20-40%) of the genome sequence at low (1-3X) coverage for most species. We plotted the distribution of coverage across the genomes for each species to demonstrate the low-level coverage (Figure S3).

Page 12, Line 402: We have edited to the text in this section to the following "It seems there was a considerable amount of by-catch in much of the reduced-representation sequencing that is normally filtered out when those reduced representation data are being analyzed. For the species with ~5Gb of sequencing data, we improved the genome coverage from an average of 1.23% to an average of 44.4% coverage at low depth (1-3X) (Figure S3). For S. occidentalis with ~ 41Gb of data, coverage improved from 61.0% to 88.7% (Table 7) at an average depth of ~ 20X (Figure S3)."

6) The authors cite reference 17 in support of the need to sample additional squamates. This reference focuses on repetitive elements, and is therefore notable that the authors do not report and data on the distribution or density of repeats. An analysis of repeat content/classification was likely done as part of the annotation pipelines and I think a brief summary would be a welcome addition to the report.

Response: Thank you for this suggestion. We have conducted a repeat analysis and have included it as an additional text section in the manuscript starting on Page 9, as well as a figure (Figure 3) and a supplementary table (Table S4) that describe the distribution of repeat elements in S. undulatus with an evolutionary contrast to A. carolinensis.

Minor comments:
Line 102 - the species name is misspelled
Response: Thank you for catching this typo, it is now corrected.

Line 381 - species name is not italicized
Response: Thank you for catching this typo, it is now corrected.

Reviewer #2: The authors represented us a genome assembly of Sceloporus undulates, which utilized three sequencing technologies including 10X linked reads, Hi-C data, and long reads from the PacBio sequencing platform. I totally agree with the authors to one of their main conclusions that the SuperNova assembly was sufficient for mapping RNAseq and whole-genome resequencing. Although such a conclusion could have long been recognized, it is helpful that the authors can prove it using this new dataset. In addition, a high-quality genome of S. undulates is a good addition to what we have for the squamate genomic data. Overall the manuscript is well developed. However, there are a couple of points that the authors should acknowledge and discuss:

Major:

(1) Please provide several important assessments to validate the genome assembly accuracy. For instance, genome size should be estimated to check whether the assembly can represent the entire genomic info or contains redundancies; A GC-depth plot should be included to demonstrate whether the assembly contains contaminations from other species; Contig N50 (different form scaffold N50) should be added to demonstrate the assembly contiguity level.

Response: Thank you for these suggestions. We have now provided citations for genome size estimates for related species for comparison, as well as a GC-depth plot to test for contamination from other species, which appears negligible. We added Contig N50 to Table 1.

Page 5-6, starting on line 153, we have added the following text:

Powered by Editorial Manager® and ProduXion Manager® from Aries Systems Corporation

	"Estimated genome size of the closely related species Sceloporus occidentalis is 2.36GB based on fulcan densiometry. Assuming S. undulatus is similar, the 1.9GB of sequence in our SceUnd1.0 assembly is likely either missing some data, or repeat regions have been condensed, creating redundancies. To assess the level of contamination in our SceUnd1.0 genome assembly we used Blobtools (v1) (Laetsch DR and Blaxter ML, 2017) workflow A that estimated contamination based on GC content difference that exist between taxa. In order to visualize depth by GC content for taxa represented in the assembly, we created a blobDB using a BAM file to infer coverage, sequence similarity hits based on the DIAMOND blast, and the SceUnd1.0 assembly fasta file. Plots were produced for two taxonomic ranks, phylum and order, with taxonomic annotation based on the "bestsum" taxrule. The majority of the represented taxa in the assembly were annotated as belonging to Chordata (phylum level) and Squamata (order level). There is a smaller, but visible, proportion of reads that are associated with order Testudines, which is likely due to regions of sequence similarity across reptiles. Overall, the plot demonstrates negligible contamination of other taxa (Figure S1)."<br><br>(2) The authors claimed that they conducted reference-based assemblies for 34 Sceloporus species to improve draft nuclear genomes assemblies from 1% coverage to 43% coverage. This information is rather misleading. Firstly, De novo assembly can hardly represent a widely accepted method when dealing with the reduced presentation data (also known as RAD-Seq or GBS data). Therefore, such a comparison doesn't mean much. Secondly, the authors should tone down this part, as readers would expect to make use of them directly when the authors mentioned them as draft genome assemblies. However, I can hardly find a scenario in which those assemblies can be reused directly. I would choose the S. undulates genome as a reference, instead of any those "draft genome assemblies", to conduct, for example, resequencing-based population studies.<br><br>Response: The goal of the reference-based assemblies was not intended to be a comparison to the de novo assemblies to determine which is a better method, but rather to demonstrate improvement of the assemblies as a resource for the community. These reference-based assemblies are not necessarily intended to be used as References in of themselves. The reviewer is correct that SceUnd1.0 would be the best assembly to map to (we have removed that phrase of the sentence). Nonetheless, these draft genomes contain a significant amount of data that can be used for comparative genomic analyses. We have provided additional analyses to demonstrate the number of genomes that can be sampled for a particular fraction of the genome, see Figure S4. These analyses demonstrate that, for ~50% of the bases in the SceUnd1.0 assembly, 16 species would be represented. We also provide an example, focusing on one gene of interest to our group, IGF1, we found that 16 of the 34 species had >75% coverage across the protein coding region of this gene, and 24 of them had >50% coverage. This is sufficient coverage to calculate estimates of genetic variation, selection analysis, and gene tree comparisons.<br><br>Page 13, Line 421 we edited the text to read:<br>"However, these draft genomes contain a substantial amount of data that can be used for comparative genomic analyses. Figure S4 demonstrates the overlap in coverage of SceUnd1.0 by the reference-based genome assemblies. These distributions estimate that 50% of the genome would be covered by a subset of 16 species. Just focusing on one gene of interest to our group, IGF1, we found that 16 of the 34 species had >75% coverage across the protein coding region of this gene, and 24 of them had >50% coverage (Figure S4). Thereby, this dataset should prove useful for analyses of protein and gene sequence evolution to understand behavioral ecology, physiology, developmental biology, and more."<br><br>(3) the authors claimed that the addition of PB long reads data provided negligible gains. PBJelly mainly works for gap-closing, so there might be a significant improvement in Contig N50 value, which can only be confirmed after the authors provide such data. In addition, the authors could use some other software, such as this one (http://biorxiv.org/content/10.1101/831248v1.full), which claimed to perform ten times better than PBJelly.

Powered by Editorial Manager® and ProduXion Manager® from Aries Systems Corporation

Response: We have added the Contig N50 to Table 1. Thank you for the suggestion to use the program presented in the BioRXiv link. We do not intend to reassemble and redo the analyses for this manuscript as other manuscripts are already in preparation or have been submitted that have used the current SceUnd1.0 Assembly. But we will definitely consider using the reference program in future versions as we continue to improve further versions of this genome reference.

Minor:

(4) Line 131: it is better to provide the genome coverage information (for example, 40X) in the main text as well.

Response: Thank you for this suggestion, it has been incorporated.

(5) line 163: scaffolds as long as 7 MB might not be mentioned as tiny scaffolds.

Response: Thank you for this suggestion. We have changed “tiny” to “smaller”.

(6) Line 179 - 181: the authors may obtain useful information from this paper, 10.1093/gigascience/giy163, which demonstrates the potential problems regarding the 10X genome assembly - generate lots of gaps and produce redundancies.

Response: Thank you for bringing this reference to our attention. While it is an interesting paper, we are uncertain what action (if any) is being requested by this comment in the context of revising the manuscript.

(7) line 259: the authors may want to give some clues for how to improve the genome annotation. Plus, the authors may provide some details on why those missing BUSCO genes failed to be annotated, was it attributed to sequence errors which cause nonsense mutations? If so, genome polishing may correct them.

Response: The SceUnd1.0 has been submitted to NCBI and is in line for their annotation pipeline and then it will be moved into ENSEMBL. We suspect this pipeline would improve the annotation. As this genome assembly is already being used for multiple projects, with manuscript preparation, undoubtedly efforts will continue to improve upon this assembly. In terms of the missing BUSCOS, the BUSCOs were for Tetrapoda and it is possible some of the genes are truly missing in S. undulatus. Because there were more BUSCOs fragmented or missing from the predicted proteins (the annotation) than the actual genomic sequence itself, we attribute those to annotation errors, not errors in the assembly.

Page 8, line 255 we have edited this text to read:
“Because there were more BUSCOs fragmented or missing from the predicted proteins (the annotation) than the actual genomic sequence itself, we attribute those to annotation errors, not errors in the assembly, which suggests this first version of annotation can be improved.”

(8) Line 278: For the mitogenome assembly, there is a big chance that de novo RNAseq assembly can obtain whole or part of the mitochondrial genome. It won't be hard work, and it is interesting to examine whether reference-based mito-genome assembly can introduce some unexpected bias.

Response: We used the program MitoZ to de-novo assemble the mitochondrial genome using RNA-seq paired-end sequencing data from 18 individuals (of the same population). We used several techniques to go about performing this assembly:

1) Assembly for each individual using the Quick Assembly approach without a taxonomic constraint
2) Assembly for each individual using the Multi-Kmer approach without a taxonomic constraint
3) Assembly for each individual using the Quick Assembly approach with a taxonomic constraint (Phrynosomatidae)
4) Assembly for each individual using the Multi-Kmer approach with a taxonomic

constraint (Phrynosomatidae)
5) Assembly of all combined reads using the Quick Assembly approach
6) Assembly of all combined reads using the Multi-Kmer approach
7) Assembly of only those reads that mapped to the Sceloporus occidentalis mitochondrial genome using the Quick Assembly approach
8) Assembly of only those reads that mapped to the Sceloporus occidentalis mitochondrial genome using the Multi-Kmer approach

None of these approaches recovered the full mitochondrial genome (each resulted in missing protein coding genes, tRNA, and rRNA). Further, different approaches sometimes selected different scaffolds as those pertaining to the mitochondrial genome. These inconsistencies and lack of data led us to the decision to retain our approach described in the original manuscript submission, wherein we mapped the raw reads to the Sceloporus undulatus mitochondrial genome, aligned this genome with Anolis carolinensis, and transferred annotations.

Page 10, line 327, we add the following sentence to the manuscript.
"While this genome is useful for understanding sequence variation and comparative genomics and phylogenetic analyses, this mitochondrial genome should not be used for examination of mitochondrial genome structure."

As for extracting the genomes from the other 34 Sceloporus species for examination of species relationships, this is not within the scope of this study and is an objective being pursued by other labs using more appropriate datasets.

(9) Line 318: can the taxonomic uncertainty be resolved using the resequencing data, e.g. you can obtain their mito-genomes via software like Novoplasty, MitoZ, et al.

Response: This question of taxonomic uncertainty of the populations in this region is currently being addressed in more depth by other research labs as is outside of the scope of this manuscript.

(10) Line 328: Can FLAGSTAT work on sam files? Or do you mean bam files?

Response: Yes FLAGSTAT can work on sam files.

(11) Line 345 - 347: How to evaluate the correctness of those increased SNPs?

Response: We are not exactly sure what this question is asking. We did not actually call SNPs with the WGS database, but used the theoretical HET SNP sensitivity metric to estimate the utility of each assembly for calling SNPs. The full WGS dataset is part of a much bigger study across S. undulatus populations and will be published at a later date. We try to further clarify the theoretical HET SNP sensitivity metric and how we are using it as a metric for useability of the assemblies in the main text. We have added the following text.

Page 11, Line 362:
"The CollectWgsMetrics tool from the Picard Toolkit [85] was used to calculate genome-wide coverage of the mapped reads for each individual and assembly, and theoretical HET SNP sensitivity (a metric based on coverage and base quality distribution that estimates probability of calling a true heterozygote SNP) as a way to predict the utility of each assembly as a reference for calling SNPs at high a low coverage. "

Line 375: "This may be due to repetitive regions being added to the assembly by the PacBio data making it slightly less mappable. "

(12) Line 370: 41Gb -> 40.8Gb or ~ 41 Gb
Response: Thank you for catching this typo, it is now corrected.

(13) Line 397: please clarify the criteria and methods that produce those markers of 1,000 bp in length.
Response: We have clarified this method with the following statement:

	Line 436" "The SceUnd1.0 scaffolds representing the 11 putative chromosomes were each divided into 1000 bp-long sequences that excluded gapped regions to serve as markers."<br><br>(14) Line 400: > 80% identity?<br>Response: Thank you for catching this typo, it is now corrected.<br><br>(15) Line 443: PBJelly cannot aid in chromosome-level assembly.<br><br>Response: Good point. We have now rephrased this to be:<br><br>Now Line 487<br>"Our study demonstrates that the SuperNova Assembly was sufficient for mapping RNAseq and whole genome resequencing, while the more expensive data from HiC and PacBio were necessary to achieve high-level continuity and chromosome-level scaffolding in the HiRise and PBJ Assemblies."
**Additional Information:**	
**Question**	**Response**
Are you submitting this manuscript to a special series or article collection?	No
**Experimental design and statistics**<br><br>Full details of the experimental design and statistical methods used should be given in the Methods section, as detailed in our Minimum Standards Reporting Checklist. Information essential to interpreting the data presented should be made available in the figure legends.<br><br>Have you included all the information requested in your manuscript?	Yes
**Resources**<br><br>A description of all resources used, including antibodies, cell lines, animals and software tools, with enough information to allow them to be uniquely identified, should be included in the Methods section. Authors are strongly encouraged to cite Research Resource Identifiers (RRIDs) for antibodies, model organisms and tools, where possible.<br><br>Have you included the information requested as detailed in our Minimum Standards Reporting Checklist?	Yes

**Availability of data and materials**<br><br>All datasets and code on which the conclusions of the paper rely must be either included in your submission or deposited in publicly available repositories (where available and ethically appropriate), referencing such data using a unique identifier in the references and in the “Availability of Data and Materials” section of your manuscript.<br><br>Have you have met the above requirement as detailed in our Minimum Standards Reporting Checklist?	No
If not, please give reasons for any omissions below.<br><br>as follow-up to "**Availability of data and materials**<br><br>All datasets and code on which the conclusions of the paper rely must be either included in your submission or deposited in publicly available repositories (where available and ethically appropriate), referencing such data using a unique identifier in the references and in the “Availability of Data and Materials” section of your manuscript.<br><br>Have you have met the above requirement as detailed in our Minimum Standards Reporting Checklist?<br><br>"	The raw RNAseq and whole genome sequencing data are in NCBI SRA. The assemblies have not yet been uploaded in to NCBI, we would like to submit our assemblies to GigaDB to make them assessable for review.

Powered by Editorial Manager® and ProduXion Manager® from Aries Systems Corporation

Manuscript

Click here to access/download;Manuscript;Westfall_etal_ScelopGenome_Revi

For submission to **GIGASCIENCE** as a *DATA NOTE*

A chromosome-level genome assembly for the Eastern fence lizard (*Sceloporus undulatus*), a reptile model for physiological and evolutionary ecology

Aundrea K. Westfall[1], Rory S. Telemeco[1,2], Mariana B. Grizante[3], Damien S. Waits[1], Amanda D. Clark[1], Dasia Y. Simpson[1], Randy L. Klabacka[1], Alexis P. Sullivan[4], George H. Perry[4,5,6], Michael W. Sears[7], Christian L. Cox[8,9], Robert M. Cox[10], Matthew E. Gifford[11], Henry B. John-Alder[12], Tracy Langkilde[4], Michael J. Angilletta Jr.[3], Adam D. Leaché[13,14], Marc Tollis[3,15], Kenro Kusumi[3], and Tonia S. Schwartz[1, §]

[1] Department of Biological Sciences, Auburn University, Auburn, AL 36849
[2] Department of Biology, California State University Fresno, Fresno, CA 93740
[3] School of Life Sciences, Arizona State University, Tempe, AZ 85287
[4] Department of Biology, Pennsylvania State University, University Park, PA 16802
[5] Department of Anthropology, Pennsylvania State University, University Park, PA 16802
[6] Huck Institutes of the Life Sciences, Pennsylvania State University, University Park, PA 16802
[7] Department of Biological Sciences, Clemson University, Clemson, SC 29634
[8] Department of Biology, Georgia Southern University, Statesboro, GA 30460
[9] Department of Biological Sciences, Florida International University, Miami, FL 33199
[10] Department of Biology, University of Virginia, Charlottesville, VA 22904
[11] Department of Biology, University of Central Arkansas, Conway, AR 72035
[12] Department of Ecology, Evolution, and Natural Resources, Rutgers University, New Brunswick, NJ 08901
[13] Department of Biology, University of Washington, Seattle, WA 98195
[14] Burke Museum of Natural History and Culture, University of Washington, Seattle, WA 98195
[15] School of Informatics, Computing, and Cyber Systems, Northern Arizona University, Flagstaff, AZ 86011

[§]**Author for Correspondence**: Tonia S. Schwartz, Department of Biological Sciences, Auburn University, Auburn, AL 36849. *Email:* tschwartz@auburn.edu *phone:* 334-844-1555

Running Head: Eastern Fence Lizard Genome

Abstract

Background: High-quality genomic resources facilitate investigations into behavioral ecology, morphological and physiological adaptations, and the evolution of genomic architecture. Lizards in the genus *Sceloporus* have a long history as important ecological, evolutionary, and physiological models, making them a valuable target for the development of genomic resources. **Findings:** We present a high-quality chromosome-level reference genome assembly, SceUnd1.0, (utilizing 10X Genomics Chromium, HiC, and PacBio data) and tissue/developmental stage transcriptomes for the Eastern Fence Lizard, *Sceloporus undulatus*. We performed synteny analysis with other snake and lizard assemblies to identify broad patterns of chromosome evolution including the fusion of micro- and macrochromosomes. We also used this new assembly to improve genome assemblies for 34 additional *Sceloporus* species, improving these assemblies from 1% coverage to 43% coverage on average. Finally, we used RNAseq and whole-genome resequencing data to compare three assemblies, each representing an increased level of cost and effort: Supernova Assembly with data from 10X Genomics Chromium; HiRise Assembly that added data from HiC; and PBJelly Assembly that added data from PacBio sequencing. We found that the Supernova Assembly contained the full genome and was a suitable reference for RNAseq and SNP calling, but the chromosome-level scaffolds provided by the addition of HiC data allowed synteny and whole genome association mapping analyses. The subsequent addition of PacBio data provided negligible gains. **Conclusions:** These new genomic resources provide valuable tools for advanced molecular analysis of an organism that has become a model in physiology and evolutionary ecology.

Keywords: genome, transcriptome, squamate, reptile

Data Description

Context

Genomic resources, including high-quality reference genomes and transcriptomes, facilitate comparisons across populations and species to address questions ranging from broad-scale chromosome evolution to the genetic basis of key adaptations. Squamate reptiles, the group encompassing lizards and snakes, have served as important models in ecological and evolutionary physiology due to their extensive metabolic plasticity [1]; diverse reproductive modes including obligate and facultative parthenogenesis [2]; repeated evolution of placental-like structures [2,3]; shifts among sex-determining systems, with XY, ZW, and temperature-dependent systems represented often in closely related species [4,5]; loss of limbs and elongated body forms [6]; and the ability to regenerate tissue [7,8]

Despite having evolved greater phylogenetic diversity than mammals and birds, two major vertebrate groups with extensive genome sampling, genomic resources for squamates remain scarce and assemblies at the chromosome-level are even more rare [7,9–13]. While squamates are known to have a level of karyotypic variability similar to that of mammals [14], the absence of high-quality genome assemblies has led to their exclusion from many chromosome-level comparative genome analyses. In comparative studies, non-mammalian amniotes are often represented only by the chicken, which is divergent from squamate reptiles by almost 280 million years [15], or the green anole (*Anolis carolinensis*), whose genome is only 60% assembled into chromosomes and is lacking assembled microchromosomes [14,16]. However, recent analyses have identified key differences that distinguish the evolution of squamate genomes from patterns found in mammals and birds [17], underscoring the need for additional high-quality genome assemblies for lizards and snakes. The development of additional squamate genomes within and across lineages will facilitate investigations of the genetic basis for many behavioral, morphological, and physiological adaptations in comparisons of organisms from the population up to higher-order taxonomic ranks.

Our goal was to develop high-quality genomic and transcriptomic resources for the spiny lizards (*Sceloporus*) to further our ability to address fundamental ecological and evolutionary questions within this taxon, across reptiles, and across vertebrates. The genus *Sceloporus* includes approximately 100 species extending throughout Central America, Mexico, and the United States [18]. Researchers have used *Sceloporus* for decades as a model system in the study of physiology [19,20], ecology [21,22], reproductive ecology [23–25], life history [26–28], and evolution [25,29–31]. The long history of research on *Sceloporus* species, applicability across multiple fields of biology, and the extensive diversity of the genus makes this an ideal group to target for genomic resource development.

We focus on the Eastern fence lizard, *Sceloporus undulatus*, which is distributed in forested habitats east of the Mississippi River [32]. Recently, *S. undulatus* has been the focus of studies on the development of sexual size dimorphism [33,34], as well as experiments testing the effects of invasive species [35–37] and climate change [22,38–40] on survival and reproduction as a model to better understand the consequences of increasing anthropogenic disturbance. The development of genomic resources for *S. undulatus,* particularly a high-quality genome assembly, will support its role as a model species for evolutionary and ecological physiology, and will have immediate benefits for a broad range of comparative studies in physiology, ecology, and evolution.

To this end, we developed a high-quality chromosome-level reference genome assembly and transcriptomes from multiple tissues for *S. undulatus*. We apply this genome reference to datasets on three scales: (1) to address how assembly quality influences mapping of RNAseq (RNA sequencing) and low coverage whole-genome sequence data; (2) to improve upon the genomic resources for the *Sceloporus* genus by creating reference-based assemblies of draft genomes for 34 other *Sceloporus* species; and (3) to draw broad comparisons in chromosome structure and conservation with other recently published squamate chromosome-level genomes through large-scale synteny analysis.

Methods and Analyses

Sequencing and assembly of the Sceloporus undulatus genome

Genome sequence data were generated from two male *S. undulatus* collected at Solon Dixon Forestry Education Center, in Andalusia, Alabama (31°09’49”N, 86°42’10”W). The animals were euthanized and tissues were dissected, snap-frozen in liquid nitrogen, and stored at -80°C. Procedures were approved by the Pennsylvania State University Institutional Animal Care and Use Committee (Protocol# 44595-1).

We developed three *S. undulatus* genome assemblies using increasingly more data with correspondingly greater cost: (1) a SuperNova assembly containing data from 10X Genomics Chromium, (2) a HiRise assembly containing the 10X Genomics data with the addition of Hi-C data, and (3) a PBJelly Assembly containing the 10X Genomics data and Hi-C data, and the addition of PacBio data. These assemblies are provided as supplemental files and their summary statistics are provided in Table 1.

In the fall of 2016, we sequenced DNA from snap-frozen brain tissue of a single juvenile male *S. undulatus* using 10X Genomics Chromium Genome Solution Library Preparation with SuperNova Assembly [41] through HudsonAlpha. The library was sequenced on one lane of Illumina HiSeqX

resulting in 774 million 150 base-pair (bp) paired-end reads that were assembled using the SuperNova pipeline. We refer to this assembly with 46X coverage as the SuperNova Assembly.

In the fall of 2017, we sequenced a second male (Figure 1) from the same population using a Hi-C library with Illumina sequencing through Dovetail Genomics prepared from blood, liver, and muscle tissue. We used this second individual because the remains from the individual used for SuperNova Assembly were insufficient for Hi-C library preparation, which required 100 mg of tissue. Dovetail Genomics developed two Hi-C libraries that were sequenced on an Illumina HiSeqX to produce 293 million and 289 million (total 582 million) 150 bp paired-end reads. The data from both Hi-C and 10X Genomics were used for assembly via the HiRise software (v2.1.3-5ce4af34ac25) pipeline at DoveTail Genomics. This pipeline excludes contigs/scaffolds < 1 kilobase pair (Kb) and only uses MQ>50 reads for scaffolding. The reads were aligned with a modified SNAP pipeline. We refer to this assembly with 4859X coverage, as the HiRise Assembly.

Finally, also in fall of 2017, DNA extracted from the second adult male was used by Dovetail Genomics to generate 1,415,213 PacBio reads with a mean size of 12,418.8 bp (range 50-82,539 bp). These PacBio data were used for gap-filling to further improve the lengths of the scaffolds of the HiRise Assembly using the program PBJelly [42]. We refer to this final assembly containing all three types of sequencing data as the PBJelly Assembly and the final SceUnd1.0 reference genome assembly.

For a visual comparison of our three *S. undulatus* assemblies and other squamate genomes, we graphed genome contiguity for these three assemblies with other squamate reptile genomes, building on the graph by Roscito et al. [43]. The *S. undulatus* SuperNova Assembly (containing only the 10X Genomics data) is as contiguous as the bearded dragon genome assembly (Figure 2a). The addition of the HiRise data brought a large increase in continuity. The HiRise and PBJelly *S. undulatus* Assemblies are nearly indistinguishable from each other and are among the most contiguous squamate genome assemblies to date (Figure 2a).

The SceUnd1.0 assembly contains 45,024 scaffolds (>850 bp, without gaps) containing 1.9 gigabase pairs (Gb) of sequence, with an N50 of 275 Megabase pairs (Mb). Importantly, 92.6% (1.765 Gb) of the assembled sequence is contained within the first 11 scaffolds. Chromosomal studies have determined that the *S. undulatus* karyotype is 2N = 22 with a haploid genome of N = 11 (six macrochromosomes + five microchromosomes; 6ma + 5mi) [31,44]. Sorting the top 11 scaffolds by size (Figure 2b) suggests that scaffolds 1-6 are the macrochromosomes (170-383 Mb in size) and scaffolds 7-11 are the five microchromosomes (13-52 Mb in size) (Figure 2b). These results suggest that the first 11 scaffolds represent the 11 chromosomes, although the assembly also produces 45,000 smaller scaffolds between 0.85Kb – 7Mb that may still contain relevant chromosomal segments that could not be assembled. Estimated genome size of the closely related

species *Sceloporus occidentalis* is 2.36 GB based on fulcan densiometry [14]. Assuming *S. undulatus* is similar, the 1.9GB of sequence in our SceUnd1.0 assembly is likely either missing some data, or repeat regions have been condensed, representing redundancies. To assess the level of contamination in our SceUnd1.0 genome assembly, we used Blobtools (v1) [45] workflow A that estimated contamination based on GC content differences that exist between taxa. To visualize depth by GC content for taxa represented in the assembly, we created a blobDB using a BAM file to infer coverage, sequence similarity hits based on the DIAMOND blast, and the SceUnd1.0 assembly fasta file. Plots were produced for two taxonomic ranks, phylum and order, with taxonomic annotation based on the "bestsum" taxrule. The majority of the represented taxa in the assembly were annotated as belonging to Chordata (phylum level) and Squamata (order level). There is a smaller, but visible, proportion of reads that are associated with order Testudines, which is likely due to regions of sequence similarity across reptiles. Overall, the plot demonstrates negligible contamination of other taxa (Figure S1).

To assess the completeness of our three genome assemblies, we utilized the BUSCO (Benchmarking Universal Single-Copy Orthologues) Tetrapoda dataset (3950 genes) [46,47]. For all three assemblies we found over 89% of BUSCO genes complete (Table 1) with only minor differences in BUSCO genes between the SuperNova, HiRise, and PBJelly Assemblies (89.5%, 90.2%, 90.9% complete). This suggests that the initial SuperNova Assembly captured nearly all of the genomic content despite having considerably shorter scaffolds (Table 1). The small increase in success with the more contiguous assemblies appears to result from a reduction in fragmented BUSCO genes with increasing data. In the SuperNova Assembly, 6.4% of BUSCO genes were present as fragments whereas only 5.5% and 5.0% were present as fragments in the HiRise and PBJelly Assemblies, respectively, thus explaining the 1.4% difference in complete BUSCO genes present. Interestingly, there was a 0.2% (i.e., 8 genes) increase in missing BUSCO genes from the SuperNova to the HiRise Assembly. In the PBJelly Assembly (SceUnd1.0), the BUSCO genes are almost all found on the largest 11 scaffolds (Figure 2c), as we would predict if those scaffolds correspond to chromosomes. Most of the BUSCO genes on the smaller scaffolds were duplicated. Even so, there are a small number of complete and fragmented BUSCO genes present on a handful of the tiny scaffolds (Figure 2c), suggesting that these scaffolds contain pieces of the chromosomes that were not properly assembled.

De novo assembly and annotation of the* Sceloporus undulatus *transcriptome

Samples used for the *de novo* transcriptome were obtained from three gravid females of *Sceloporus undulatus* collected in Edgefield County, South Carolina (33.7°N, 82.0°W) and transported to Arizona State University. These animals were maintained under conditions described in previous publications [48,49], which were approved by the Institutional Animal Care and Use Committee (Protocol #14-1338R) at Arizona State University. Approximately two days after laying eggs, each

lizard was euthanized by injecting sodium pentobarbital into the coelomic cavity. Whole brain and skeletal muscle samples were removed and placed in RNA-lysis buffer (mirVana miRNA Isolation Kit, Ambion) and flash-frozen. Additionally, three early-stage embryos from each clutch were dissected, pooled together, homogenized in RNA-lysis buffer, and also flash frozen.

Total RNA was isolated from the embryo and three tissue samples from each adult female (whole brain, skeletal muscle) using the mirVana miRNA Isolation Kit (Ambion) total RNA protocol. Samples were checked for quality on a 2100 Bioanalyzer (Agilent). One sample from each tissue was selected for RNAseq based on the highest RNA Integrity Number (RIN), with a minimum cutoff of 8.0. For each selected sample, 3 μg of total RNA was sent to the University of Arizona Genetics Core (Tucson, AZ) for library preparation with TruSeq v3 chemistry for a standard insert size. RNA samples were multiplexed and sequenced using an Illumina HiSeq 2000 to generate 100-bp paired-end reads. Publicly available raw Illumina RNAseq reads from *S. undulatus* liver (juvenile male) were also added to our dataset [50,51]. After removing adapters, raw reads from the four tissues were evaluated using FastQC (https://github.com/s-andrews/FastQC) and trimmed using Trimmomatic v-0.32 [52], filtering for quality score (≥Q20) and using HEADCROP:9 to minimize nucleotide bias. This procedure yielded 179,374,469 quality-filtered reads. Table 2 summarizes read-pair counts from whole brain, skeletal muscle, whole embryos, and liver.

All trimmed reads were pooled and assembled *de novo* using Trinity v-2.2.0 with default k-mer size of 25 [53]. From the final transcriptome, a subset of contigs containing the longest open reading frames (ORFs), representing 123,323 transcripts, was extracted from the *de novo* transcriptome assembly using TransDecoder v-3.0.0 (http://transdecoder.github.io) with homology searches against the databases UniProtKB/SwissProt [54] and PFAM [55]. The transcriptome was annotated using Trinotate v-3.0 (http://trinotate.github.io), which involved searching against multiple databases (as UniProtKB/SwissProt, PFAM, signalP, GO) to identify sequence homology and protein domains, as well as to predict signaling peptides. This pooled Tissue-Embryo Transcriptome and annotation are provided as supplemental files.

The most comprehensive transcriptome, obtained using reads from four tissues, consists of 547,370 contigs with an average length of 781.5 nucleotides (Table 2) — shorter than other assemblies because of the range of contig sizes that varied among datasets (1, 3 and 4 tissues; Table S1, Figure S2). The N50 of the most highly expressed transcripts that represent 90% of the total normalized expression data (E90N50) was lowest in the assembly based on one tissue (Table 2). To validate the *de novo* transcriptome data, trimmed reads from the 4 tissues used for RNA sequencing (brain, skeletal muscle, liver and whole embryos) were aligned back to the Trinity assembled contigs using Bowtie2 v2.2.6 [56]. From the 176,086,787 reads that aligned, 97% represented proper pairs (Table S2), indicating good read representation in the *de novo* transcriptome assembly. To assess quality and completeness of the assemblies, we first compared

the *de novo* assembled transcripts with the BUSCO Tetrapoda dataset, with BLAST+ v2.2.31 [57] and HMMER v3.1b2 [58] as dependencies. This procedure revealed that the *de novo* transcriptome assembly captured 97.1% of the expected orthologues (sum of completed and fragmented), a result comparable to the 97.8% obtained for the green anole transcriptome using 14 tissues [59] (Table 3). Next, nucleotide sequences of *de novo* assembled transcripts with the longest ORFs were compared to the protein set of *Anolis carolinensis* (AnoCar2.0, Ensembl) using BLASTX (evalue=1e-20, max_target_seqs=1). This comparison showed that 11,223 transcripts of *S. undulatus* have nearly full-length (>80%) alignment coverage with *A. carolinensis* proteins (Table S3). Predicted proteins of *S. undulatus* were also used to identify 13,422 one-to-one orthologs with proteins of *A. carolinensis* through reciprocal BLAST (evalue=1e-6, max_target_seqs=1). Table 4 summarizes the *de novo* transcriptome annotation results.

Genome Assembly Annotation

Using the 24 largest scaffolds of the SceUnd1.0 assembly (we refer to this set as SceUnd1.0_top24), we used the Funannotate v1.5.0 pipeline (https://github.com/nextgenusfs/funannotate) for gene prediction and functional annotation. Funannotate uses RNAseq data and the Tetrapoda BUSCO [46] dataset to train the *ab initio* gene prediction programs Augustus [60] and GeneMark-ET [61]. Evidence Modeler is used to generate the consensus from Augustus and GeneMark-ES/ET. In the training step, we used four raw RNAseq datasets described in Table 2 that contained a total of 68 sequenced libraries. tRNAscan-SE [62] was used to predict tRNA genes. Finally the genes were functionally annotated via InterProScan [63], eggNOG [64], Pfam [55] , UniProtKB [54], MEROPS [65], CAZyme, and GO ontology. We also used DIAMOND blastp [66] to compare the predicted proteins to ENSEMBL human, chicken, mouse, and green anole lizard databases (Supplemental files: SceUnd1.0_top24.gff3; SceUnd1.0_top24_CompliedAnnotation.csv). Our annotation pipeline predicted 54,149 genes, 15,472 of which were attributed meaningful functional annotation beyond “hypothetical protein”. Through BLAST of the predicted protein coding genes, we found 21,050 (39%) had hits in ENSEMBL. We then quantified the number of BUSCO genes identified in the predicted proteins from the Funannotate pipeline and found 79.1%, which corresponds to an 11.6% decrease from the number of complete BUSCO genes in the SceUnd1.0 genome assembly. Because there were more BUSCOs fragmented or missing from the predicted proteins (the annotation) than the actual genomic sequence itself, we attribute those to annotation errors, not errors in the assembly, which suggests this first version of annotation can be improved. SceUnd1.1, (a slightly updated version of SceUnd1.0 based on NCBI requirements) has been submitted to NCBI for annotation.

We used annotation and sequence homology to identify the X chromosome. Sex chromosomes are highly variable among *Sceloporus* species, and the genus appears to have evolved multiple

variations of XY systems [31]. However, some species, including *S. undulatus*, do not appear to have morphologically distinct sex chromosomes [67]. While the ancestral condition is heteromorphic chromosomes with a minute Y, many species within the genus demonstrate multiple sex chromosome heteromorphisms (i.e. multiple forms of the X chromosome) or have evolved indistinct sex chromosomes, such as the *undulatus* species group [18]. These heteromorphisms are likely the result of other chromosomes' fusions to the X, as *Sceloporus* are among the large portion of iguanian lizards with conserved sex chromosomes, and another *Sceloporus* species within the same broad 2n=22 radiation, *Sceloporus malachiticus*, has an X chromosome homologous to the green anole X, but fused to several microchromosomes [68]. Given this observed homology, we used known X chromosome genes from the green anole to identify the scaffold likely representing the X chromosome within *S. undulatus*. We blasted 16 X-linked genes from the green anole downloaded from Ensembl (AnoCar2.0: ACAD10, ADORA2A, ATP2A2, CCDC92, CIT, CLIP1, CUX2, DGCR8, FICD, MLEC, MLXIP, ORAI1, PLBD2, PUS1, TMEM119, ZCCHC8) [69,70] to SceUnd1.0. They almost exclusively map to the tenth largest scaffold, the fourth predicted microchromosome (Figure 2b), indicating that it is likely the X chromosome. The Y chromosome could not be independently identified from the assembly, most likely due to the homomorphic nature of *S. undulatus* sex chromosomes; higher sequence homology may have caused the Y chromosome to assemble with the X chromosome [31]. This result, that the fourth predicted microchromosome is the putative X chromosome, is further supported a separate synteny analysis described below.

Repeat Annotation and Evolutionary Analysis

To estimate the repetitive landscape of the *Sceloporus undulatus* genome, we modeled repeats *de novo* by running RepeatModeler v1.0.8 (http://www.repeatmasker.org) [71] on the SceUnd1.0 assembly. We then annotated repeats in the assembly using RepeatMasker v4.0.7 (http://www.repeatmasker.org) [72] with the *de novo* consensus repeat library. To estimate evolutionary divergence within repeat families in the *S. undulatus* genome, we generated repeat-family specific alignments and calculated the average Kimura-2-parameter divergence from consensus within each family, correcting for high mutation rates at CpG sites with the calcDivergenceFromAlign.pl RepeatMasker tool. We compared the divergence profiles of *S. undulatus* and *Anolis carolinensis* by completing parallel analyses. We annotated repeats in the *A. carolinensis* genome (AnoCar2.0) with RepeatMasker and the "anolis" repeat library from RepBase release 20170127 [73].

The *Sceloporus undulatus* assembly contained a diverse repertoire of repeats including transposable elements, the most abundant of which are the Long Interspersed Nuclear Elements (LINEs, Table S4) comprising ~15% of the genome. Relative proportions of LINEs, short interspersed nuclear repeats (SINEs), LTR (long terminal repeat) retrotransposons, and DNA

transposons were similar to those of *A. carolinensis*. The diversity of repeat elements in *S. undulatus* mirror that of the *Anolis* genome [74], as well as that of other squamates [17]. However, the age distribution of elements between the two genomes were vastly different (Figure 3). For instance, a much larger proportion of the *Anolis* genome was comprised of transposable element insertions ≤10% from their family consensus. This indicates an overabundance of inserts resulting from recent activity in *A. carolinensis* relative to *Sceloporus*. In particular, the *Anolis* genome contained far more recent SINEs (Kruskal Wallis test; p=9.374e-05). The distribution of recent LINEs was significantly different between the two genomes (p=2.824e-06), and *Anolis* contained more recent insertions from the L1 family (p=0.0001571), as well as RTE-BovB (p=0.001152) and R4 (p=0.0001571). The *Anolis* genome also contained more recent LTR retrotransposons (p=1.153-e07), as well as Mariner (p=0.0002122), Tigger (p=0.01017) and Chapaev (p=0.001152) DNA transposons.

Mitochondrial Genome Assembly

The mitochondrial genome was not captured by the genome sequencing approaches, likely due to how these types of libraries are prepared. However, mitochondrial sequence data obtained via RNAseq can be effectively assembled into whole mtDNA genomes [75–78]. We used RNAseq reads from 18 *S. undulatus* individuals from the RNAseq Dataset 4 (Table 2), which are from the same population as the individuals used for the genome sequencing. We used Trimmomatic v0.37 [52] to clean the raw reads and then mapped the clean reads to a complete *S. occidentalis* mtDNA genome [79] using BWA v0.7.15 [80]. Of the 632,987,330 total cleaned reads, 9.73% mapped to the *S. occidentalis* mtDNA genome with an average read depth of 5,164.42 reads per site per individual. After sorting and indexing mapped reads with SAMTOOLS v1.6 [81], we used the mpileup function in SAMTOOLS to build a consensus mitochondrial genome (mtGenome) excluding the reference and filling the no-coverage regions with “N” to generate 100% coverage of the mtGenome based on the consensus across the 18 individuals. We mapped the consensus genome to the well-annotated *Anolis carolinensis* mtGenome with MAFFT v1.3.7 [82] and transferred the annotation using the “copy annotation” command in GENEIOUS v.11.1.5 [83]. Annotations from the *A. carolinensis* mtGenome (17,223 bp) transferred well to the newly assembled *S. undulatus* mtGenome (17,072 bp), with 13 protein coding genes, 22 tRNA regions, 2 rRNA regions, and a control region (see full list in Supplemental File). While this genome is useful for understanding sequence variation and comparative genomics and phylogenetic analyses, this mitochondrial genome should not be used for examination of mitochondrial genome structure. The mitochondrial genome and the annotation are provided as supplemental data.

Addressing reference assembly quality using population-level transcriptomic and genomic data

In developing the high-quality reference genome for *S. undulatus*, we produced three assemblies using increasing amounts of data, for correspondingly greater costs. To assess the utility of each of the assemblies for addressing ecological genomic questions, we use two datasets: RNAseq and whole genome resequencing.

First, we used RNAseq Dataset 4 (Table 5) from n=18 males that were sampled from the same population (Alabama) as the individuals that were used to develop the reference assemblies; we then used these data to test whether the percentage of reads that mapped to the reference varied depending on which assembly we used as the reference. RNAseq data were cleaned with Trimmomatic v0.37 [52] and mapped with HISAT2 v2.1.0 [84] to each of the three *S. undulatus* genome assemblies. The percentage of reads that mapped were calculated using SAMTOOLS v1.6 flagstat [81]. We found negligible differences in mapping the RNAseq data to the SuperNova, HiRise and PBJelly assemblies where 81.49%, 82.37%, and 82.28% of cleaned reads mapped, respectively (Table 6).

Second, we prepared genomic DNA libraries for massively parallel sequencing for n=10 *S. undulatus* individuals (6 females, 4 males) from the same Alabama population as the individuals that were used to develop the reference assemblies. We also prepared libraries for n=5 *S. undulatus* individuals (1 female, 4 males) from Edgar Evins, Tennessee, and for n=5 individuals (2 females, 3 males) from St. Francis, Arkansas. This Arkansas population is at the boarders of the *S. undulatus* and *S. consobrinus* geographic distributions making its taxonomic status uncertain [18]. Specifically, we followed standard protocols for tissue DNA extraction from toe and/or tail clips with OMEGA EZNA Tissue spin-column kits. We then prepared sequencing libraries using the Illumina TruSeq Nano kit. We multiplexed these libraries with other individuals not included in this analysis and sequenced the library pool across two Illumina NovaSeq 6000 S4 sequencing runs. Five individuals from each of the three populations were sequenced to ~20x average read coverage; the remaining five individuals from Alabama were sequenced to lower coverage (~3x). Raw sequence read data were trimmed with Trimmomatic [52] and mapped separately to each of the three *S. undulatus* assemblies with bwa_mem [80] to each of the assemblies. SAMTOOLS flagstat [81] was used to calculate the total number of alignments in the .sam files generated during mapping and the number of shotgun reads that mapped to each assembly. The CollectWgsMetrics tool from the Picard Toolkit [85] was used to calculate genome-wide coverage of the mapped reads for each individual and assembly, and theoretical HET SNP sensitivity (a metric based on coverage and base-quality distribution that estimates probability of calling a true heterozygote SNP) as a way to predict the utility of each assembly as a reference for calling SNPs at high and low coverage. For all sequencing depths and populations, we observed fewer total alignments to the PBJelly Assembly than to either the HiRise or Supernova Assemblies (Table 6). Even though there were <0.5% fewer total reads that passed quality control (QC) with the PBJelly

Assembly/ SceUnd1.0, a higher percentage of the QC-passed reads mapped to this assembly than to either the HiRise or Supernova Assemblies (Table 6). We also determined that individuals from the same population as the *S. undulatus* individuals used to create these reference assemblies had a higher percentage of reads map to the assemblies than individuals from the Tennessee or Arkansas populations (Table 6). Those reads had lower whole-genome coverage and lower theoretical HET SNP sensitivity when mapped to the PBJelly/SceUnd1.0 Assembly than either the HiRise or Supernova Assemblies (Table 6). This may be due to repetitive regions being added to the assembly by the PacBio data, making it slightly less mappable. Both the RNAseq and the whole genome resequencing datasets support the conclusion that the 10X Chromium data that was used for the SuperNova Assembly covered the genome sufficiently to be a good reference for mapping RNAseq and WGS data, and that the HiC data (included in the HiRise Assembly) and the PacBio data (included in the final PBJelly Assembly) did not increase the amount of sequence information. Rather, the use of the HiC data and PacBio data resulted in larger scaffolds, which will aid in understanding the genomic context of expression data and sequence variants.

Assembly and refinement of genomic data for 34 additional Sceloporus species

Draft reduced-representation genomes are available for 34 species within *Sceloporus* [86,87] (phylogeny in Figure 4a). We downloaded the raw genomic reads for these 34 *Sceloporus* species from the Sequence Read Archive (Study Accession SRP041983; Table 7). Genomic resources for 33 of the species were obtained using reduced-representation libraries (yielding approximately 5 Gb per species), while one species, *S. occidentalis*, was sequenced using whole-genome shotgun sequencing (40.88 Gb; Table 7) [86]. To improve the draft assemblies for these 34 species, we mapped these raw reads to the final assembly, SceUnd1.0, using BWA-MEM [88]. Only the 11 longest, putative chromosome scaffolds from the SceUnd1.0 were used. The GATK version 3 [89–91] RealignerTargetCreator and IndelRealigner tools were used for local realignment, and HaplotypeCaller was used to identify insertion/deletion (INDEL) and single nucleotide polymorphism (SNP) variants. These sequence variants were separated and filtered with the SelectVariants and VariantFiltration tools using the GATK base settings. BEDTools [92] ‘genomecov’ tool was used to calculate coverage and identify regions with no coverage. We generated consensus sequences for each species by writing variants back over the reference fasta and replacing nucleotides with no coverage with “N”, using BCFtools [81] ‘consensus’ for SNPs and BEDTools ‘maskfasta’ for indels and regions with no mapping coverage (Supplemental Code File).

Mapping the reduced representation genome data from the 33 additional *Sceloporus* species improved the assemblies for each species. It seems there was a considerable amount of by-catch in many of the reduced-representation sequences that is normally filtered out when those reduced representation data are analyzed. For the species with ~5Gb of sequencing data, we improved the

genome coverage from an average of 1.23% to an average of 44.4% coverage at low depth (1-3X) (Figure S3). For *S. occidentalis* with ~ 41Gb of data, coverage improved from 61.0% to 88.7% (Table 7), at an average around 20X depth (Figure S3). Across the 33 species with ~ 5Gb of data, the BUSCO genes identified (complete and fragmented) in the reference-based assemblies ranged from 0.5 to 71.9% (complete and fragmented), whereas *S. occidentalis* had 95.9% BUSCO genes (complete and fragmented) identified, similar to our *S. undulatus* SuperNova Assembly (Table 7). Notably, across the *Sceloporus* genus, the percent of the raw data that mapped to the reference was negatively correlated with divergence time to the reference, *S. undulatus* ($p<0.0001$, $r=0.779$; Figure 4b). For species that are less then ~20 million years diverged from *S. undulatus*, >90% of reads mapped; the percentage of reads mapped declined to 75% when divergence was greater than 35 million years (Figure 4b).

It is important to note that the reference-based assemblies produced for these 34 species will correspond 1:1 with the synteny of the *S. undulatus* scaffolds. However, *Sceloporus* is notable among squamates for remarkable chromosome rearrangements with karyotypes ranging from 2N=22 to 2N=46 [31]. Therefore, the genome assemblies for species with karyotypes other than 2N=22 (the *S. undulatus* reference) or with large chromosomal inversions will not be reliable for addressing questions related to genomic architecture or structural variation [93]. However, these draft genomes contain a substantial amount of data that can be used for comparative genomic analyses. Figure S4 demonstrates the overlap in coverage of SceUnd1.0 by the reference-based genome assemblies. These distributions estimate that 50% of the genome would be covered by a subset of 16 species. Focusing on one gene of interest to our group, IGF1, we found that 16 of the 34 species had >75% coverage across the protein coding region of this gene and 24 of them had >50% coverage (Figure S4). Thereby, this dataset should prove useful for analyses of protein and gene sequence evolution to understand behavioral ecology, physiology, developmental biology, and more.

Analysis of synteny with other squamate chromosome-level genomes

As another benchmark of genome completeness, and to generate an initial look at chromosome evolution among squamates, we performed synteny analysis of the Eastern fence lizard (*S. undulatus*) SceUnd1.0 assembly with the green anole (*Anolis carolinensis*, AnoCar2.0) and with recently published chromosome-level assemblies for the Burmese python (*Python bivittatus*) [94] and the Argentine black and white tegu lizard (*Salvator merianae*) [43] (available at https://www.dnazoo.org/). The SceUnd1.0 scaffolds representing the 11 putative chromosomes were each divided into 1000 bp-long sequences that excluded gapped regions to serve as markers. Using BLAST, these markers were compared to the predicted chromosomes from the python and tegu HiC assemblies. BLAST hits for each were filtered to only include unique hits with greater than 80% identity, at least 500bp long, and part of 4 consecutive hits from the same Eastern fence

lizard chromosome, a method previously used for synteny analysis for the prairie rattlesnake [95]. Using these results, the Eastern fence lizard chromosomes were painted onto the anole, python, and tegu chromosomes to visualize large-scale synteny (Figure 5).

The decreased chromosome number in the *S. undulatus* species group compared to other *Sceloporus* lineages and the Iguanian group has long driven a hypothesis that a high number of fusions occurred in chromosomes in this species group, which is evident in the marker-based synteny painting of the *S. undulatus* genome. While the incomplete nature of the green anole genome, especially the lack of microchromosomes, makes many *Sceloporus* lineage-specific fusions difficult to identify, the inclusion of the tegu and python genomes provide guidance. For example, tegu macrochromosomes (ma) 6 and 7 and microchromosomes (mi) 2 and 5 are all syntenic to fence lizard ma6. However, the tegu macrochromosomes occur in a single block as the python X chromosome, and we cannot discern whether this was a fusion in a lineage preceding Iguanians and snakes or a fission in the tegu. The tegu microchromosomes are syntenic to python mi3 and mi9, which may have been fused in the fence lizard, considering the considerable size difference between fence lizard ma6 and the syntenic green anole ma6. Similarly, tegu mi1 and mi3 are syntenic to python mi3 and mi9, fusing to form the fence lizard mi3 but almost completely absent from the green anole assembly. These synteny results further support that the fourth largest microchromosome in the SceUnd1.0 assembly is syntenic to the anole X chromosome (Figure 3, Figure 5). However, it is not syntenic to the python X chromosome, which is syntenic to the Z chromosome in other snakes. The tegu sex chromosome has not been identified. Based on the blast hits from the anole X-linked genes and this synteny analysis we define the fourth largest microchromosome in the SceUnd1.0 assembly as the putative X chromosome, but functional data are needed to confirm this assignment.

Discussion

For the advancement of reptilian genomic and transcriptomic resources, we provide a high-quality, chromosome-level genome assembly for the Eastern fence lizard, *S. undulatus*, *de novo* transcriptomes for *S. undulatus* encompassing multiple tissues and life stages, and improved draft genome assemblies from 34 additional *Sceloporus* species. In the final reference assembly, SceUnd1.0, the largest 11 scaffolds contain 92.6% (1.765 of 1.905 Gb) of the genome sequence; these 11 scaffolds likely represent the 6 macro- and 5 microchromosomes of *S. undulatus*, based on karyotype, genome size, BUSCO analysis, and synteny with other squamate genomes. The remaining small scaffolds may contain some chromosome segments that could not be assembled, misassembled regions, or duplicated genes.

In comparing the three levels of reference genome assemblies, we found that the first level using only the 10X Genomics and the SuperNova Assembly contained all, or very nearly all, of the

protein-coding regions of the genome within its contigs (based on BUSCO and mapping of RNAseq and whole genome resequencing data). By including the Hi-C data, the contiguity of the HiRise Assembly dramatically improved, joining contigs into chromosome-length scaffolds, but had minimal effect on mapping percentages for either RNAseq or WGS. The inclusion of the PacBio data in the final PBJelly Assembly to produce SceUnd1.0 closed some gaps but yielded a relatively small improvement after the already dramatic improvements from the Hi-C data.

While it is now becoming possible to obtain a reference genome assembly for almost any organism, the quality and cost of reference genome assemblies vary considerably depending on the technologies used. This presents researchers with an important question: what levels of sequencing effort and assembly quality are required for a particular ecological genomics study? Important factors that must be considered include the sequencing depth, sequence contiguity, and thoroughness of annotation. Our study demonstrates that the SuperNova Assembly was sufficient for mapping RNAseq and whole-genome resequencing, while the more expensive data from HiC and PacBio were necessary to achieve high-level continuity and chromosome-level scaffolding in the HiRise and PBJ Assemblies.

Genome assemblies of high-quality and contiguity are critical for understanding organismal biology in a wide range of contexts that includes behavior, physiology, ecology, and evolution, on scales ranging from populations to higher-level clades. From RNAseq to ChIPseq (chromatin immunoprecipitation sequencing) and epigenetics, large-scale sequencing is rapidly becoming commonplace in ecological genomics to address fundamental questions of how organisms directly respond to their environment and how populations evolve in response to environmental variation. Many advanced molecular tools are typically reserved for traditional model organisms but with the large foundation of ecological and physiological data available for *S. undulatus*, a high-quality reference genome opens the door for these molecular techniques to be used in this ecological model organism. For example, with the recent demonstration of CRISPR-Cas9 gene modification in a lizard, the brown anole [96], a genome reference will facilitate the application of gene drive technologies for functional genomic studies in *Sceloporus* lizards. This reference will provide a foundation for whole genome studies to understand speciation and hybridization among closely related species utilizing low coverage re-sequencing, or as a point of comparison with more distantly related species relative to the chromosomal inversions and large-scale genome architectural changes common in the clade. *Sceloporus undulatus* and other lizards in the genus *Sceloporus* exhibit evolutionary reversals in sexual-size dimorphism and dichromatism and they have been used to demonstrate that androgens such as testosterone can inhibit growth in species (such as *S. undulatus*) in which females are the larger sex [19,97–99]. This SceUnd1.0 chromosome-level genome assembly would support ChIPseq or *in silico* analyses to identify sex hormone response elements. In addition, this assembly will facilitate the identification of

signatures of exposure to environmental stressors in both gene expression and epigenetic modification [100] to evaluate pressing questions on how climate change and invasive species affect local fauna. All of these uses for a chromosome-level genome assembly provide valuable extensions to ongoing work in the *Sceloporus* genus.

Availability of Supporting Data

Supplemental Data are hosted on the Auburn University Scholarly Repository, AUora: https://aurora.auburn.edu/handle/11200/49988

1. All three genome assemblies are provided as supplemental data.
    a. SuperNova assembly containing data from 10X Genomics Chromium: GenomeAssembly_SuperNova_Sceloporus_undulatus_pseudohap.fasta.gz
    b. HiRise assembly containing the 10X Genomics data with the addition of the Hi-C data: GenomeAssembly_HiRise_Sceloporus_undulatus.fasta.gz
    c. PBJelly Assembly (SceUnd1.0) containing the 10X Genomics data and the Hi-C data, with the addition of PacBio data: GenomeAssembly_SceUnd1.0_PBJELLY.fasta.gz
2. Tissue-Embryo Transcriptomes and annotation are provided as supplemental data.
    a. TranscriptomeAssemblyAnnotation.zip folder containing
        i. Transcriptome File: TranscriptomeAssembly_Tissues-Embryo_Trinity.fasta
        ii. Annotation File: TranscriptomeAssembly_Tissues-Embryo_Transdecoder.gff3
3. Truncated assembly used for the Funannotate annotation pipeline (SceUnd1.0_top24), and the annotation results are supplied as supplemental data.
    a. SceUnd1.0_top24.fasta. This file contains only the longest 24 scaffolds and they have been renamed 1-24 from longest to shortest.
    b. SceUnd1.0_top24_Annotation_FunnanotateResults.zip folder containing the following files:
        i. SceUnd1.0_top24.gff3
        ii. SceUnd1.0_top24.proteins.fa

iii. SceUnd1.0_top24.transcripts.fa
iv. SceUnd1.0_top24.annotations.txt
v. SceUnd1.0_top24_CompiledAnnotation.csv
vi. SceUnd1.0_top24.proteins.fa.report_EnsembleCombined.top.txt

4. The mitochondrial genomes and the annotation are provided as supplemental data.
   a. MitoGenomeAssembly_Sceloporus_undulatus.fasta
   b. MitoGenomeAssembly_Sceloporus_undulatus_Annotation.gff
5. The reference-based assemblies for the 34 *Sceloporus* species are provided as supplemental data.
   a. GenomeAssemblies_34Sceloporus.tar.gz
   b. Code for generating consensus sequences for each species: mkgenome_AW-AC.sh

Abbreviations

bp: Base pairs; **BUSCO**: Benchmarking Universal Single Copy Orthologues; **ChIPseq**: Chromatin immunoprecipitation sequencing; **E90N50**: N50 of the most highly expressed transcripts that represent 90% of the total normalized expression data; **Gb**: Gigabase pairs; **HET SNP**: Heterozygote single nucleotide polymorphism; **INDEL**: insertion/deletion; **Kb**: Kilobase pairs; **L50 and L90**: The smallest number of scaffolds that make up 50% or 90% of the total assembly length, respectively; **LINEs**: Long interspersed nuclear elements; **LTR transposons**: Long terminal repeat transposons; **ma**: Macrochromosome; **Mb**: Megabase pairs; **mi**: Microchromosome; **mtDNA**: Mitochondrial DNA; **N50 and N90**: The contig or scaffold length such that the sum of the lengths of all scaffolds of this size or larger is equal to 50% or 90%, respectively, of the total assembly length. **ORFs**: Open reading frames; **QC**: Quality control; **RIN**: RNA Integrity Number; **RNAseq**: RNA sequencing; **SceUnd1.0**: *Sceloporus undulatus* genome assembly including data from 10X Genomics Chromium library with Illumina sequencing, Hi-C library with Illumina sequencing, and PacBio sequencing assembled using the program PBJelly. Also referred to as the PBJelly assembly; **SceUnd1.0_top24**: *Sceloporus undulatus* genome assembly including only the longest 24 scaffolds from SceUnd1.0; **SINEs**: Short interspersed nuclear elements; **SNP**: Single Nucleotide Polymorphism; **tRNA**: Transfer RNA

Competing Interests

None Declared

Funding

This work was supported by NSF GRFP (DGE 1414475 to AC; DGE 1255832 to APS); NSF BCS-1554834 to GHP; NSF-IOS-PMB 1855845 to ADL; NSF-IOS-1456655 to TL; Clemson University lab funds to MS; Georgia Southern Startup Funds to CLC; University of Virginia start-up funding to RMC; Hatch Multistate W3045 project no. NJ17240 to HJA; Grant for Postdoctoral Interdisciplinary Research in the Life Sciences from the School of Life Sciences at Arizona State University to MT; Auburn University Start-up Funds to TSS.

Acknowledgements

We are grateful for the support of the DoveTail Genomics and Auburn University Office of Information Technology and Hopper High-Performance Computing Cluster for assistance with this work. We thank Kirsty MacLeod for catching the adult male used for sequencing, and Juan Rodriguez for bioinformatic assistance.

Authors' Contributions

AW: Data curation; Formal analysis; Investigation; Validation; Visualization; Writing – original; Writing – review & editing

RST: Conceptualization; Data curation; Formal analysis; Investigation; Validation; Visualization; Writing – review & editing

MBG: Data curation; Formal analysis; Investigation; Validation; Visualization; Writing – original; Writing – review & editing

DSW: Data curation; Formal analysis; Software; Validation; Visualization; Writing – original; Writing – review & editing

AC: Data curation; Formal analysis; Methodology; Software, Validation, Visualization

DYS: Formal analysis; Software; Writing – original; Writing – review & editing

RK: Methodology; Formal analysis; Writing- original draft; Writing- review & editing

AC: Data curation; Formal analysis; Methodology; Software, Validation, Visualization

APS: Formal analysis; Writing – original draft; Writing – review & editing

CLC: Conceptualization; Data Curation; Investigation; Funding Acquisition; Writing-review & editing

GP: Funding acquisition; Supervision, Writing – review & editing.

MT: Data curation; Formal analysis; Methodology; Funding acquisition; Writing – review & editing

TL: Conceptualization; Funding acquisition; Resources; Writing – review & editing

KK: Conceptualization; Funding acquisition; Resources; Writing – review & editing

MWS: Resources; Funding Acquisition; Writing- review & editing

ADL: Conceptualization; Data curation; Funding acquisition; Methodology; Writing – original; Writing – review & editing

MJA: Conceptualization; Funding acquisition; Writing – review & editing

MEG: Conceptualization; Writing – review & editing

HJA: Investigation; Funding acquisition; Writing – review & editing

RMC: Conceptualization; Funding acquisition; Investigation; Writing – review & editing

TSS: Conceptualization; Data curation; Funding acquisition; Formal analysis; Investigation; Project Administration; Resources; Supervision; Writing – original; Writing – review & editing.

All authors have read and approved the final version of the manuscript.

References

1. Seebacher F. A review of thermoregulation and physiological performance in reptiles: what is the role of phenotypic flexibility? *J Comp Physiol B*. 2005; doi: 10.1007/s00360-005-0010-6.

2. Kearney M, Fujita MK, Ridenour J. Lost sex in the reptiles: constraints and correlations. In: Schön I, Martens K, Dijk P, editors. *Lost Sex: The Evolutionary Biology of Parthenogenesis*. Dordrecht: Springer Netherlands;

3. Van Dyke JU, Brandley MC, Thompson MB. The evolution of viviparity: molecular and genomic data from squamate reptiles advance understanding of live birth in amniotes. *Reproduction*. 2014; doi: 10.1530/REP-13-0309.

4. Rhen T, Schroeder A. Molecular Mechanisms of Sex Determination in Reptiles. *SXD*. Karger Publishers; 2010; doi: 10.1159/000282495.

5. Sarre SD, Ezaz T, Georges A. Transitions between sex-determining systems in reptiles and amphibians. *Annu Rev Genom Hum Genet*. Annual Reviews; 2011; doi: 10.1146/annurev-genom-082410-101518.

6. Bergmann PJ, Morinaga G. The convergent evolution of snake-like forms by divergent evolutionary pathways in squamate reptiles. *Evolution*. 2019; doi: https://doi.org/10.1111/evo.13651.

7. Liu Y, Zhou Q, Wang Y, Luo L, Yang J, Yang L, et al.. *Gekko japonicus* genome reveals evolution of adhesive toe pads and tail regeneration. *Nature Communications*. Nature Publishing Group; 2015; doi: 10.1038/ncomms10033.

8. Andrew AL, Perry BW, Card DC, Schield DR, Ruggiero RP, McGaugh SE, et al.. Growth and stress response mechanisms underlying post-feeding regenerative organ growth in the Burmese python. *BMC Genomics*. 2017; doi: 10.1186/s12864-017-3743-1.

9. Janes DE, Organ CL, Fujita MK, Shedlock AM, Edwards SV. Genome evolution in Reptilia, the sister group of mammals. *Annual Review of Genomics and Human Genetics*. 2010; doi: 10.1146/annurev-genom-082509-141646.

10. Alföldi J, Palma FD, Grabherr M, Williams C, Kong L, Mauceli E, et al.. The genome of the green anole lizard and a comparative analysis with birds and mammals. *Nature*. 2011; doi: 10.1038/nature10390.

11. Georges A, Li Q, Lian J, O'Meally D, Deakin J, Wang Z, et al.. High-coverage sequencing and annotated assembly of the genome of the Australian dragon lizard Pogona vitticeps. *GigaScience*. 2015; doi: 10.1186/s13742-015-0085-2.

12. Xiong Z, Li F, Li Q, Zhou L, Gamble T, Zheng J, et al.. Draft genome of the leopard gecko, *Eublepharis macularius*. *Gigascience*. Oxford Academic; 2016; doi: 10.1186/s13742-016-0151-4.

13. Lind AL, Lai YYY, Mostovoy Y, Holloway AK, Iannucci A, Mak ACY, et al.. Genome of the Komodo dragon reveals adaptations in the cardiovascular and chemosensory systems of monitor lizards. *Nature Ecology & Evolution*. Nature Publishing Group; 2019; doi: 10.1038/s41559-019-0945-8.

14. Olmo E. Trends in the evolution of reptilian chromosomes. *Integrative and Comparative Biology*. 2008; doi: 10.1093/icb/icn049.

15. Hedges SB, Marin J, Suleski M, Paymer M, Kumar S. Tree of life reveals clock-Like speciation and diversification. *Mol Biol Evol*. Oxford Academic; 2015; doi: 10.1093/molbev/msv037.

16. Zhang G, Li C, Li Q, Li B, Larkin DM, Lee C, et al.. Comparative genomics reveals insights into avian genome evolution and adaptation. *Science*. 2014; doi: 10.1126/science.1251385.

17. Pasquesi GIM, Adams RH, Card DC, Schield DR, Corbin AB, Perry BW, et al.. Squamate reptiles challenge paradigms of genomic repeat element evolution set by birds and

mammals. *Nature Communications*. Nature Publishing Group; 2018; doi: 10.1038/s41467-018-05279-1.

18. Leaché AD. Species tree discordance traces to phylogeographic clade boundaries in North American fence lizards (*Sceloporus*). *Syst Biol*. Oxford Academic; 2009; doi: 10.1093/sysbio/syp057.

19. John-Alder HB, Cox RM, Haenel GJ, Smith LC. Hormones, performance and fitness: Natural history and endocrine experiments on a lizard (*Sceloporus undulatus*). *Integrative and Comparative Biology*. 2009; doi: 10.1093/icb/icp060.

20. Buckley LB, Urban MC, Angilletta MJ, Crozier LG, Rissler LJ, Sears MW. Can mechanism inform species' distribution models? *Ecology Letters*. 2010; doi: https://doi.org/10.1111/j.1461-0248.2010.01479.x.

21. Warner DA, Andrews RM. Nest-site selection in relation to temperature and moisture by the lizard, *Sceloporus undulatus*. *herp*. The Herpetologists' League; 2002; doi: 10.1655/0018-0831(2002)058[0399:NSIRTT]2.0.CO;2.

22. Telemeco RS, Fletcher B, Levy O, Riley A, Rodriguez-Sanchez Y, Smith C, et al.. Lizards fail to plastically adjust nesting behavior or thermal tolerance as needed to buffer populations from climate warming. *Global Change Biology*. 2017; doi: https://doi.org/10.1111/gcb.13476.

23. Blackburn DG, Gavelis GS, Anderson KE, Johnson AR, Dunlap KD. Placental specializations of the mountain spiny lizard *Sceloporus jarrovi*. *Journal of Morphology*. 2010; doi: https://doi.org/10.1002/jmor.10860.

24. Anderson KE, Blackburn DG, Dunlap KD. Scanning electron microscopy of the placental interface in the viviparous lizard *Sceloporus jarrovi* (Squamata: Phrynosomatidae). *Journal of Morphology*. John Wiley & Sons, Ltd; 2011; doi: 10.1002/jmor.10925.

25. Lambert SM, Wiens JJ. Evolution of viviparity: A phylogenetic test of the cold-climate hypothesis in *Phrynosomatid* lizards. *Evolution*. 2013; doi: https://doi.org/10.1111/evo.12130.

26. Angilletta MJ, Niewiarowski PH, Dunham AE, Leaché AD, Porter WP. Bergmann's Clines in ectotherms: Illustrating a life-history perspective with sceloporine lizards. *Am Nat*. 2004; doi: 10.1086/425222.

27. Angilletta MJ, Oufiero CE, Leaché AD. Direct and indirect effects of environmental temperature on the evolution of reproductive strategies: an information-theoretic approach. *Am Nat*. 2006; doi: 10.1086/507880.

28. Tinkle DW, Ballinger RE. *Sceloporus undulatus*: A Study of the intraspecific comparative demography of a lizard. *Ecology*. 1972; doi: https://doi.org/10.2307/1934772.

29. Lawing AM, Polly PD, Hews DK, Martins EP. Including Fossils in Phylogenetic Climate Reconstructions: A Deep Time Perspective on the Climatic Niche Evolution and

Diversification of Spiny Lizards (*Sceloporus*). *The American Naturalist*. 2016; doi: 10.1086/687202.

30. Rosenblum EB, Parent CE, Diepeveen ET, Noss C, Bi K. Convergent phenotypic evolution despite contrasting demographic histories in the fauna of white sands. *The American Naturalist*. The University of Chicago Press; 2017; doi: 10.1086/692138.

31. Leaché AD, Sites JW. Chromosome evolution and diversification in North American spiny lizards (genus *Sceloporus*). *Cytogenet Genome Res*. 2009; doi: 10.1159/000293285.

32. Leaché AD, Reeder TW. Molecular systematics of the Eastern Fence Lizard (*Sceloporus undulatus*): A comparison of parsimony, likelihood, and bayesian approaches. *Syst Biol*. Oxford Academic; 2002; doi: 10.1080/10635150275347 5871.

33. Cox RM, Butler MA, John-Alder HB. The evolution of sexual size dimorphism in reptiles. Sex, Size and Gender Roles. Oxford University Press

34. Pollock NB, Feigin S, Drazenovic M, John-Alder HB. Sex hormones and the development of sexual size dimorphism: 5α-dihydrotestosterone inhibits growth in a female-larger lizard (*Sceloporus undulatus*). *J Exp Biol*. 2017; doi: 10.1242/jeb.166553.

35. Trompeter WP, Langkilde T. Invader danger: Lizards faced with novel predators exhibit an altered behavioral response to stress. *Hormones and Behavior*. 2011; doi: 10.1016/j.yhbeh.2011.04.001.

36. Graham SP, Freidenfelds NA, Thawley CJ, Robbins TR, Langkilde T. Are invasive species stressful? The gucocorticoid profile of native lizards exposed to invasive fire ants depends on the context. *Physiological and Biochemical Zoology*. The University of Chicago Press; 2016; doi: 10.1086/689983.

37. Gifford ME, Robinson CD, Clay TA. The influence of invasive fire ants on survival, space use, and patterns of natural selection in juvenile lizards. *Biol Invasions*. 2017; doi: 10.1007/s10530-017-1370-z.

38. Angilletta MJ Jr, Zelic MH, Adrian GJ, Hurliman AM, Smith CD. Heat tolerance during embryonic development has not diverged among populations of a widespread species (*Sceloporus undulatus*). *Conservation Physiology*. 2013; doi: 10.1093/conphys/cot018.

39. Buckley LB, Ehrenberger JC, Angilletta MJ. Thermoregulatory behaviour limits local adaptation of thermal niches and confers sensitivity to climate change. *Functional Ecology*. 2015; doi: https://doi.org/10.1111/1365-2435.12406.

40. Carlo MA, Riddell EA, Levy O, Sears MW. Recurrent sublethal warming reduces embryonic survival, inhibits juvenile growth, and alters species distribution projections under climate change. *Ecol Lett*. 2018; doi: 10.1111/ele.12877.

41. Zheng GXY, Lau BT, Schnall-Levin M, Jarosz M, Bell JM, Hindson CM, et al.. Haplotyping germline and cancer genomes with high-throughput linked-read sequencing. *Nature Biotechnology*. Nature Publishing Group; 2016; doi: 10.1038/nbt.3432.

42. English AC, Richards S, Han Y, Wang M, Vee V, Qu J, et al.. Mind the Gap: Upgrading Genomes with Pacific Biosciences RS Long-Read Sequencing Technology. *PLOS ONE*. Public Library of Science; 2012; doi: 10.1371/journal.pone.0047768.

43. Roscito JG, Sameith K, Pippel M, Francoijs K-J, Winkler S, Dahl A, et al.. The genome of the tegu lizard Salvator merianae: combining Illumina, PacBio, and optical mapping data to generate a highly contiguous assembly. *Gigascience*. 2018; doi: 10.1093/gigascience/giy141.

44. Cole CJ. Chromosome variation in North American fence lizards (Genus *Sceloporus; undulatus* species group). *Syst Biol*. Oxford Academic; 1972; doi: 10.1093/sysbio/21.4.357.

45. Laetsch DR, Blaxter ML. BlobTools: Interrogation of genome assemblies. *F1000Res*. 2017; doi: 10.12688/f1000research.12232.1.

46. Simão FA, Waterhouse RM, Ioannidis P, Kriventseva EV, Zdobnov EM. BUSCO: assessing genome assembly and annotation completeness with single-copy orthologs. *Bioinformatics*. 2015; doi: 10.1093/bioinformatics/btv351.

47. Waterhouse RM, Seppey M, Simão FA, Manni M, Ioannidis P, Klioutchnikov G, et al.. BUSCO applications from quality assessments to gene prediction and phylogenomics. *Mol Biol Evol*. Oxford Academic; 2018; doi: 10.1093/molbev/msx319.

48. Fisher RE, Geiger LA, Stroik LK, Hutchins ED, George RM, Denardo DF, et al.. A histological comparison of the original and regenerated tail in the Green Anole, *Anolis carolinensis*. *The Anatomical Record*. 2012; doi: https://doi.org/10.1002/ar.22537.

49. Ritzman TB, Stroik LK, Julik E, Hutchins ED, Lasku E, Denardo DF, et al.. The gross anatomy of the original and regenerated tail in the Green Anole (*Anolis carolinensis*). *The Anatomical Record*. 2012; doi: https://doi.org/10.1002/ar.22524.

50. McGaugh SE, Bronikowski AM, Kuo C-H, Reding DM, Addis EA, Flagel LE, et al.. Rapid molecular evolution across amniotes of the IIS/TOR network. *Proceedings of the National Academy of Sciences*. 2015; doi: 10.1073/pnas.1419659112.

51. McGaugh SE, Bronikowski AM, Kuo C-H, Reding DM, Addis EA, Flagel LE, et al.. Data from: Rapid molecular evolution across amniotes of the IIS/TOR network. Dryad; DOI: 10.5061/DRYAD.VN872

52. Bolger AM, Lohse M, Usadel B. Trimmomatic: a flexible trimmer for Illumina sequence data. *Bioinformatics*. 2014; doi: 10.1093/bioinformatics/btu170.

53. Grabherr MG, Haas BJ, Yassour M, Levin JZ, Thompson DA, Amit I, et al.. Full-length transcriptome assembly from RNA-Seq data without a reference genome. *Nature Biotechnology*. Nature Publishing Group; 2011; doi: 10.1038/nbt.1883.

54. Wu CH, Apweiler R, Bairoch A, Natale DA, Barker WC, Boeckmann B, et al.. The universal protein resource (UniProt): an expanding universe of protein information. *Nucleic Acids Res*. Oxford Academic; 2006; doi: 10.1093/nar/gkj161.

55. Finn RD, Coggill P, Eberhardt RY, Eddy SR, Mistry J, Mitchell AL, et al.. The Pfam protein families database: towards a more sustainable future. *Nucleic Acids Res*. 2016; doi: 10.1093/nar/gkv1344.

56. Langmead B, Salzberg SL. Fast gapped-read alignment with Bowtie 2. *Nature Methods*. Nature Publishing Group; 2012; doi: 10.1038/nmeth.1923.

57. Camacho C, Coulouris G, Avagyan V, Ma N, Papadopoulos J, Bealer K, et al.. BLAST+: architecture and applications. *BMC Bioinformatics*. 2009; doi: 10.1186/1471-2105-10-421.

58. Eddy SR. A new generation of homology search tools based on probabilistic inference. *Genome Inform*. 23:205–112009;

59. Eckalbar WL, Hutchins ED, Markov GJ, Allen AN, Corneveaux JJ, Lindblad-Toh K, et al.. Genome reannotation of the lizard *Anolis carolinensis* based on 14 adult and embryonic deep transcriptomes. *BMC Genomics*. 2013; doi: 10.1186/1471-2164-14-49.

60. Stanke M, Schöffmann O, Morgenstern B, Waack S. Gene prediction in eukaryotes with a generalized hidden Markov model that uses hints from external sources. *BMC Bioinformatics*. 2006; doi: 10.1186/1471-2105-7-62.

61. Lomsadze A, Burns PD, Borodovsky M. Integration of mapped RNA-Seq reads into automatic training of eukaryotic gene finding algorithm. *Nucleic Acids Res*. Oxford Academic; 2014; doi: 10.1093/nar/gku557.

62. Lowe TM, Chan PP. tRNAscan-SE On-line: integrating search and context for analysis of transfer RNA genes. *Nucleic Acids Res*. Oxford Academic; 2016; doi: 10.1093/nar/gkw413.

63. Jones P, Binns D, Chang H-Y, Fraser M, Li W, McAnulla C, et al.. InterProScan 5: genome-scale protein function classification. *Bioinformatics*. 2014; doi: 10.1093/bioinformatics/btu031.

64. Huerta-Cepas J, Szklarczyk D, Forslund K, Cook H, Heller D, Walter MC, et al.. eggNOG 4.5: a hierarchical orthology framework with improved functional annotations for eukaryotic, prokaryotic and viral sequences. *Nucleic Acids Res*. 2016; doi: 10.1093/nar/gkv1248.

65. Rawlings ND, Waller M, Barrett AJ, Bateman A. MEROPS: the database of proteolytic enzymes, their substrates and inhibitors. *Nucleic Acids Res*. 2014; doi: 10.1093/nar/gkt953.

66. Buchfink B, Xie C, Huson DH. Fast and sensitive protein alignment using DIAMOND. *Nature Methods*. 2015; doi: 10.1038/nmeth.3176.

67. Sites JW, Archie JW, Cole CJ, Flores-Villela O. A review of phylogenetic hypotheses for lizards of the genus *Sceloporus* (Phrynosomatidae): implications for ecological and evolutionary studies. Bulletin of the AMNH ; no. 213. *Sceloporus phylogeny*. [New York] : American Museum of Natural History; 1992;

68. Lisachov AP, Tishakova KV, Romanenko SA, Molodtseva AS, Prokopov DYu, Pereira JC, et al.. Whole-chromosome fusions in the karyotype evolution of *Sceloporus* (Iguania, Reptilia) are more intense in sex chromosomes than autosomes. Genomics; 2020 Mar.

69. Rovatsos M, Altmanová M, Pokorná M, Kratochvíl L. Conserved sex chromosomes across adaptively radiated *Anolis* Lizards. *Evolution*. 2014; doi: https://doi.org/10.1111/evo.12357.

70. Rovatsos M, Altmanová M, Pokorná MJ, Kratochvíl L. Novel X-linked genes revealed by quantitative polymerase chain reaction in the green anole, *Anolis carolinensis*. *G3 (Bethesda)*. 2014; doi: 10.1534/g3.114.014084.

71. Smit A, Hubley R, Green P. RepeatModeler Open-1.0. Institute for Systems Biology;

72. Smit A, Hubley R, Green P. RepeatMasker Open-4.0. Institute for Systems Biology;

73. Jurka J, Kapitonov VV, Pavlicek A, Klonowski P, Kohany O, Walichiewicz J. Repbase Update, a database of eukaryotic repetitive elements. *CGR*. Karger Publishers; 2005; doi: 10.1159/000084979.

74. Tollis M, Boissinot S. The transposable element profile of the anolis genome. *Mob Genet Elements*. 2011; doi: 10.4161/mge.1.2.17733.

75. Smith DR. RNA-Seq data: a goldmine for organelle research. *Brief Funct Genomics*. Oxford Academic; 2013; doi: 10.1093/bfgp/els066.

76. Schwartz TS, Arendsee ZW, Bronikowski AM. Mitochondrial divergence between slow- and fast-aging garter snakes. *Experimental Gerontology*. 2015; doi: 10.1016/j.exger.2015.09.004.

77. Tian Y, Smith DR. Recovering complete mitochondrial genome sequences from RNA-Seq: A case study of Polytomella non-photosynthetic green algae. *Molecular Phylogenetics and Evolution*. 2016; doi: 10.1016/j.ympev.2016.01.017.

78. Waits DS, Simpson DY, Sparkman AM, Bronikowski AM, Schwartz TS. The utility of reptile blood transcriptomes in molecular ecology. *Mol Ecol Resour*. 2020; doi: 10.1111/1755-0998.13110.

79. Kumazawa Y. Mitochondrial DNA sequences of five squamates: phylogenetic affiliation of snakes. *DNA Res*. Oxford Academic; 2004; doi: 10.1093/dnares/11.2.137.

80. Li H, Durbin R. Fast and accurate short read alignment with Burrows–Wheeler transform. *Bioinformatics*. Oxford Academic; 2009; doi: 10.1093/bioinformatics/btp324.

81. Li H, Handsaker B, Wysoker A, Fennell T, Ruan J, Homer N, et al.. The Sequence Alignment/Map format and SAMtools. *Bioinformatics*. 2009; doi: 10.1093/bioinformatics/btp352.

82. Katoh K, Standley DM. MAFFT Multiple Sequence Alignment Software Version 7: Improvements in Performance and Usability. *Mol Biol Evol*. 2013; doi: 10.1093/molbev/mst010.

83. Kearse M, Moir R, Wilson A, Stones-Havas S, Cheung M, Sturrock S, et al.. Geneious Basic: An integrated and extendable desktop software platform for the organization and analysis of sequence data. *Bioinformatics*. 2012; doi: 10.1093/bioinformatics/bts199.

84. Pertea M, Kim D, Pertea GM, Leek JT, Salzberg SL. Transcript-level expression analysis of RNA-seq experiments with HISAT, StringTie and Ballgown. *Nature Protocols*. Nature Publishing Group; 2016; doi: 10.1038/nprot.2016.095.

85. Broad Institute: Picard Tools - By Broad Institute. http://broadinstitute.github.io/picard/ Accessed 2020 Dec 15.

86. Leaché AD, Harris RB, Maliska ME, Linkem CW. Comparative species divergence across eight triplets of Spiny Lizards (*Sceloporus*) using genomic sequence data. *Genome Biology and Evolution*. 2013; doi: 10.1093/gbe/evt186.

87. Arthofer W, Banbury BL, Carneiro M, Cicconardi F, Duda TF, Harris RB, et al.. Genomic Resources Notes Accepted 1 August 2014–30 September 2014. *Molecular Ecology Resources*. 2015; doi: https://doi.org/10.1111/1755-0998.12340.

88. Li H. Aligning sequence reads, clone sequences and assembly contigs with BWA-MEM. *arXiv:13033997 [q-bio]*. 2013;

89. McKenna A, Hanna M, Banks E, Sivachenko A, Cibulskis K, Kernytsky A, et al.. The Genome Analysis Toolkit: a MapReduce framework for analyzing next-generation DNA sequencing data. *Genome Res*. 2010; doi: 10.1101/gr.107524.110.

90. DePristo MA, Banks E, Poplin R, Garimella KV, Maguire JR, Hartl C, et al.. A framework for variation discovery and genotyping using next-generation DNA sequencing data. *Nature Genetics*. Nature Publishing Group; 2011; doi: 10.1038/ng.806.

91. Auwera GAV der, Carneiro MO, Hartl C, Poplin R, Angel G del, Levy-Moonshine A, et al.. From FastQ data to high-confidence variant calls: The Genome Analysis Toolkit best practices pipeline. *Current Protocols in Bioinformatics*. 2013; doi: https://doi.org/10.1002/0471250953.bi1110s43.

92. Quinlan AR, Hall IM. BEDTools: a flexible suite of utilities for comparing genomic features. *Bioinformatics*. Oxford Academic; 2010; doi: 10.1093/bioinformatics/btq033.

93. Bedoya AM, Leaché AD. Characterization of a large pericentric inversion in plateau fence lizards (*Sceloporus tristichus*): evidence from chromosome-scale genomes | bioRxiv. *bioRxiv*. 2020;

94. Castoe TA, de Koning APJ, Hall KT, Card DC, Schield DR, Fujita MK, et al.. The Burmese python genome reveals the molecular basis for extreme adaptation in snakes. *Proc Natl Acad Sci USA*. 2013; doi: 10.1073/pnas.1314475110.

95. Schield DR, Card DC, Hales NR, Perry BW, Pasquesi GM, Blackmon H, et al.. The origins and evolution of chromosomes, dosage compensation, and mechanisms underlying venom regulation in snakes. *Genome Res*. 2019; doi: 10.1101/gr.240952.118.

96. Rasys AM, Park S, Ball RE, Alcala AJ, Lauderdale JD, Menke DB. CRISPR-Cas9 gene editing in lizards through microinjection of unfertilized oocytes. *Cell Reports*. 2019; doi: 10.1016/j.celrep.2019.07.089.

97. Cox RM, Skelly SL, John-Alder HB. Testosterone inhibits growth in juvenile male eastern fence lizards (*Sceloporus undulatus*): implications for energy allocation and sexual size dimorphism. *Physiol Biochem Zool*. 2005; doi: 10.1086/430226.

98. Cox RM, John-Alder HB. Testosterone has opposite effects on male growth in lizards (*Sceloporus* spp.) with opposite patterns of sexual size dimorphism. *J Exp Biol*. 2005; doi: 10.1242/jeb.01948.

99. John-Alder HB, Cox RM, Taylor EN. Proximate developmental mediators of sexual dimorphism in size: case studies from squamate reptiles. *Integr Comp Biol*. Oxford Academic; 2007; doi: 10.1093/icb/icm010.

100. Schrey AW, Robbins TR, Lee J, Dukes DW, Ragsdale AK, Thawley CJ, et al.. Epigenetic response to environmental change: DNA methylation varies with invasion status. *Environ Epigenet*. 2016; doi: 10.1093/eep/dvw008.

Table Click here to access/download;Table;Tables_2021-04-16.docx

Table 1: Summary statistics across genome assemblies for *Sceloporus undulatus*.

**Metric**	**Supernova Assembly (10X Chromium)**	**HiRise Assembly (10X Chromium + Hi-C)**	**PBJelly Assembly (SceUnd1.0) (10X Chromium + Hi-C + PacBio)**
**Coverage**	46X	4859X	4859X
**Contig N50**	0.049 Mb	0.073 Mb	0.193 Mb
**Scaffold N50**	2.55 Mb	265.4 Mb	275.6 Mb
**Scaffold N90**	0.241Mb	35.4 Mb	37.1 Mb
**Scaffold L50**	218 scaffold	3 scaffolds	3 scaffolds
**Scaffold L90**	987 scaffolds	9 scaffolds	9 scaffolds
**Tetrapoda BUSCO (n=3950) on whole genome**	89.5% Complete, 6.4% Fragmented 4.1% Missing	90.2% Complete 5.5% Fragmented 4.3% Missing	90.9% Complete, 5.0% Fragmented 4.1% Missing
**Tetrapoda BUSCO (n=3950) on top 24 scaffolds**			90.7% Complete, 4.9% Fragmented 4.4% Missing
**Tetrapoda BUSCO (n=3950) on predicted proteins from top 24 scaffolds**			79.1% Complete 13.7% Fragmented 7.2% Missing
**Assembly Size**	1.61 Gb	1.836 Gb	1.9056 GB with gaps 1.8586 GB without gaps Annotation: 21,050 of our predicted proteins had hits in ENSEMBL.

N50 - The contig or scaffold length such that the sum of the lengths of all scaffolds of this size or larger is equal to 50% of the total assembly length.
N90 - The scaffold length such that the sum of the lengths of all scaffolds of this size or larger is equal to 90% of the total assembly length.
L50 - The smallest number of scaffolds that make up 50% of the total assembly length.

L90 - The smallest number of scaffolds that make up 90% of the total assembly length.

Table 2: *Sceloporus undulatus de novo* transcriptome assembly statistics. The 4 tissues are comprised of 3 tissues first reported in this study (brain, skeletal, and embryos) from gravid females collected in Edgefield County, SC, plus liver tissue previously reported by McGaugh et al. 2015 [51].

**Assembly**	**1 tissue [51]**	**3 tissues**	**4 tissues**
Total of Trinity transcripts	158,323	492,249	547,370
Total of Trinity 'genes'	138,031	422,687	467,658
GC%	43.81	42.85	42.76
Contig N50	1,720	1,648	1,438
Contig E90N50	2,254	2,640	2,550
Average contig length (bp)	833.0	822.4	781.5
Transcripts with the longest ORFs	86,630 (54.7%)	212,172 (43.1%)	217,756 (39.8%)

Table 3: BUSCO results for transcriptomes of two lizard species. For *Sceloporus undulatus*, the 4 tissues are the 3 tissues (brain, skeletal muscle and embryos) first reported here with the addition of 1 tissue (liver) from McGaugh et al. 2015 [51]. For *Anolis carolinensis*, see Eckalbar et al. 2013 [59] for the complete list of tissues used.

	Sceloporus undulatus			***Anolis carolinensis***
	**1 tissue**	**3 tissues**	**4 tissues**	**14 tissues**
Complete genes	72.5%	91.7%	92.3%	96.7%
Duplicated genes	25%	43.8%	43.9%	37.9%
Fragmented genes	9.2%	4.8%	4.8%	1.1%
Missing genes	18.3%	3.5%	2.9%	2.2%
Reference	McGaugh et al. 2015	This study	This study	Eckalbar et al, 2013[59]

Table 4. Annotation of *Sceloporus undulatus de novo* transcriptome assembly using 4 tissues. Unique annotation numbers between parentheses.

**Annotation**	
Annotated genes	467,658
Annotated transcript isoforms	547,370
Annotated isoforms/genes	1.17
Transcripts with Swiss-Prot annotation	(71,944)
Transcripts with PFAM annotation	51,018 (46,432)
Transcripts with KEGG annotation	65,694 (21,520)
Transcripts with GO annotation	73,936 (66,554)

Table 5. RNAseq datasets used for training the genome annotation pipeline. Datasets 1 and 2 were also used in the *de novo* transcriptome assembly.

Data Set	Tissue	Age	Sex	Treatment/ Condition	Data Type	NCBI SRA Accession #
**1. This Paper**	Skeletal muscle	Adult	Female	Post-reproductive	100 bp PE	SAMN06312743
	Brain	Adult	Female	Post-reproductive	100 bp PE	SAMN06312741
	Whole Embryo	Embryo	N/A		100 bp PE	SAMN06312742
**2. McGaugh et al. 2015**	Liver	Juvenile		Control Lab	100 bp PE	SRR629640
**3. Cox et al. In Review**	Liver	Juvenile	Female	Blank	125 bp PE	SAMN14774299
	Liver	Juvenile	Male	Castrated	125 bp PE	through
	Liver	Juvenile	Male	Control	125 bp PE	SAMN14774321
	Liver	Juvenile	Female	Testosterone	125 bp PE	
	Liver	Juvenile	Male	Testosterone	125 bp PE	
**4. Simpson et al. In Prep.**	Liver	Adult	Male	Control Lab	150 bp PE	SAMN08687228
	Liver	Adult	Male	Acute Heat Stress	150 bp PE	through
	Liver	Adult	Male	Fire Ant Bitten	150 bp PE	SAMN08687245

McGaugh SE, Bronikowski AM, Kuo C-H, Reding DM, Addis EA, Flagel LE, et al. Data from: Rapid molecular evolution across amniotes of the IIS/TOR network. Dryad Digital Repository. http://dx.doi.org/10.5061/dryad.vn872. 2015.

Cox, C. L., A. K. Chung, D. C. Card, T. A. Castoe, N. Pollock, H. John-Alder, and R. M. Cox. Evolutionary regulation of sex-biased gene expression and sexual dimorphism.

Simpson, D., R. Telemeco, T. Langkilde, T. S. Schwartz. Different ecological stressors have contrasting transcriptomic responses.

Table 6. Comparison of each genome assembly type as a reference for population-level analyses for RNAseq and Whole Genome Sequencing of *Sceloporus undulatus* individuals from Alabama (AL, either low or high coverage), Tennessee (TN) and Arkansas (AR). Datasets were mapped to either the SuperNova Assembly containing only the 10X Genomics Chromium data, the HiRise Assembly containing 10X Genomics Chromium and Hi-C data, or the PBJelly assembly (SceUnd1.0) containing 10X Genomics Chromium, Hi-C, and PacBio data. Average SAMTOOLS QC-passed reads, reads mapped, and percentage of mapped QC-passed reads for every sequencing depth and population are shown along with average whole-genome coverage and theoretical HET SNP sensitivity for every assembly and population.

		RNAseq-AL	Low Cov-AL	High Cov-AL	High Cov-TN	High Cov-AR
**SuperNova**	**QC-passed Reads**	3.28E7 ± 6.83E6	5.11E7 ± 3.36E7	3.33E8 ± 2.66E7	3.47E8 ± 9.39E7	3.33E8 ± 6.14E7
	**Reads Mapped**	2.68E7 ± 6.19E6	5.07E7 ± 3.34E7	3.30E8 ± 2.65E7	3.43E8 ± 9.13E7	3.23E8 ± 6.69E7
	**% Reads Mapped**	81.49 ± 0.09	99.29 ± 0.11	99.29 ± 0.08	98.80 ± 0.60	96.84 ± 4.75
	**Whole-genome (X)**	NA	3.56 ± 2.95	23.02 ± 10.52	23.33 ± 11.25	22.27 ± 10.81
	**HET SNP sensitivity**	NA	0.58	0.93	0.91	0.91
**HiRise**	**QC-passed Reads**	3.30E7 ± 6.86E6	5.11E7 ± 3.36E7	3.33E8 ± 2.66E7	3.47E8 ± 9.39E7	3.33E8 ± 6.14E7
	**Reads Mapped**	2.71E7 ± 6.30E6	5.07E7 ± 3.34E7	3.30E8 ± 2.65E7	3.43E8 ± 9.13E7	3.23E8 ± 6.69E7
	**% Reads Mapped**	82.37 ± 0.09	99.29 ± 0.11	99.29 ± 0.08	98.80 ± 0.60	96.84 ± 4.75
	**Whole genome (X)**	NA	3.56 ± 2.95	23.02 ± 10.52	23.33 ± 11.25	22.27 ± 10.81
	**HET SNP sensitivity**	NA	0.58	0.93	0.91	0.91
**PBJelly**	**QC-passed Reads**	3.29E7 ± 6.84E6	5.09E7 ± 3.35E7	3.31E8 ± 2.64E7	3.45E8 ± 9.29E7	3.31E8 ± 6.09E7
	**Reads Mapped**	2.71E7 ± 6.25E6	5.06E7 ± 3.33E7	3.29E8 ± 2.63E7	3.41E8 ± 9.05E7	3.22E8 ± 6.66E7
	**% Reads Mapped**	82.28 ± 0.09	99.46 ± 0.11	99.47 ± 0.08	98.97 ± 0.61	97.00 ± 4.78
	**Whole-genome (X)**	NA	3.36 ± 2.97	21.75 ± 11.46	22.04 ± 12.14	21.04 ± 11.64
	**HET SNP sensitivity**	NA	0.55	0.88	0.87	0.86

Table 7. *Sceloporus* species with partial genomic sequence assemblies updated using SceUnd1.0 as a reference. Genomic resources for 34 of the species were obtained using reduced representation libraries (Arthofer et al. 2014), while one species, *S. occidentalis*, was sequenced using whole-genome shotgun sequencing (Leaché et al. 2013). The data were downloaded from the Sequence Read Archive (Study Accession SRP041983; Genomic Resources Development Consortium et al., 2015). Gigabases refer to the amount of sequence data for each library.

		Original De Novo Assembly				*Reference-based Assembly*			
Species	**SRA Accession**	**Gigabases**	**%Coverage**	**BUSCO %Comp**	**BUSCO %Frag**	**%MAPPED**	**%Coverage**	**BUSCO %Comp**	**BUSCO %Frag**
S. occidentalis	SRX545583	40.88	61.01	16.2	32.8	96.59	88.68	90.2	5.7
S. adleri	SRX542351	6.14	0.88	0	0	94.18	63.2	25.8	23.3
S. angustus	SRX542352	5.9	1.18	0.1	1.1	74.73	46.43	33.0	27.7
S. bicanthalis	SRX542353	5.1	1.74	0.2	1.6	92.52	42.26	7.0	19.5
S. carinatus	SRX542354	7.96	1.38	0.2	1.2	75.11	46.47	31.7	31.1
S. clarkii	SRX542380	3.92	0.08	0.0	0.0	86.84	15.71	0.8	3.0
S. cowlesi	SRX542355	4.93	3.78	0.2	3.1	97.88	60.17	13.7	21.6
S. edwardtaylori	SRX542356	4.57	1.37	0.1	1.4	95.94	58.21	13.8	20.8
S. exsul	SRX542357	3.57	0.04	1.7	0.3	80.2	52.16	6.0	16.3
S. formosus	SRX542358	6.5	1.81	0.1	1.7	96.19	70.49	39.1	27.1
S. gadoviae	SRX542359	5.82	1.06	0.2	0.9	87.34	40.13	4.4	14.8
S. graciosus	SRX542383	4.53	NA	0.1	0.4	84.72	7.13	0.1	0.4
S. grammicus	SRX542360	4.76	1.81	0.1	1.7	92.92	52.8	12.2	20.7
S. horridus	SRX542361	3.74	0.17	0.2	0.9	95.92	37.49	1.6	7.0
S. hunsakeri	SRX542362	4.42	1.14	1.8	0.9	83.3	38.41	2.8	10.6
S. jalapae	SRX542363	6.96	1.5	0.0	0.0	88.12	56.49	34.4	31.0
S. licki	SRX542364	3.38	0.95	1.4	1.0	93.31	36.81	2.1	9.1
S. magister	SRX542365	3.5	0.8	1.7	0.7	84.26	31.74	1.2	5.6

S. malachiticus	SRX542384	4.55	0.11	0.1	0.4	91.15	22.27	0.9	4.2
S. mucronatus	SRX542366	5.54	1.25	0.2	1.4	94.23	60.02	20.9	25.3
S. ochoterenae	SRX542367	6.63	1.57	0.3	2.5	78.84	46.78	17.6	21.6
S. olivaceus	SRX542368	3.14	1.11	1.2	0.9	95.38	35.89	1.4	8.2
S. orcutti	SRX542369	3.88	0.99	1.8	0.9	81.14	35.79	1.9	8.8
S. palaciosi	SRX542370	6.59	1.58	0.1	1.5	90.49	42.11	3.4	11.3
S. scalaris	SRX542371	6.56	1.04	0.2	1.8	89.93	65.53	47.0	24.9
S. smithi	SRX542373	4.75	1.18	0.1	0.8	77.35	39.47	7.7	16.8
S. spinosus	SRX542374	5.91	1.51	0.1	1.1	96.8	69.15	36.0	26.9
S. taeniocnemis	SRX542382	3.68	0.14	0.1	0.4	88.58	22.35	0.9	3.7
S. torquatus	SRX542375	6.78	1.75	0.3	2.2	90.15	57.36	20.1	21.4
S. tristichus	SRX542376	5.36	4.67	0.3	3.4	98.29	62.09	17.4	22.8
S. utiformis	SRX542381	4.13	0.06	0.0	0.3	63.97	17.42	1.1	3.7
S. variabilis	SRX542377	7.59	1.5	0.2	1.2	76.93	52.22	38.8	30.2
S. woodi	SRX542378	3.52	0.7	1.7	0.8	94.64	52.36	6.4	17.9
S. zosteromus	SRX542379	2.71	0.62	1.3	0.9	93.48	29.39	0.7	5.3
Average (excluding S. occidentalis)			1.23%				44.4%		

Genomic Resources Development Consortium, Arthofer W., Banbury B.L., Carneiro M., Cicconardi F., Duda T.F., Harris R.B., Kang D.S., Leaché A.D., Nolte V., Nourisson C., Palmieri N., Schlick-Steiner B.C., Schlötterer C., Sequeira F., Sim C., Steiner F.M., Vallinoto M., Weese D.A. 2014. Genomic resources notes accepted 1 August 2014–30 September 2014. Molecular Ecology Resources. 15:228–229. https://doi.org/10.1111/1755-0998.12340

Leaché, A.D., Harris, R.B., Maliska, M.E. and Linkem, C.W., 2013. Comparative species divergence across eight triplets of spiny lizards (Sceloporus) using genomic sequence data. Genome Biology and Evolution. 5:2410–2419.

 Click here to access/download;Figure;Figures_2021-04-16.docx

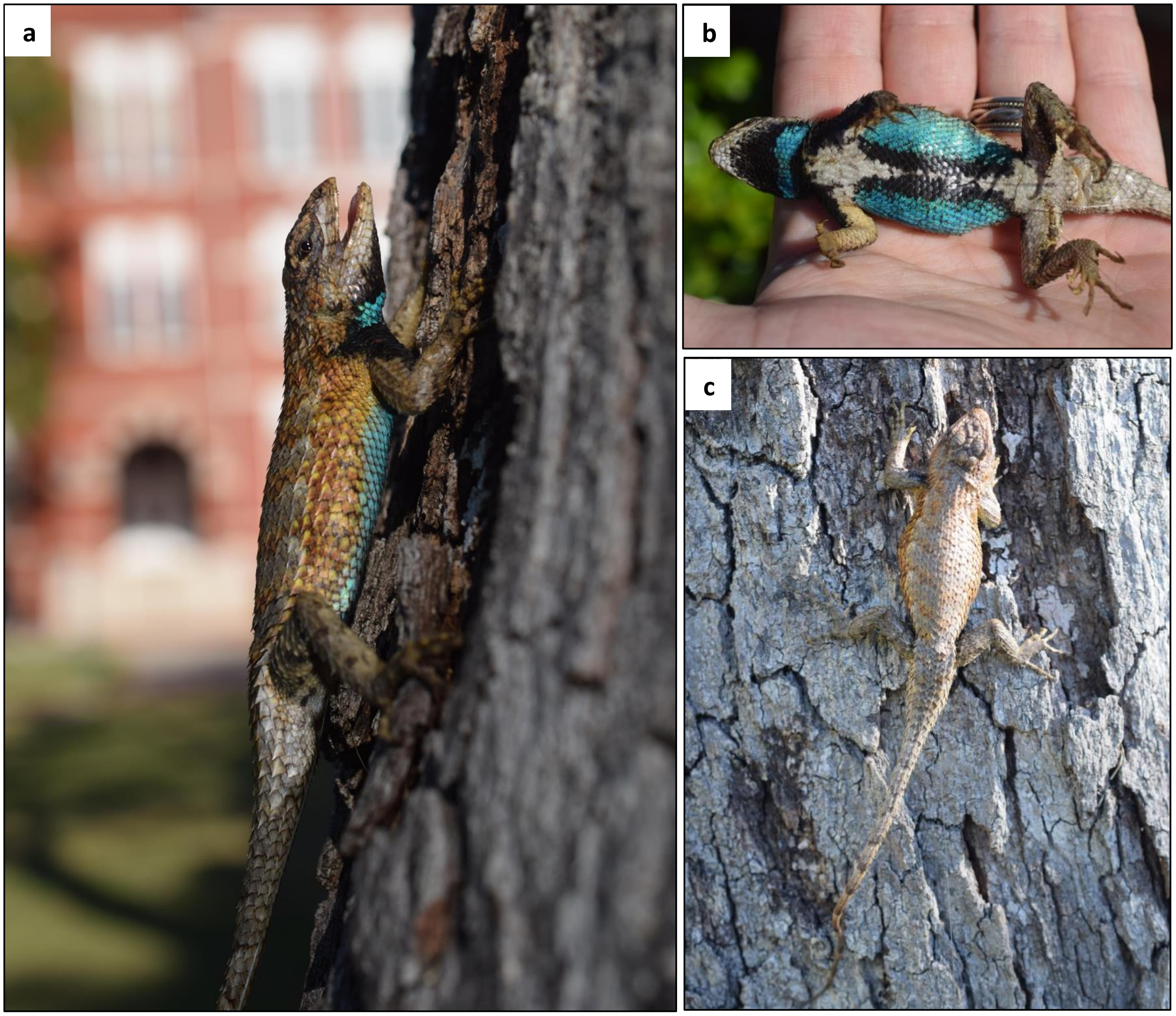

Figure 1: Adult male *Sceloporus undulatus* (Eastern Fence Lizard) from Andalusia, Alabama, pictured outside of Sanford Hall at Auburn University, (a) profile, (b) ventral, (c) dorsal view. This specimen was used for genome sequencing at DoveTail Genomics. Photo credits to R. Telemeco.

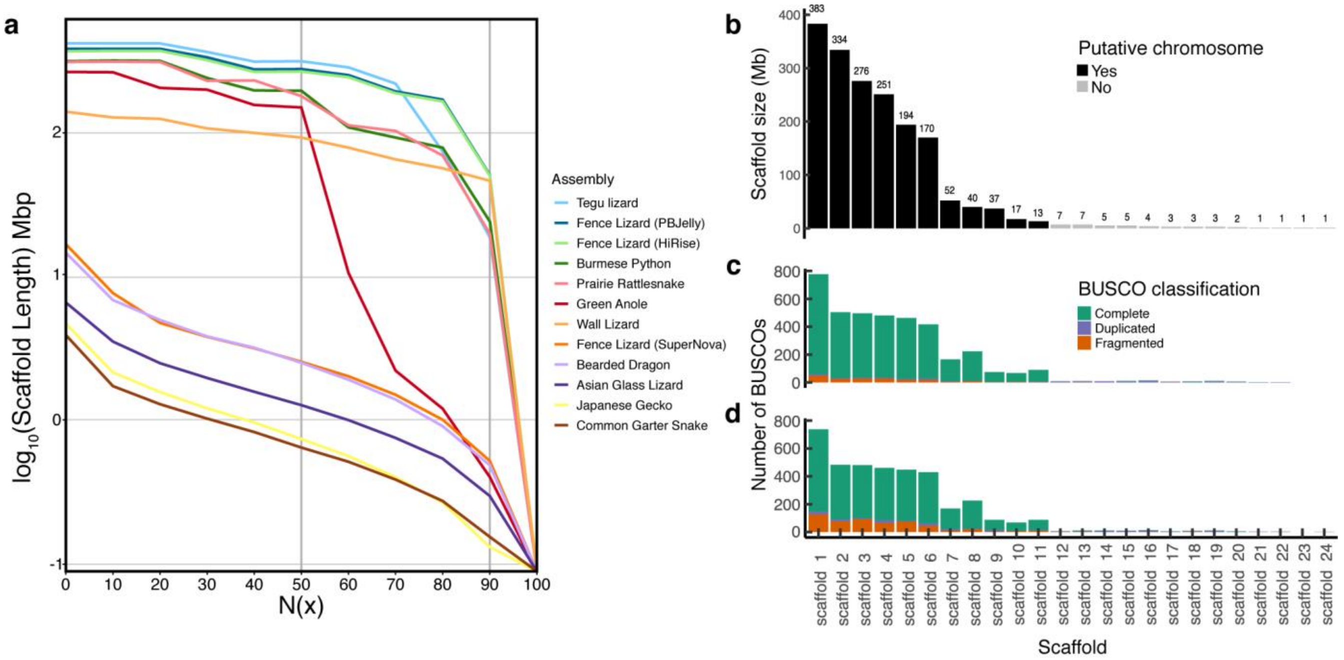

Figure 2: An evaluation of *Sceloporus undulatus* genome assembly quality. (a) Comparison of the contiguity of the three *S. undulatus* genome assemblies (Fence Lizard) relative to other squamate genome assemblies based on the log 10 of the scaffold length. The X axis is the N(x) with the N50 and the N90 emphasized with a vertical line, representing the scaffold size that contains 50 or 90 percent of the data. The legend lists the assemblies in the order of the lines from most contiguous (top) to least contiguous (bottom). Note the Fence Lizard PBJelly (dark blue, SceUnd1.0) and Fence Lizard HiRise (green) assemblies are the second and third from the top and are nearly indistinguishable. (b-d) Scaffold size distribution of SceUnd1.0 and the number of BUSCO genes that mapped to each scaffold. (b) The length of the first 24 scaffolds, where the first 11 scaffolds likely represent the haploid N=11 chromosomes (6 macrochromosomes and 5 microchromosomes). The numbers above each bar represent scaffold length to the nearest Mb. The number of BUSCO genes that mapped to each scaffold based on (c) the genome assembly, and (d) the predicted proteins from the annotation. The 11 large scaffolds inferred to correspond to chromosomes have many unique and complete BUSCO genes (green), whereas the smaller contigs have duplicated BUSCOs (purple) suggesting they are the result of reads not mapping correctly to the chromosomes.

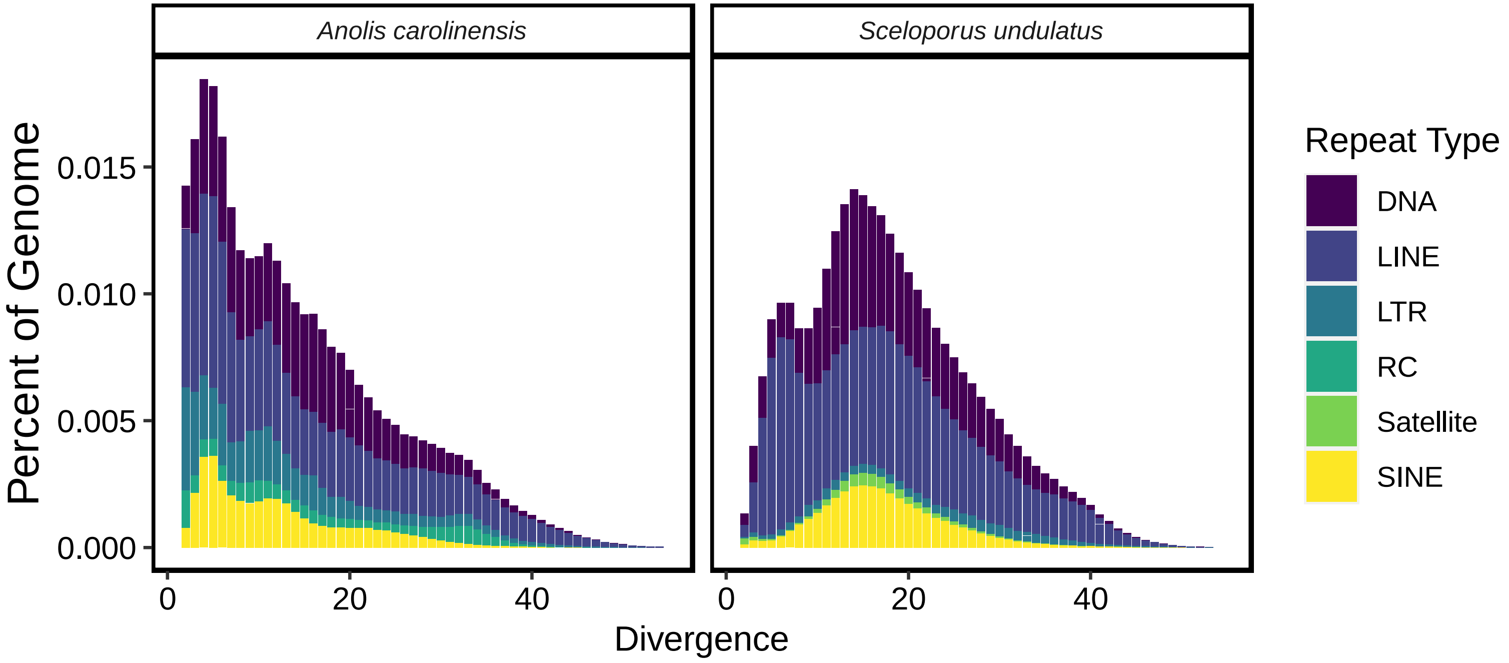

Figure 3: Age distributions of the major repetitive elements found in the *Anolis carolinensis* (AnoCar2.0) and *Sceloporus undulatus* (SceUnd1.0) genome assemblies. The repeat landscapes depict the relative abundance of repeat types in the genome versus their Kimura divergence from their consensus. DNA=DNA transposons; LINE=Long Interspersed Nuclear Element; LTR=Long terminal repeat retrotransposons; RC=rolling circle Helitron; SINE= Short Interspersed Nuclear Element.

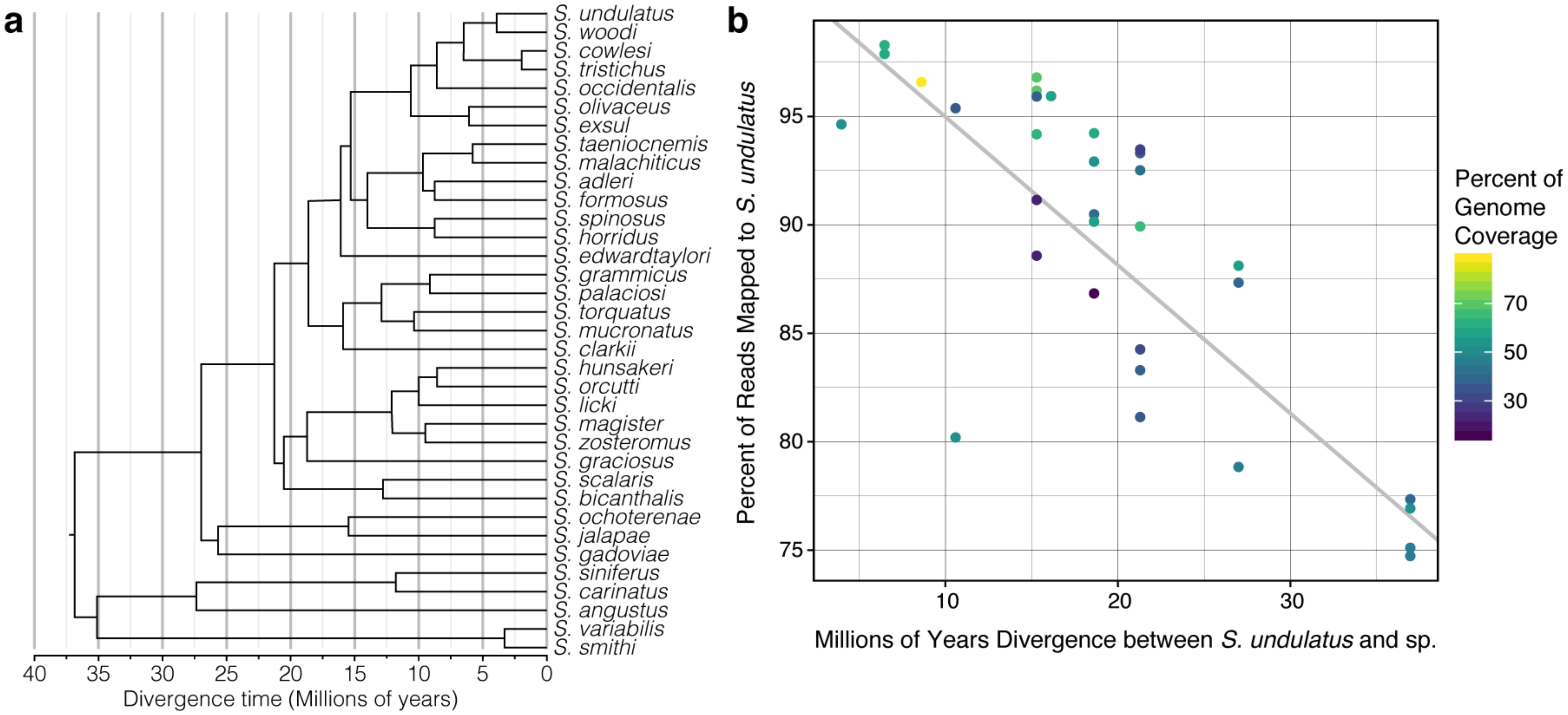

Figure 4: Relationship between divergence time and effectiveness of using the *Sceloporus undulatus* assembly for reference-based mapping. (a) A phylogenetic tree of *Sceloporus* species with draft genomic data. Species groups' names are included for the groups closest to *S. undulatus*. (b) Mapping each species by % reads mapped and time of divergence from *S. undulatus* with a linear regression. The color of the dots represents the percent of the genome that is covered, which was affected by the number of redundant sequences in the reduced representation library for a particular species.

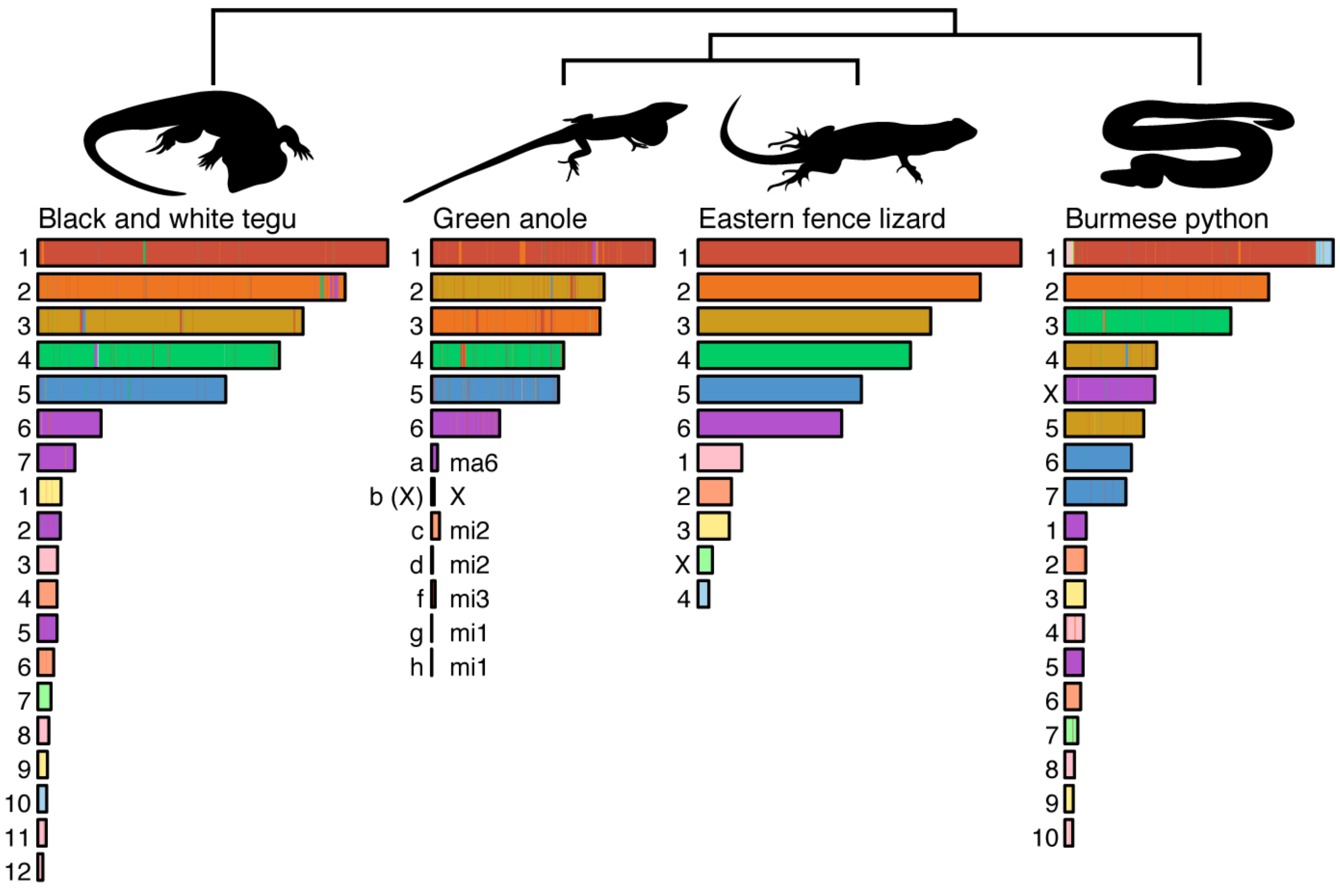

s

Figure 5: Marker-based synteny painting of fence lizard (*Sceloporus undulatus*) scaffolds/chromosomes onto the tegu (*Salvator merianae*), green anole (*Anolis carolinensis*), and python (*Python bivittatus*) assemblies. The color indicates synteny for that scaffold. The linkage groups representing macrochromosomes and microchromosomes are numbered independently for each species. Green anole linkage groups are labeled with lowercase letters, and the syntenic fence lizard chromosomes are listed to the right. Putative sex chromosomes are indicated with uppercase letters.

Click here to access/download
Supplementary Material
Supplemental_Methods-Results_2021-04-16.docx

Dear Editors and Reviewers,

Thank you for the review of our manuscript. We found the reviews insightful and responding to the comments have allowed us to improve the manuscript considerably. Below we have addressed every comment in Blue, and added or edited text in *Blue italics,* and when we have made changes in the manuscript we indicate the page and line numbers as well as use tracked changes in the manuscript. We think you will find these revisions have improved the manuscript.

Thank you,
Tonia

Reviewer #1: The manuscript "A chromosome-level genome assembly for the Eastern fence lizard (Sceloporus undulatus), a reptile model for physiological and evolutionary ecology" by Westfall et al, reports a genome assembly that is likely to be of use to labs working in this and other reptilian systems. There are a few aspects of the presentation that are a bit confusing or are otherwise in need of revision, but I suspect these can be resolved. Specific comments are outlined below.

1) It in the context of the way the manuscript is laid out it would have been interesting to see the results of a PacBio only assembly, and/or an assembly that intergrated PacBio at an earlier stage, similar to the VGP assembly pipeline (https://vertebrategenomesproject.org/phase-one). Given the timescale of resource development and use of DoveTail (see also comment 2) I can understand why the reported approach was used, though it may not have been optimal.

Response: Thank you for this comment. We agree that comparing different combinations of the order in which we used the data types would be interesting. Unfortunately, because of how our current data was collected these would not be valid comparisons, particularly because the low level of coverage with the PacBio reads were collected specifically to complement the previous assembly and not sufficient for *de novo* assembly.

2) The use of HiRise (Dovetail) in the assembly pipeline raises some issues with reproducibility as that program is maintained as closed source code. As such it will be impossible for anyone to independently replicate the published assembly using the same methods reported in the paper. This may change in the future if the code is released, and I encourage the authors to request its release. If this request is not granted the authors should make sure to include the software version used for this assembly and all relevant assembly/filtration parameters, as well as a .agp (or similar) file that relays mapping evidence and weights that were used in the scaffolding process.

Response: As correctly indicated by the reviewer the HiRise Pipeline is proprietary to DoveTail Genomics, who did the sequencing and assembly for that portion of this project. Unfortunately,

upon contacting DoveTail Genomics they notified us that the production files older than 3 years are purged from their system so the specific run files are not available. Therefore, we are not able to provide those requested files. We have included the HiRise version number and parameters in the main text (see below) for the readers to evaluate.

Page 5, line 127, text added:
"The data from both Hi-C and the 10X Genomics were used for assembly in the HiRise software (v2.1.3-5ce4af34ac25) pipeline at DoveTail Genomics. The pipeline excludes contigs/scaffolds < 1kb and only uses MQ>50 reads for scaffolding. The reads were aligned with a modified SNAP pipeline."

3) It is unclear to me if the assignment of the sex chromosome is valid. It appeals that the only evidence used to support this is homology to the anole sex chromosome. In a lineage with variable sex chromosomes it would seem that this would provide scant evidence in favor of a particular chromosomes being the sex chromosome. Is there any other information that can be leveraged here? If not I would recommend dropping this section and the paragraph immediately preceeding the discussion.

Response: Thank you for this comment, we now see how this section can be clarified, and we have added text to clarify this point (see below). We have included additional information in this section for why we think this approach would likely identify the sex chromosome. But, we have also added "putative" in multiple places including Figure 5 to emphasize that this finding needs to be empirically confirmed. The paragraph above the discussion now describes the results from an additional, separate synteny analysis that further supports our original inference that the fourth predicted microchromosome is the putative X chromosome.

Page 9, line 266, the following text has been added to the paragraph describing the annotation of the putative X chromosome*:*

"These heteromorphisms are likely the result of other chromosomes' fusions to the X, as Sceloporus are among the large portion of iguanian lizards with conserved sex chromosomes, and another Sceloporus species within the same broad 2n=22 radiation, Sceloporus malachiticus, has an X chromosome homologous to the green anole X, but fused to several microchromosomes [68]. Given the observed homology, we used known X chromosome genes from the green anole to identify the scaffold likely representing the X chromosome within S. undulatus independently from other synteny analysis."

Page 9, line 279:

"This result, that the fourth predicted microchromosome is the putative X chromosome, is further supported by a separate synteny analysis described below"

Page 14, line 457, we have edited this text in the last paragraph above the discussion to read:

“These synteny results further support that the fourth largest microchromosome in the SceUnd1.0 assembly is syntenic to the anole X chromosome (Figure 3, Figure 5). However, it is not syntenic to the python X chromosome, which is syntenic to the Z chromosome in other snakes. The tegu sex chromosome has not been identified. Based on the blast hits from the anole X-linked genes and this synteny analysis we define the fourth largest microchromosome in the SceUnd1.0 assembly as the putative X chromosome, but functional data are needed to confirm this assignment.”

We have also edited the paragraph at the top of page 14 that further discusses the fusion and fission of chromosomes in squamates.

4) The presentation of data from the 34 additional species could also use some shoring up, particularly given the fact that these mostly rely on reduced representation data that should subsample ~2% of the genome (notably, the guess of 2% may be wrong here, can the authors provide an estimate). How much overlap is there in the physical regions sampled in each of the 34 species? Is it nearly the same 2% or are they sampling wildly different intervals? This is likely to be of interest to several groups that are using reduced representation sequencing for comparative studies. The new genome assembly uniquely positions the authors to address this and they seem to already have the necessary data in hand.

Response: We have included additional analyses for the reader to better understand the overlap among the 34 Sceloporus reference-based assemblies. We used bedtools to count (1) the number of bases that have coverage by a specific number of reference-based genome assemblies (1 to 34 of the assemblies), and (2) the accumulating fraction of the genome that is covered by 1 to 34 reference-based assemblies. We provide these as bar graphs in Supplemental Figure 4 (Figure S4). We also provide an example of how these data can be used by focusing on one gene of interest to our group, IGF1. Using the coordinates in the annotation in the .gff3 from the SceUnd1.0 we pulled out the exons for the target gene, IGF1. These were aligned and visualized in Geneious. We found 16 of the 34 species had >75==% coverage across the protein coding region of this gene, 24 of them had >50% coverage. This is sufficient coverage to calculate estimates of genetic variation, selection analysis, and gene tree comparisons.

Page 13, Line 421 we edited the text to read:
“*However, together these draft genomes contain a substantial amount of data that can be used for comparative genomic analyses. Figure S4 demonstrates the overlap in coverage of SceUnd1.0 by the reference-based genome assemblies. These distributions estimate that 50% of the genome would be covered by a subset of 16 species. Focusing on one individual gene of interest to our group, IGF1, we found that 16 of the 34 species had >75% coverage across the protein coding region of this gene and 24 of 34 had >50% coverage (Figure S4). Therefore, this dataset should prove useful for analyses of protein and gene sequence evolution to understand behavioral ecology, physiology, developmental biology, and more.”*

5)	Related to this, for cases where substantially more than 2% of the genome is corrected. How is this distributed? In repetitive elements? Single-copy sequence? And why does this deviate from the expected ~2% in the cases that is does (e.g. 44% in one case).

Response: The 2% estimate is based on in silico experiments. It seems there was considerable by-catch in much of the reduced-representation sequencing, which is normally filtered out when those reduced representation data are being analyzed. With this by-catch, we were able to recover a reasonable amount (20-40%) of the genome sequence at low (1-3X) coverage for most species. We plotted the distribution of coverage across the genomes for each species to demonstrate the low-level coverage (Figure S3).

Page 12, Line 402: We have edited to the text in this section to the following
"*It seems there was a considerable amount of by-catch in much of the reduced-representation sequencing that is normally filtered out when those reduced representation data are being analyzed. For the species with ~5Gb of sequencing data, we improved the genome coverage from an average of 1.23% to an average of 44.4% coverage at low depth (1-3X) (Figure S3). For S. occidentalis with ~ 41Gb of data, coverage improved from 61.0% to 88.7% (Table 7) at an average depth of ~ 20X (Figure S3).*"

6)	The authors cite reference 17 in support of the need to sample additional squamates. This reference focuses on repetitive elements, and is therefore notable that the authors do not report and data on the distribution or density of repeats. An analysis of repeat content/classification was likely done as part of the annotation pipelines and I think a brief summary would be a welcome addition to the report.

Response: Thank you for this suggestion. We have conducted a repeat analysis and have included it as an additional text section in the manuscript starting on Page 9, as well as a figure (Figure 3) and a supplementary table (Table S4) that describe the distribution of repeat elements in *S. undulatus* with an evolutionary contrast to *A. carolinensis*.

Minor comments:
Line 102 - the species name is misspelled
Response: Thank you for catching this typo, it is now corrected.

Line 381 - species name is not italicized
Response: Thank you for catching this typo, it is now corrected.

Reviewer #2: The authors represented us a genome assembly of Sceloporus undulates, which utilized three sequencing technologies including 10X linked reads, Hi-C data, and long reads from the PacBio sequencing platform. I totally agree with the authors to one of their main conclusions that the SuperNova assembly was sufficient for mapping RNAseq and whole-genome resequencing. Although such a conclusion could have long been recognized, it is

helpful that the authors can prove it using this new dataset. In addition, a high-quality genome of S. undulates is a good addition to what we have for the squamate genomic data. Overall the manuscript is well developed. However, there are a couple of points that the authors should acknowledge and discuss:

Major:

(1) Please provide several important assessments to validate the genome assembly accuracy. For instance, genome size should be estimated to check whether the assembly can represent the entire genomic info or contains redundancies; A GC-depth plot should be included to demonstrate whether the assembly contains contaminations from other species; Contig N50 (different form scaffold N50) should be added to demonstrate the assembly contiguity level.

Response: Thank you for these suggestions. We have now provided citations for genome size estimates for related species for comparison, as well as a GC-depth plot to test for contamination from other species, which appears negligible. We added Contig N50 to Table 1.

Page 5-6, starting on line 153, we have added the following text:
"Estimated genome size of the closely related species Sceloporus occidentalis is 2.36GB based on fulcan densiometry.   Assuming S. undulatus is similar, the 1.9GB of sequence in our SceUnd1.0 assembly is likely either missing some data, or repeat regions have been condensed, creating redundancies. To assess the level of contamination in our SceUnd1.0 genome assembly we used Blobtools (v1) (Laetsch DR and Blaxter ML, 2017) workflow A that estimated contamination based on GC content difference that exist between taxa. In order to visualize depth by GC content for taxa represented in the assembly, we created a blobDB using a BAM file to infer coverage, sequence similarity hits based on the DIAMOND blast, and the SceUnd1.0 assembly fasta file. Plots were produced for two taxonomic ranks, phylum and order, with taxonomic annotation based on the "bestsum" taxrule. The majority of the represented taxa in the assembly were annotated as belonging to Chordata (phylum level) and Squamata (order level). There is a smaller, but visible, proportion of reads that are associated with order Testudines, which is likely due to regions of sequence similarity across reptiles. Overall, the plot demonstrates negligible contamination of other taxa (Figure S1)."

(2) The authors claimed that they conducted reference-based assemblies for 34 Sceloporus species to improve draft nuclear genomes assemblies from 1% coverage to 43% coverage. This information is rather misleading. Firstly, De novo assembly can hardly represent a widely accepted method when dealing with the reduced presentation data (also known as RAD-Seq or GBS data). Therefore, such a comparison doesn't mean much. Secondly, the authors should tone down this part, as readers would expect to make use of them directly when the authors mentioned them as draft genome assemblies. However, I can hardly find a scenario in which those assemblies can be reused directly. I would choose the S. undulates genome as a reference, instead of any those "draft genome assemblies", to conduct, for example, resequencing-based population studies.

Response: The goal of the reference-based assemblies was not intended to be a comparison to the *de novo* assemblies to determine which is a better method, but rather to demonstrate improvement of the assemblies as a resource for the community. These reference-based assemblies are not necessarily intended to be used as References in of themselves. The reviewer is correct that SceUnd1.0 would be the best assembly to map to (we have removed that phrase of the sentence). Nonetheless, these draft genomes contain a significant amount of data that can be used for comparative genomic analyses. We have provided additional analyses to demonstrate the number of genomes that can be sampled for a particular fraction of the genome, see Figure S4. These analyses demonstrate that, for ~50% of the bases in the SceUnd1.0 assembly, 16 species would be represented. We also provide an example, focusing on one gene of interest to our group, IGF1, we found that 16 of the 34 species had >75% coverage across the protein coding region of this gene, and 24 of them had >50% coverage. This is sufficient coverage to calculate estimates of genetic variation, selection analysis, and gene tree comparisons.

Page 13, Line 421 we edited the text to read:
"*However, these draft genomes contain a substantial amount of data that can be used for comparative genomic analyses. Figure S4 demonstrates the overlap in coverage of SceUnd1.0 by the reference-based genome assemblies. These distributions estimate that 50% of the genome would be covered by a subset of 16 species. Just focusing on one gene of interest to our group, IGF1, we found that 16 of the 34 species had >75% coverage across the protein coding region of this gene, and 24 of them had >50% coverage (Figure S4). Thereby, this dataset should prove useful for analyses of protein and gene sequence evolution to understand behavioral ecology, physiology, developmental biology, and more.*"

(3) the authors claimed that the addition of PB long reads data provided negligible gains. PBJelly mainly works for gap-closing, so there might be a significant improvement in Contig N50 value, which can only be confirmed after the authors provide such data. In addition, the authors could use some other software, such as this one (http://biorxiv.org/content/10.1101/831248v1.full), which claimed to perform ten times better than PBJelly.

Response: We have added the Contig N50 to Table 1. Thank you for the suggestion to use the program presented in the BioRXiv link. We do not intend to reassemble and redo the analyses for this manuscript as other manuscripts are already in preparation or have been submitted that have used the current SceUnd1.0 Assembly. But we will definitely consider using the reference program in future versions as we continue to improve further versions of this genome reference.

Minor:

(4) Line 131: it is better to provide the genome coverage information (for example, 40X) in the main text as well.

Response: Thank you for this suggestion, it has been incorporated.

(5) line 163: scaffolds as long as 7 MB might not be mentioned as tiny scaffolds.

Response: Thank you for this suggestion. We have changed “tiny” to “smaller”.

(6) Line 179 - 181: the authors may obtain useful information from this paper, 10.1093/gigascience/giy163, which demonstrates the potential problems regarding the 10X genome assembly - generate lots of gaps and produce redundancies.

Response: Thank you for bringing this reference to our attention. While it is an interesting paper, we are uncertain what action (if any) is being requested by this comment in the context of revising the manuscript.

(7) line 259: the authors may want to give some clues for how to improve the genome annotation. Plus, the authors may provide some details on why those missing BUSCO genes failed to be annotated, was it attributed to sequence errors which cause nonsense mutations? If so, genome polishing may correct them.

Response: The SceUnd1.0 has been submitted to NCBI and is in line for their annotation pipeline and then it will be moved into ENSEMBL. We suspect this pipeline would improve the annotation. As this genome assembly is already being used for multiple projects, with manuscript preparation, undoubtedly efforts will continue to improve upon this assembly. In terms of the missing BUSCOS, the BUSCOs were for Tetrapoda and it is possible some of the genes are truly missing in S. undulatus. Because there were more BUSCOs fragmented or missing from the predicted proteins (the annotation) than the actual genomic sequence itself, we attribute those to annotation errors, not errors in the assembly.

Page 8, line 255 we have edited this text to read:

“Because there were more BUSCOs fragmented or missing from the predicted proteins (the annotation) than the actual genomic sequence itself, we attribute those to annotation errors, not errors in the assembly, which suggests this first version of annotation can be improved.”

(8) Line 278: For the mitogenome assembly, there is a big chance that de novo RNAseq assembly can obtain whole or part of the mitochondrial genome. It won't be hard work, and it is interesting to examine whether reference-based mito-genome assembly can introduce some unexpected bias.

Response: We used the program MitoZ to de-novo assemble the mitochondrial genome using RNA-seq paired-end sequencing data from 18 individuals (of the same population). We used several techniques to go about performing this assembly:

1) Assembly for each individual using the Quick Assembly approach without a taxonomic constraint
2) Assembly for each individual using the Multi-Kmer approach without a taxonomic constraint
3) Assembly for each individual using the Quick Assembly approach with a taxonomic constraint (Phrynosomatidae)
4) Assembly for each individual using the Multi-Kmer approach with a taxonomic constraint (Phrynosomatidae)
5) Assembly of all combined reads using the Quick Assembly approach
6) Assembly of all combined reads using the Multi-Kmer approach
7) Assembly of only those reads that mapped to the Sceloporus occidentalis mitochondrial genome using the Quick Assembly approach
8) Assembly of only those reads that mapped to the Sceloporus occidentalis mitochondrial genome using the Multi-Kmer approach

None of these approaches recovered the full mitochondrial genome (each resulted in missing protein coding genes, tRNA, and rRNA). Further, different approaches sometimes selected different scaffolds as those pertaining to the mitochondrial genome. These inconsistencies and lack of data led us to the decision to retain our approach described in the original manuscript submission, wherein we mapped the raw reads to the Sceloporus undulatus mitochondrial genome, aligned this genome with Anolis carolinensis, and transferred annotations.

Page 10, line 327, we add the following sentence to the manuscript.
"While this genome is useful for understanding sequence variation and comparative genomics and phylogenetic analyses, this mitochondrial genome should not be used for examination of mitochondrial genome structure."

As for extracting the genomes from the other 34 Sceloporus species for examination of species relationships, this is not within the scope of this study and is an objective being pursued by other labs using more appropriate datasets.

(9) Line 318: can the taxonomic uncertainty be resolved using the resequencing data, e.g. you can obtain their mito-genomes via software like Novoplasty, MitoZ, et al.

Response: This question of taxonomic uncertainty of the populations in this region is currently being addressed in more depth by other research labs as is outside of the scope of this manuscript.

(10) Line 328: Can FLAGSTAT work on sam files? Or do you mean bam files?

Response: Yes FLAGSTAT can work on sam files.

(11) Line 345 - 347: How to evaluate the correctness of those increased SNPs?

Response: We are not exactly sure what this question is asking. We did not actually call SNPs with the WGS database, but used the theoretical HET SNP sensitivity metric to estimate the utility of each assembly for calling SNPs. The full WGS dataset is part of a much bigger study across *S. undulatus* populations and will be published at a later date. We try to further clarify the theoretical HET SNP sensitivity metric and how we are using it as a metric for useability of the assemblies in the main text. We have added the following text.

Page 11, Line 362:
"*The CollectWgsMetrics tool from the Picard Toolkit [85] was used to calculate genome-wide coverage of the mapped reads for each individual and assembly, and theoretical HET SNP sensitivity (a metric based on coverage and base quality distribution that estimates probability of calling a true heterozygote SNP) as a way to predict the utility of each assembly as a reference for calling SNPs at high a low coverage. "*

Line 375: "*This may be due to repetitive regions being added to the assembly by the PacBio data making it slightly less mappable.* "

(12) Line 370: 41Gb -> 40.8Gb or ~ 41 Gb
Response: Thank you for catching this typo, it is now corrected.

(13) Line 397: please clarify the criteria and methods that produce those markers of 1,000 bp in length.
Response: We have clarified this method with the following statement:

Line 436" "The SceUnd1.0 scaffolds representing the 11 putative chromosomes were each divided into 1000 bp-long sequences that excluded gapped regions to serve as markers."

(14) Line 400: > 80% identity?
Response: Thank you for catching this typo, it is now corrected.

(15) Line 443: PBJelly cannot aid in chromosome-level assembly.

Response: Good point. We have now rephrased this to be:

Now Line 487
"Our study demonstrates that the SuperNova Assembly was sufficient for mapping RNAseq and whole genome resequencing, while the more expensive data from HiC and PacBio were necessary to achieve high-level continuity and chromosome-level scaffolding in the HiRise and PBJ Assemblies."
