# Supplementary material for: A chromosome-level genome assembly for the eastern fence lizard (Sceloporus undulatus), a reptile model for physiological and evolutionary ecology
Source: Gigascience. 2021 Oct 1;10(10):giab066. doi: 10.1093/gigascience/giab066 (PMC8486681; doi:10.1093/gigascience/giab066)
Supplement: giab066_Supplemental_File [file giab066_supplemental_file.docx]

**A chromosome-level genome assembly for the Eastern Fence Lizard (*Sceloporus undulatus*), a reptile model for physiological and evolutionary ecology**

**Westfall et al.**

**Availability of Supporting Data**

This Whole Genome Shotgun project has been deposited at DDBJ/ENA/GenBank under the accession JAGXEY000000000. The assembly Sceloporus undulatus AU_SceUnd_v1.1 (a slightly updated version of SceUnd1.0 based on NCBI requirements) is version JAGXEY010000000.

Supplemental Data are hosted on the Auburn University Scholarly Repository, AUora: <https://aurora.auburn.edu/handle/11200/49988>

1. All three genome assemblies are provided as supplemental data.
   1. SuperNova assembly containing data from 10X Genomics Chromium:

GenomeAssembly_SuperNova_Sceloporus_undulatus_pseudohap.fasta.gz

- 1. HiRise assembly containing the 10X Genomics data with the addition of the Hi-C data:

GenomeAssembly_HiRise_Sceloporus_undulatus.fasta.gz

- 1. PBJelly Assembly (SceUnd1.0) containing the 10X Genomics data and the Hi-C data, with the addition of PacBio data:

GenomeAssembly_SceUnd1.0_PBJELLY.fasta.gz

1. Tissue-Embryo Transcriptomes and annotation are provided as supplemental data.
   1. TranscriptomeAssemblyAnnotation.zip folder containing
      1. Transcriptome File: TranscriptomeAssembly_Tissues-Embryo_Trinity.fasta
      2. Annotation File: TranscriptomeAssembly_Tissues-Embryo_Transdecoder.gff3
2. Truncated assembly used for the Funannotate annotation pipeline (SceUnd1.0_top24), and the annotation results are supplied as supplemental data.
   1. SceUnd1.0_top24.fasta. This file contains only the longest 24 scaffolds and they have been renamed 1-24 from longest to shortest.
   2. SceUnd1.0_top24_Annotation_FunnanotateResults.zip folder containing the following files:
      1. SceUnd1.0_top24.gff3
      2. SceUnd1.0_top24.proteins.fa
      3. SceUnd1.0_top24.transcripts.fa
      4. SceUnd1.0_top24.annotations.txt
      5. SceUnd1.0_top24_CompiledAnnotation.csv
      6. SceUnd1.0_top24.proteins.fa.report_EnsembleCombined.top.txt
3. The mitochondrial genomes and the annotation are provided as supplemental data.
   1. MitoGenomeAssembly_Sceloporus_undulatus.fasta
   2. MitoGenomeAssembly_Sceloporus_undulatus_Annotation.gff
4. The reference-based assemblies for the 34 *Sceloporus* species are provided as supplemental data.
   1. GenomeAssemblies_34Sceloporus.tar.gz
   2. Code for generating consensus sequences for each species: mkgenome_AW-AC.sh

**Full list of genes identified in the mitochondrial genome.**

Annotations from the *A. carolinensis* mitochondrial genome (17,223 bp) transferred well to the newly assembled *S. undulatus* mitochondrial genome (17,072 bp), with 13 protein coding genes (ATP6, ATP8, COX1, COX2, COX3, CYTB, ND1, ND2, ND3, ND4, ND4L, ND5, ND6), 22 tRNA regions (tRNA-Phe, tRNA-Val, tRNA-Leu, tRNA-Ile, tRNA-Gln, tRNA-Met, tRNA-Trp, tRNA-Ala, tRNA-Asn, tRNA-Cys, tRNA-Tyr, tRNA-Ser, tRNA-Asp, tRNA-Lys, tRNA-Gly, tRNA-Arg, tRNA-His, tRNA-Ser, tRNA-Leu, tRNA-Glu, tRNA-Thr, tRNA-Pro), 2 rRNA regions (12S, 16S), and a control region.

**Table S1:** Contig length statistics for *Sceloporus undulatus* *de novo* transcriptome assemblies. 4 tissues = 3 tissues (brain, skeletal muscle and embryos) + 1 tissue (liver; McGaugh et al, 2015).

|  | **1 tissue** | **3 tissues** | **4 tissues** |
| --- | --- | --- | --- |
| Minimum length | 201.0 | 201.0 | 201.0 |
| 1^st^ Quartile | 266.0 | 266.0 | 266.0 |
| Median | 382.0 | 377.0 | 375.0 |
| Mean | 829.9 | 822.4 | 781.0 |
| 3^rd^ Quartile | 808.0 | 732.0 | 711.0 |
| Maximum length | 16,776.0 | 30,410.0 | 30,258.0 |

McGaugh, S. E. *et al.* (2016) ‘Data from: Rapid molecular evolution across amniotes of the IIS/TOR network’. Dryad. doi: [10.5061/DRYAD.VN872](https://doi.org/10.5061/DRYAD.VN872).

**Table S2:** Reads mapped to *Sceloporus undulatus* *de novo* transcriptome assembly using 4 tissues.

| **Read classification** | **Counts** | **Percentage of mapped reads** |
| --- | --- | --- |
| Proper pairing | 170,981,981 | 97.10% |
| Left read only | 3,778,790 | 2.15% |
| Right read only | 1,015,874 | 0.58% |
| Improper pairing | 310,142 | 0.18% |

**Table S3:** Representation of full-length reconstructed protein-coding genes in *Sceloporus undulatus* *de novo* transcriptome, using the protein set of *Anolis carolinensis* (AnoCar2.0, Ensembl) as a reference.

| **Alignment coverage** | **Counts** | **Cumulative counts** |
| --- | --- | --- |
| 100% | 9,874 | 9,874 |
| 90% | 1,349 | 11,223 |
| 80% | 799 | 12,022 |
| 70% | 757 | 12,779 |
| 60% | 725 | 13,504 |
| 50% | 577 | 14,081 |
| 40% | 463 | 14,544 |
| 30% | 455 | 14,999 |
| 20% | 358 | 15,357 |
| 10% | 97 | 15,454 |

**Table S4:** Comparison of repeat contents of two iguanian lizard genome assemblies.

|  | ***Sceloporus undulatus* (SceUnd1.0)** | | ***Anolis carolinensis* (AnoCar2.0)** | |
| --- | --- | --- | --- | --- |
| **Repeat Type** | **Number of elements** | **Percentage of sequence** | **Number of elements** | **Percentage of sequence** |
| SINEs | 68,485,616 | 3.59% | 75,887,012 | 4.22% |
| LINEs | 281,357,165 | 14.77% | 234,058,101 | 13.01% |
| LTR elements | 25,420,051 | 1.33% | 84,049,288 | 4.67% |
| DNA transposons | 167,999,004 | 8.82% | 157,677,814 | 8.76% |
| Unclassified | 232,908,710 | 12.22% | 34,170,372 | 1.90% |
| Satellites | 11,372,293 | 0.60% | 560,153 | 0.03% |
| Total repeats in the genome | 787,622,832 | 41.333% | 591,836,683 | 32.90% |

SINEs=short interspersed nuclear elements; LINEs=long interspersed nuclear elements; LTR=long terminal repeat


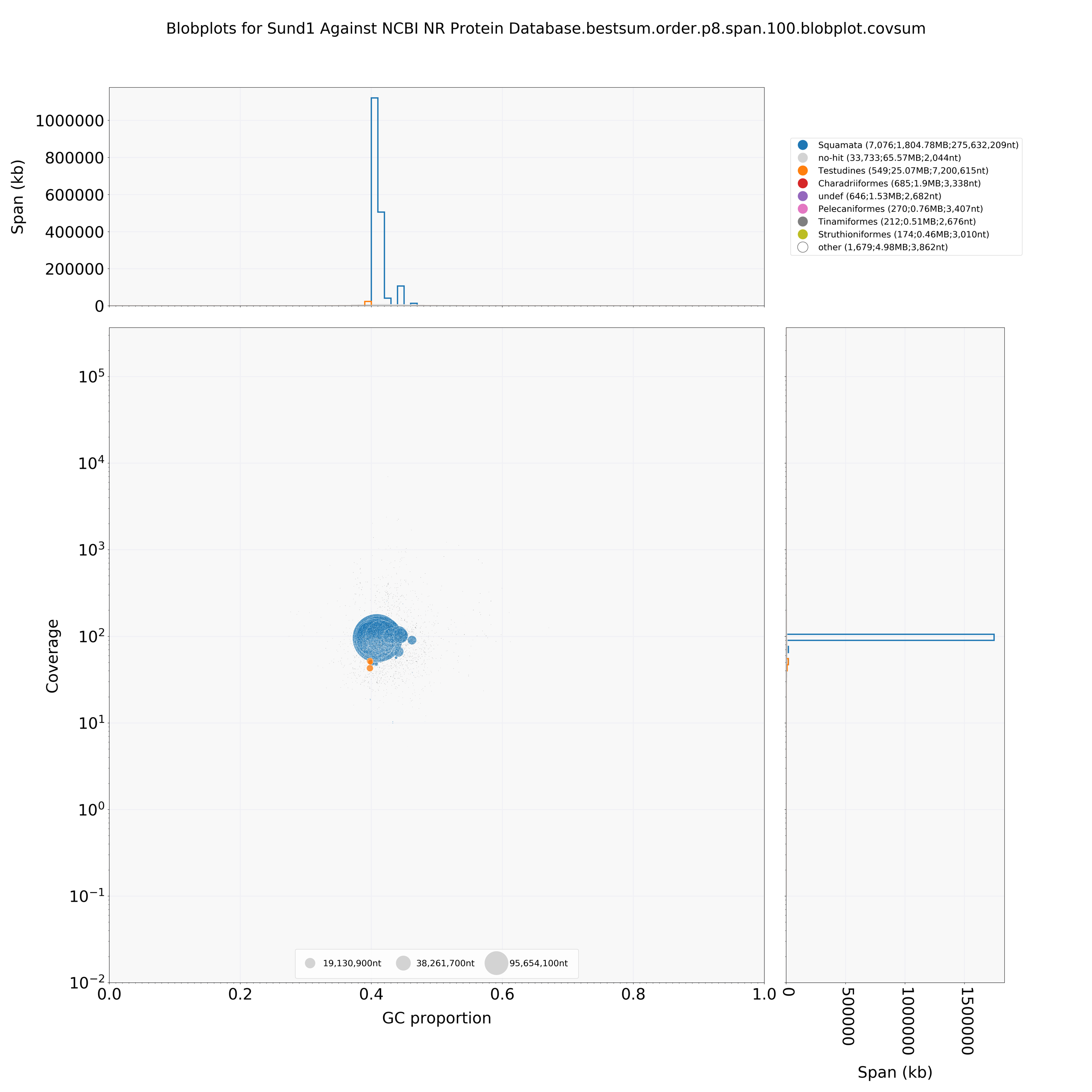
**Figure S1:** Blobplot for the SceUnd1.0. Sequences were compared to the NCBI nr protein database using BlastX. The center graph is the blobplot of coverage by GC proportion, with the top and side graphs the corresponding histograms. Taxonomic identity of the hits are plotted based on color. The size of the circles in the center plot indicates the number of sequences for a GC proportion. The low percentage of non-squamate hits were to another reptile group, Testudines.


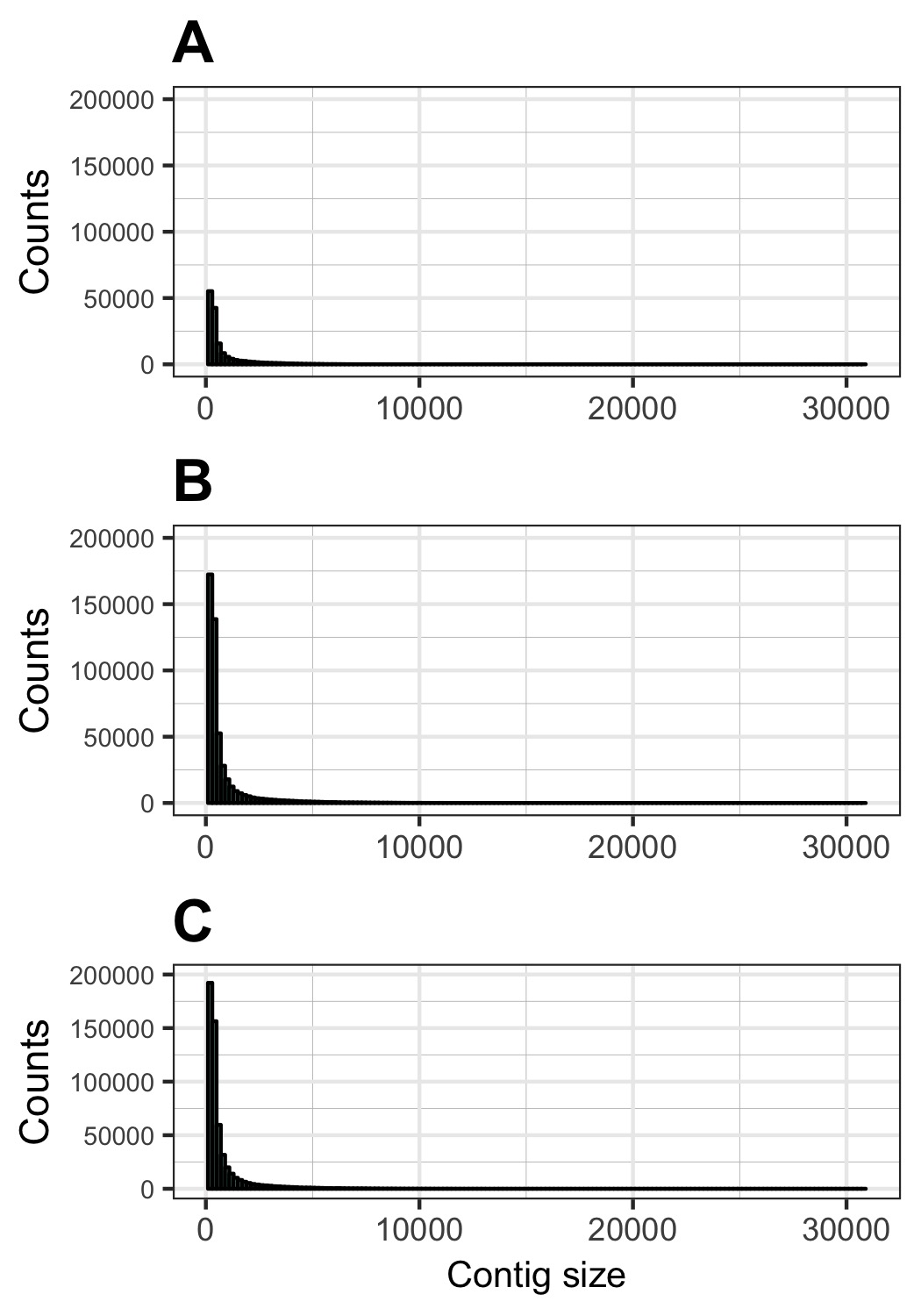


**Figure S2:** Contig sizes for different *Sceloporus undulatus* transcriptome assemblies. Assemblies used (**A**) the previously published single tissue transcriptome (liver [23]), (**B**) transcriptomes from the 3 tissues sequenced in this study (brain, skeletal muscle and embryos), and (**C**) the combined data set of 4 tissues ([23] and this study).


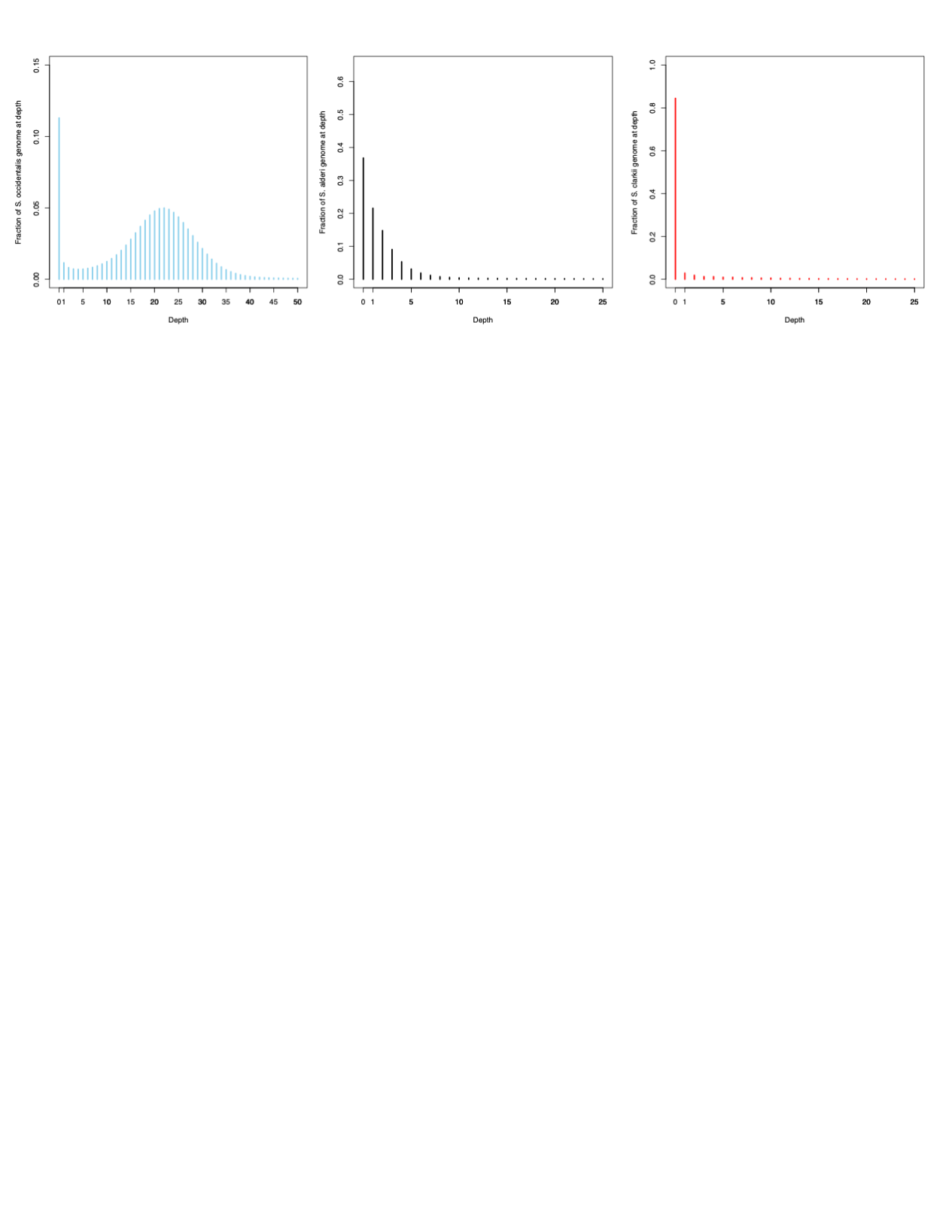

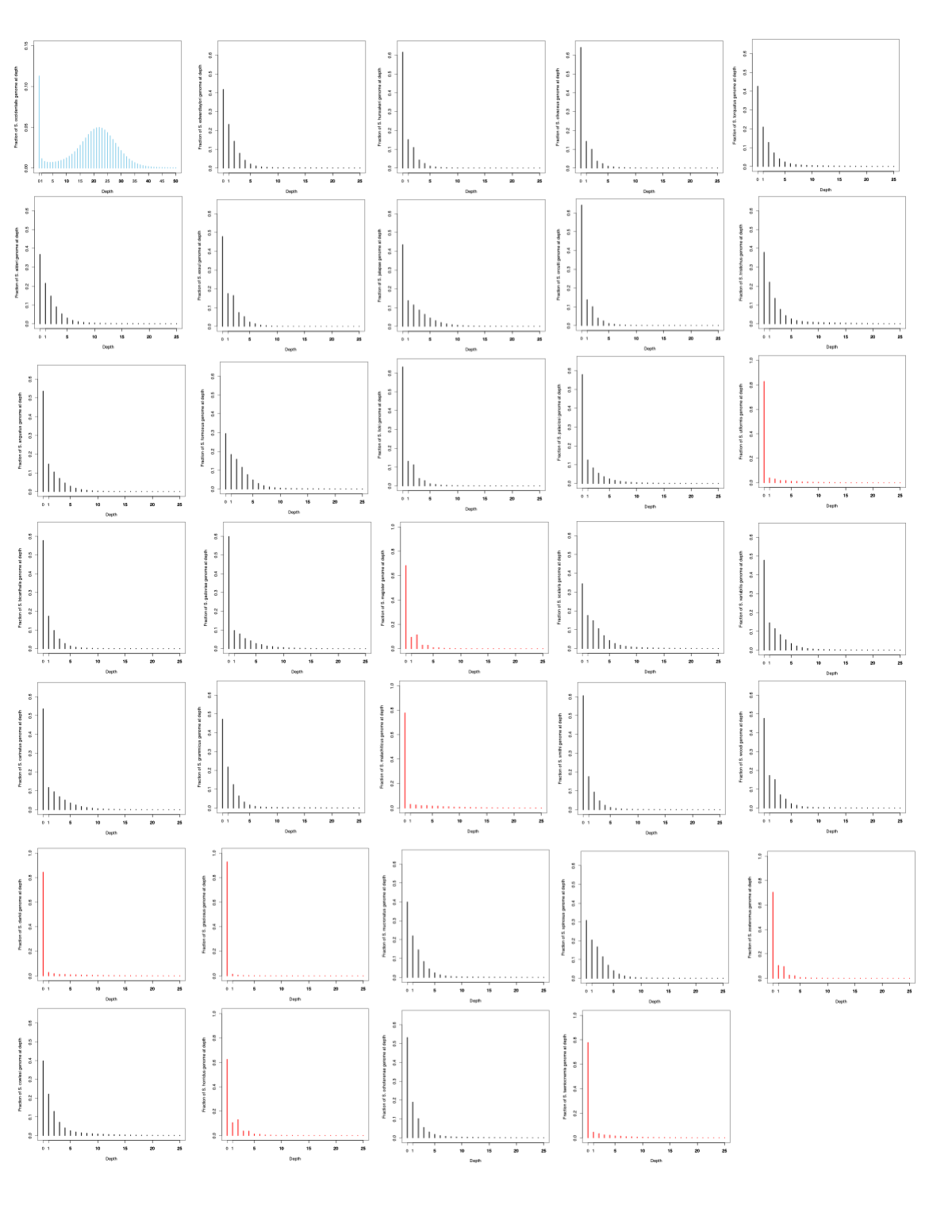


b

ab

**Figure S3:** Depth of coverage histograms for the 34 reference-based assemblies. (a) representative plots color coded by the different axes. (b) histogram for each of the species corresponding in order to Table 7, moving down and then to the right.

a

b

c


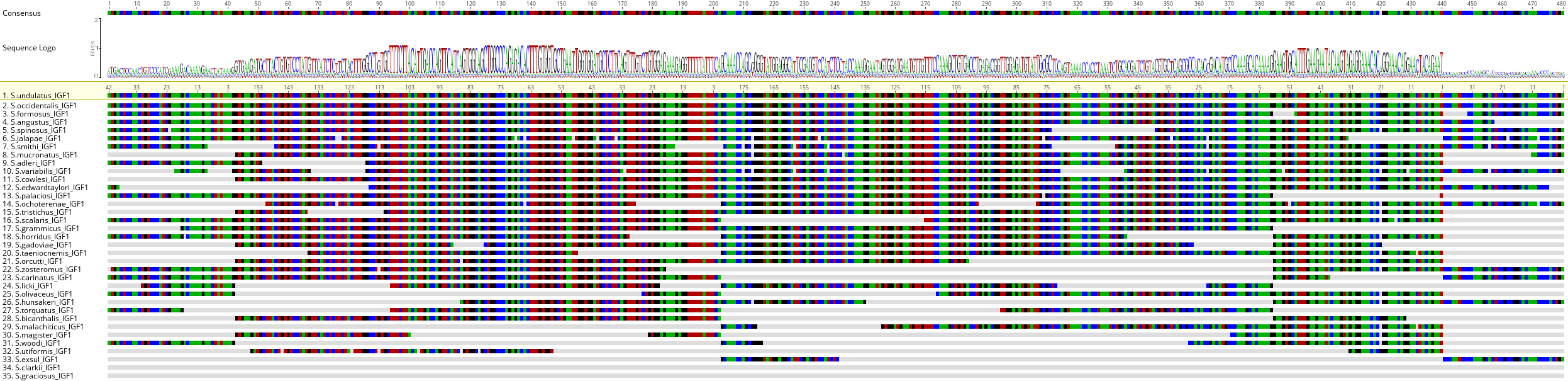

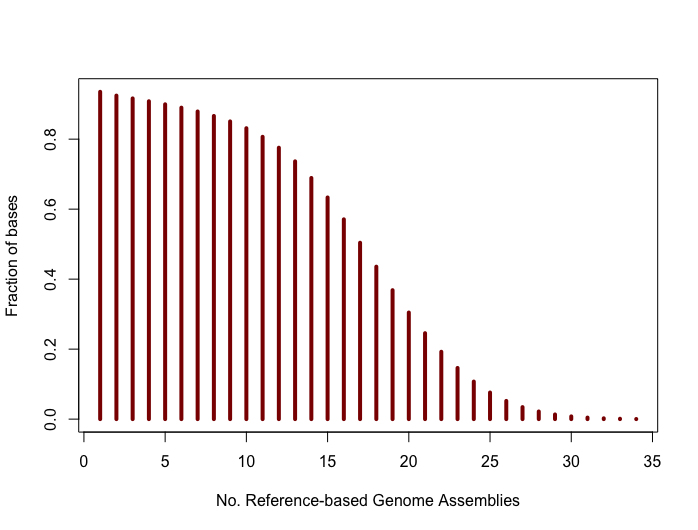

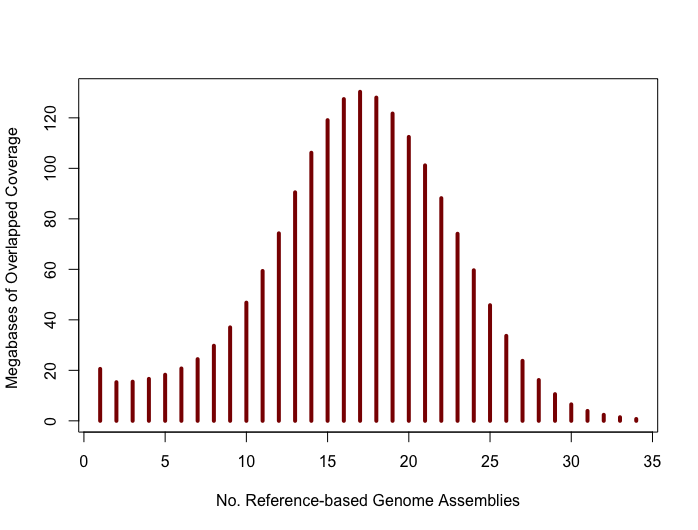


**Figure S4:** Overlap in coverage for the 34 Sceloporus species. The bar graph (a) demonstrates the number of bases (plotted as Mb) that are covered in 1, 2, 3… 34 of the reference-based assemblies. The bar graph (b) demonstrates the accumulating fraction of the SceUnd1.0 assembly that would be covered by one or more of the reference-based genome assemblies. (c) A multiple sequence alignment of the IGF1 protein-coding sequence across the 34 Sceloporus genomes with *S. undulatus* set as a reference. This provides a visual example of how the reference-based assemblies from the 34 Sceloporus species can be used. The color within the alignment represents sequence data, the gray is missing data filled with Ns. The species are sorted from most coverage of IGF1 to the least, with the last two species not having any sequence data for IGF1.
