# Supplementary material for: A chromosome-level genome assembly for the eastern fence lizard (Sceloporus undulatus), a reptile model for physiological and evolutionary ecology
Source: Gigascience. 2021 Oct 1;10(10):giab066. doi: 10.1093/gigascience/giab066 (PMC8486681; doi:10.1093/gigascience/giab066)
Supplement: giab066_Response_to_Reviewer_Comments_Original_Submission [file giab066_response_to_reviewer_comments_original_submission.pdf]

Author's Response To Reviewer Comments

Close

Dear Editors and Reviewers,

Thank you for the review of our manuscript. We found the reviews insightful and responding to the comments have allowed us to improve the manuscript considerably. Below we have addressed every comment in Blue, and added or edited text in Blue italics, and when we have made changes in the manuscript we indicate the page and line numbers as well as use tracked changes in the manuscript. We think you will find these revisions have improved the manuscript.

Thank you,
Tonia

Reviewer #1: The manuscript "A chromosome-level genome assembly for the Eastern fence lizard (Sceloporus undulatus), a reptile model for physiological and evolutionary ecology" by Westfall et al, reports a genome assembly that is likely to be of use to labs working in this and other reptilian systems. There are a few aspects of the presentation that are a bit confusing or are otherwise in need of revision, but I suspect these can be resolved. Specific comments are outlined below.

1) It in the context of the way the manuscript is laid out it would have been interesting to see the results of a PacBio only assembly, and/or an assembly that intergrated PacBio at an earlier stage, similar to the VGP assembly pipeline (https://vertebrategenomesproject.org/phase-one). Given the timescale of resource development and use of DoveTail (see also comment 2) I can understand why the reported approach was used, though it may not have been optimal.

Response: Thank you for this comment. We agree that comparing different combinations of the order in which we used the data types would be interesting. Unfortunately, because of how our current data was collected these would not be valid comparisons, particularly because the low level of coverage with the PacBio reads were collected specifically to complement the previous assembly and not sufficient for de novo assembly.

2) The use of HiRise (Dovetail) in the assembly pipeline raises some issues with reproducibility as that program is maintained as closed source code. As such it will be impossible for anyone to independently replicate the published assembly using the same methods reported in the paper. This may change in the future if the code is released, and I encourage the authors to request its release. If this request is not granted the authors should make sure to include the software version used for this assembly and all relevant assembly/filtration parameters, as well as a .agp (or similar) file that relays mapping evidence and weights that were used in the scaffolding process.

Response: As correctly indicated by the reviewer the HiRise Pipeline is proprietary to DoveTail Genomics, who did the sequencing and assembly for that portion of this project. Unfortunately, upon contacting DoveTail Genomics they notified us that the production files older than 3 years are purged from their system so the specific run files are not available. Therefore, we are not able to provide those requested files. We have included the HiRise version number and parameters in the main text (see below) for the readers to evaluate.

Page 5, line 127, text added:
"The data from both Hi-C and the 10X Genomics were used for assembly in the HiRise software (v2.1.3-5ce4af34ac25) pipeline at DoveTail Genomics. The pipeline excludes contigs/scaffolds < 1kb and only uses MQ>50 reads for scaffolding. The reads were aligned with a modified SNAP pipeline."

3) It is unclear to me if the assignment of the sex chromosome is valid. It appeals that the only evidence used to support this is homology to the anole sex chromosome. In a lineage with variable sex

chromosomes it would seem that this would provide scant evidence in favor of a particular chromosomes being the sex chromosome. Is there any other information that can be leveraged here? If not I would recommend dropping this section and the paragraph immediately preceeding the discussion.

Response: Thank you for this comment, we now see how this section can be clarified, and we have added text to clarify this point (see below). We have included additional information in this section for why we think this approach would likely identify the sex chromosome. But, we have also added "putative" in multiple places including Figure 5 to emphasize that this finding needs to be empirically confirmed. The paragraph above the discussion now describes the results from an additional, separate synteny analysis that further supports our original inference that the fourth predicted microchromosome is the putative X chromosome.
Page 9, line 266, the following text has been added to the paragraph describing the annotation of the putative X chromosome:
"These heteromorphisms are likely the result of other chromosomes' fusions to the X, as Sceloporus are among the large portion of iguanian lizards with conserved sex chromosomes, and another Sceloporus species within the same broad 2n=22 radiation, Sceloporus malachiticus, has an X chromosome homologous to the green anole X, but fused to several microchromosomes [68]. Given the observed homology, we used known X chromosome genes from the green anole to identify the scaffold likely representing the X chromosome within S. undulatus independently from other synteny analysis."
Page 9, line 279:
"This result, that the fourth predicted microchromosome is the putative X chromosome, is further supported by a separate synteny analysis described below"
Page 14, line 457, we have edited this text in the last paragraph above the discussion to read:
"These synteny results further support that the fourth largest microchromosome in the SceUnd1.0 assembly is syntenic to the anole X chromosome (Figure 3, Figure 5). However, it is not syntenic to the python X chromosome, which is syntenic to the Z chromosome in other snakes. The tegu sex chromosome has not been identified. Based on the blast hits from the anole X-linked genes and this synteny analysis we define the fourth largest microchromosome in the SceUnd1.0 assembly as the putative X chromosome, but functional data are needed to confirm this assignment."

We have also edited the paragraph at the top of page 14 that further discusses the fusion and fission of chromosomes in squamates.

4) The presentation of data from the 34 additional species could also use some shoring up, particularly given the fact that these mostly rely on reduced representation data that should subsample ~2% of the genome (notably, the guess of 2% may be wrong here, can the authors provide an estimate). How much overlap is there in the physical regions sampled in each of the 34 species? Is it nearly the same 2% or are they sampling wildly different intervals? This is likely to be of interest to several groups that are using reduced representation sequencing for comparative studies. The new genome assembly uniquely positions the authors to address this and they seem to already have the necessary data in hand.

Response: We have included additional analyses for the reader to better understand the overlap among the 34 Sceloporus reference-based assemblies. We used bedtools to count (1) the number of bases that have coverage by a specific number of reference-based genome assemblies (1 to 34 of the assemblies), and (2) the accumulating fraction of the genome that is covered by 1 to 34 reference-based assemblies. We provide these as bar graphs in Supplemental Figure 4 (Figure S4). We also provide an example of how these data can be used by focusing on one gene of interest to our group, IGF1. Using the coordinates in the annotation in the .gff3 from the SceUnd1.0 we pulled out the exons for the target gene, IGF1. These were aligned and visualized in Geneious. We found 16 of the 34 species had >75==% coverage across the protein coding region of this gene, 24 of them had >50% coverage. This is sufficient coverage to calculate estimates of genetic variation, selection analysis, and gene tree comparisons.

Page 13, Line 421 we edited the text to read:
"However, together these draft genomes contain a substantial amount of data that can be used for comparative genomic analyses. Figure S4 demonstrates the overlap in coverage of SceUnd1.0 by the reference-based genome assemblies. These distributions estimate that 50% of the genome would be covered by a subset of 16 species. Focusing on one individual gene of interest to our group, IGF1, we found that 16 of the 34 species had >75% coverage across the protein coding region of this gene and 24 of 34 had >50% coverage (Figure S4). Therefore, this dataset should prove useful for analyses of

protein and gene sequence evolution to understand behavioral ecology, physiology, developmental biology, and more."

5) Related to this, for cases where substantially more than 2% of the genome is corrected. How is this distributed? In repetitive elements? Single-copy sequence? And why does this deviate from the expected ~2% in the cases that is does (e.g. 44% in one case).

Response: The 2% estimate is based on in silico experiments. It seems there was considerable by-catch in much of the reduced-representation sequencing, which is normally filtered out when those reduced representation data are being analyzed. With this by-catch, we were able to recover a reasonable amount (20-40%) of the genome sequence at low (1-3X) coverage for most species. We plotted the distribution of coverage across the genomes for each species to demonstrate the low-level coverage (Figure S3).

Page 12, Line 402: We have edited to the text in this section to the following
"It seems there was a considerable amount of by-catch in much of the reduced-representation sequencing that is normally filtered out when those reduced representation data are being analyzed. For the species with ~5Gb of sequencing data, we improved the genome coverage from an average of 1.23% to an average of 44.4% coverage at low depth (1-3X) (Figure S3). For S. occidentalis with ~ 41Gb of data, coverage improved from 61.0% to 88.7% (Table 7) at an average depth of ~ 20X (Figure S3)."

6) The authors cite reference 17 in support of the need to sample additional squamates. This reference focuses on repetitive elements, and is therefore notable that the authors do not report and data on the distribution or density of repeats. An analysis of repeat content/classification was likely done as part of the annotation pipelines and I think a brief summary would be a welcome addition to the report.

Response: Thank you for this suggestion. We have conducted a repeat analysis and have included it as an additional text section in the manuscript starting on Page 9, as well as a figure (Figure 3) and a supplementary table (Table S4) that describe the distribution of repeat elements in S. undulatus with an evolutionary contrast to A. carolinensis.

Minor comments:
Line 102 - the species name is misspelled
Response: Thank you for catching this typo, it is now corrected.

Line 381 - species name is not italicized
Response: Thank you for catching this typo, it is now corrected.

Reviewer #2: The authors represented us a genome assembly of Sceloporus undulates, which utilized three sequencing technologies including 10X linked reads, Hi-C data, and long reads from the PacBio sequencing platform. I totally agree with the authors to one of their main conclusions that the SuperNova assembly was sufficient for mapping RNAseq and whole-genome resequencing. Although such a conclusion could have long been recognized, it is helpful that the authors can prove it using this new dataset. In addition, a high-quality genome of S. undulates is a good addition to what we have for the squamate genomic data. Overall the manuscript is well developed. However, there are a couple of points that the authors should acknowledge and discuss:

Major:

(1) Please provide several important assessments to validate the genome assembly accuracy. For instance, genome size should be estimated to check whether the assembly can represent the entire genomic info or contains redundancies; A GC-depth plot should be included to demonstrate whether the assembly contains contaminations from other species; Contig N50 (different form scaffold N50) should be added to demonstrate the assembly contiguity level.

Response: Thank you for these suggestions. We have now provided citations for genome size estimates for related species for comparison, as well as a GC-depth plot to test for contamination from other species, which appears negligible. We added Contig N50 to Table 1.

Page 5-6, starting on line 153, we have added the following text:
"Estimated genome size of the closely related species Sceloporus occidentalis is 2.36GB based on fulcan densiometry. Assuming S. undulatus is similar, the 1.9GB of sequence in our SceUnd1.0 assembly is likely either missing some data, or repeat regions have been condensed, creating redundancies. To assess the level of contamination in our SceUnd1.0 genome assembly we used Blobtools (v1) (Laetsch DR and Blaxter ML, 2017) workflow A that estimated contamination based on GC content difference that exist between taxa. In order to visualize depth by GC content for taxa represented in the assembly, we created a blobDB using a BAM file to infer coverage, sequence similarity hits based on the DIAMOND blast, and the SceUnd1.0 assembly fasta file. Plots were produced for two taxonomic ranks, phylum and order, with taxonomic annotation based on the "bestsum" taxrule. The majority of the represented taxa in the assembly were annotated as belonging to Chordata (phylum level) and Squamata (order level). There is a smaller, but visible, proportion of reads that are associated with order Testudines, which is likely due to regions of sequence similarity across reptiles. Overall, the plot demonstrates negligible contamination of other taxa (Figure S1)."

(2) The authors claimed that they conducted reference-based assemblies for 34 Sceloporus species to improve draft nuclear genomes assemblies from 1% coverage to 43% coverage. This information is rather misleading. Firstly, De novo assembly can hardly represent a widely accepted method when dealing with the reduced presentation data (also known as RAD-Seq or GBS data). Therefore, such a comparison doesn't mean much. Secondly, the authors should tone down this part, as readers would expect to make use of them directly when the authors mentioned them as draft genome assemblies. However, I can hardly find a scenario in which those assemblies can be reused directly. I would choose the S. undulates genome as a reference, instead of any those "draft genome assemblies", to conduct, for example, resequencing-based population studies.

Response: The goal of the reference-based assemblies was not intended to be a comparison to the de novo assemblies to determine which is a better method, but rather to demonstrate improvement of the assemblies as a resource for the community. These reference-based assemblies are not necessarily intended to be used as References in of themselves. The reviewer is correct that SceUnd1.0 would be the best assembly to map to (we have removed that phrase of the sentence). Nonetheless, these draft genomes contain a significant amount of data that can be used for comparative genomic analyses. We have provided additional analyses to demonstrate the number of genomes that can be sampled for a particular fraction of the genome, see Figure S4. These analyses demonstrate that, for ~50% of the bases in the SceUnd1.0 assembly, 16 species would be represented. We also provide an example, focusing on one gene of interest to our group, IGF1, we found that 16 of the 34 species had >75% coverage across the protein coding region of this gene, and 24 of them had >50% coverage. This is sufficient coverage to calculate estimates of genetic variation, selection analysis, and gene tree comparisons.

Page 13, Line 421 we edited the text to read:
"However, these draft genomes contain a substantial amount of data that can be used for comparative genomic analyses. Figure S4 demonstrates the overlap in coverage of SceUnd1.0 by the reference-based genome assemblies. These distributions estimate that 50% of the genome would be covered by a subset of 16 species. Just focusing on one gene of interest to our group, IGF1, we found that 16 of the 34 species had >75% coverage across the protein coding region of this gene, and 24 of them had >50% coverage (Figure S4). Thereby, this dataset should prove useful for analyses of protein and gene sequence evolution to understand behavioral ecology, physiology, developmental biology, and more."

(3) the authors claimed that the addition of PB long reads data provided negligible gains. PBJelly mainly works for gap-closing, so there might be a significant improvement in Contig N50 value, which can only be confirmed after the authors provide such data. In addition, the authors could use some other software, such as this one (http://biorxiv.org/content/10.1101/831248v1.full), which claimed to perform ten times better than PBJelly.

Response: We have added the Contig N50 to Table 1. Thank you for the suggestion to use the program presented in the BioRXiv link. We do not intend to reassemble and redo the analyses for this manuscript as other manuscripts are already in preparation or have been submitted that have used the current SceUnd1.0 Assembly. But we will definitely consider using the reference program in future versions as we continue to improve further versions of this genome reference.

Minor:

(4) Line 131: it is better to provide the genome coverage information (for example, 40X) in the main text as well.

Response: Thank you for this suggestion, it has been incorporated.

(5) line 163: scaffolds as long as 7 MB might not be mentioned as tiny scaffolds.

Response: Thank you for this suggestion. We have changed "tiny" to "smaller".

(6) Line 179 - 181: the authors may obtain useful information from this paper, 10.1093/gigascience/giy163, which demonstrates the potential problems regarding the 10X genome assembly - generate lots of gaps and produce redundancies.

Response: Thank you for bringing this reference to our attention. While it is an interesting paper, we are uncertain what action (if any) is being requested by this comment in the context of revising the manuscript.

(7) line 259: the authors may want to give some clues for how to improve the genome annotation. Plus, the authors may provide some details on why those missing BUSCO genes failed to be annotated, was it attributed to sequence errors which cause nonsense mutations? If so, genome polishing may correct them.

Response: The SceUnd1.0 has been submitted to NCBI and is in line for their annotation pipeline and then it will be moved into ENSEMBL. We suspect this pipeline would improve the annotation. As this genome assembly is already being used for multiple projects, with manuscript preparation, undoubtedly efforts will continue to improve upon this assembly. In terms of the missing BUSCOS, the BUSCOs were for Tetrapoda and it is possible some of the genes are truly missing in S. undulatus. Because there were more BUSCOs fragmented or missing from the predicted proteins (the annotation) than the actual genomic sequence itself, we attribute those to annotation errors, not errors in the assembly.

Page 8, line 255 we have edited this text to read:
"Because there were more BUSCOs fragmented or missing from the predicted proteins (the annotation) than the actual genomic sequence itself, we attribute those to annotation errors, not errors in the assembly, which suggests this first version of annotation can be improved."

(8) Line 278: For the mitogenome assembly, there is a big chance that de novo RNAseq assembly can obtain whole or part of the mitochondrial genome. It won't be hard work, and it is interesting to examine whether reference-based mito-genome assembly can introduce some unexpected bias.

Response: We used the program MitoZ to de-novo assemble the mitochondrial genome using RNA-seq paired-end sequencing data from 18 individuals (of the same population). We used several techniques to go about performing this assembly:

1) Assembly for each individual using the Quick Assembly approach without a taxonomic constraint
2) Assembly for each individual using the Multi-Kmer approach without a taxonomic constraint
3) Assembly for each individual using the Quick Assembly approach with a taxonomic constraint (Phrynosomatidae)
4) Assembly for each individual using the Multi-Kmer approach with a taxonomic constraint (Phrynosomatidae)
5) Assembly of all combined reads using the Quick Assembly approach
6) Assembly of all combined reads using the Multi-Kmer approach
7) Assembly of only those reads that mapped to the Sceloporus occidentalis mitochondrial genome using the Quick Assembly approach
8) Assembly of only those reads that mapped to the Sceloporus occidentalis mitochondrial genome using the Multi-Kmer approach

None of these approaches recovered the full mitochondrial genome (each resulted in missing protein coding genes, tRNA, and rRNA). Further, different approaches sometimes selected different scaffolds as those pertaining to the mitochondrial genome. These inconsistencies and lack of data led us to the

decision to retain our approach described in the original manuscript submission, wherein we mapped the raw reads to the Sceloporus undulatus mitochondrial genome, aligned this genome with Anolis carolinensis, and transferred annotations.

Page 10, line 327, we add the following sentence to the manuscript.
"While this genome is useful for understanding sequence variation and comparative genomics and phylogenetic analyses, this mitochondrial genome should not be used for examination of mitochondrial genome structure."

As for extracting the genomes from the other 34 Sceloporus species for examination of species relationships, this is not within the scope of this study and is an objective being pursued by other labs using more appropriate datasets.

(9) Line 318: can the taxonomic uncertainty be resolved using the resequencing data, e.g. you can obtain their mito-genomes via software like Novoplasty, MitoZ, et al.

Response: This question of taxonomic uncertainty of the populations in this region is currently being addressed in more depth by other research labs as is outside of the scope of this manuscript.

(10) Line 328: Can FLAGSTAT work on sam files? Or do you mean bam files?

Response: Yes FLAGSTAT can work on sam files.

(11) Line 345 - 347: How to evaluate the correctness of those increased SNPs?

Response: We are not exactly sure what this question is asking. We did not actually call SNPs with the WGS database, but used the theoretical HET SNP sensitivity metric to estimate the utility of each assembly for calling SNPs. The full WGS dataset is part of a much bigger study across S. undulatus populations and will be published at a later date. We try to further clarify the theoretical HET SNP sensitivity metric and how we are using it as a metric for useability of the assemblies in the main text. We have added the following text.

Page 11, Line 362:
"The CollectWgsMetrics tool from the Picard Toolkit [85] was used to calculate genome-wide coverage of the mapped reads for each individual and assembly, and theoretical HET SNP sensitivity (a metric based on coverage and base quality distribution that estimates probability of calling a true heterozygote SNP) as a way to predict the utility of each assembly as a reference for calling SNPs at high a low coverage. "

Line 375: "This may be due to repetitive regions being added to the assembly by the PacBio data making it slightly less mappable. "

(12) Line 370: 41Gb -> 40.8Gb or ~ 41 Gb
Response: Thank you for catching this typo, it is now corrected.

(13) Line 397: please clarify the criteria and methods that produce those markers of 1,000 bp in length.
Response: We have clarified this method with the following statement:

Line 436" "The SceUnd1.0 scaffolds representing the 11 putative chromosomes were each divided into 1000 bp-long sequences that excluded gapped regions to serve as markers."

(14) Line 400: > 80% identity?
Response: Thank you for catching this typo, it is now corrected.

(15) Line 443: PBJelly cannot aid in chromosome-level assembly.

Response: Good point. We have now rephrased this to be:

Now Line 487
"Our study demonstrates that the SuperNova Assembly was sufficient for mapping RNAseq and whole genome resequencing, while the more expensive data from HiC and PacBio were necessary to achieve high-level continuity and chromosome-level scaffolding in the HiRise and PBJ Assemblies."

Close
