# Supplementary material for: A chromosome-level genome assembly for the eastern fence lizard (Sceloporus undulatus), a reptile model for physiological and evolutionary ecology
Source: Gigascience. 2021 Oct 1;10(10):giab066. doi: 10.1093/gigascience/giab066 (PMC8486681; doi:10.1093/gigascience/giab066)
Supplement: giab066_Response_to_Reviewer_Comments_Revision_1 [file giab066_response_to_reviewer_comments_revision_1.pdf]

Author's Response To Reviewer Comments

Close

Dear Editors and Reviewers,

Thank you for the re-review of our manuscript. We found the reviews insightful and responding to the comments have allowed us to improve the manuscript considerably. Below we have addressed every comment in Blue, and added or edited text in Blue italics, and when we have made changes in the manuscript we indicate the line numbers and use tracked changes in the manuscript. We think you will find these revisions have improved the manuscript.

Thank you,
Tonia

Reviewer #1: The authors seem to have appropriately addressed previous concerns where possible and I think there are only three concerns that should be addressed prior to publication.

1) The assembly methods are not reproducible. Because Dovetail uses a proprietary assembly algorithm that is closed access and closed source, and moreover failed to retain information necessary to evaluate the assembly, it would be nearly impossible for others (or the authors themselves) to replicate the assembly. This is not the authors' fault, but it is hard to ignore in a world where rigor and reproducibility are paramount. I am happy to defer to the editors with respect to how this can be resolved, but I feel it should not simply be ignored.

Response: Thank you for this comment. We agree in principle to this comment, and we have done our best to provide what information we can on the assembly parameters provided by Dovetail. On line 137 we cite the papers that describe the HiRise program and the GITHUB for the HiRise scaffolder source code. In the text we provide the extent of the information that we were able to get from multiple conversations with the staff at DoveTail Genomics; on lines 129-130 we describe the parameter setting that were used for the HiRise assembly, and on lines 125-137 we list the parameters settings for the PBJelly assembly.

Minor Comments
1) Ling 154 "fulcan densitometry" - This should be "Feulgen densitometry" with appropriate capitalization.
Response: Thank you for bringing this typo to our attention. This is now corrected.

2) Figure 5 - coloration of Anolis sex and microchromosomes is obscured by the bounding boxes. This should be fixed and the "ma" "mi" labels formally defined in the legend.
Response: Thank you for bringing this to our attention. We have replaced that figure with one that has the Anolis microchromosomes are expanded, and we have removed the "ma" and "mi" labels. We have labeled the chromosomes continuously and have removed any corresponding labels in the text on page 14.

Reviewer #2: Authors replied satisfactorily to most of the comments. I have only two notes on the wording in the abstract section.

(1) "improved these assemblies from 1% coverage to 43% coverage on average for 34 additional Sceloporus species" will definitely mislead readers. Please rephrase it to avoid confusion, or remove it from main findings;

Response: Thank you for this comment. We have edited the abstract to remove that phrase.
Lines 39-40: We also used this new assembly to provide improved reference-based genome assemblies for 34 additional Sceloporus species.

(2) PB long reads did improve contig N50 > 2 times, which is not negligible gain.

Response: Thank you for this comment, we clarify this statement to read:
Lines 48-49: The subsequent addition of PacBio data doubled the contig N50, but provided negligible gains in scaffold length.

Close
