# Supplementary material for: A chromosome-level genome assembly for the eastern fence lizard (Sceloporus undulatus), a reptile model for physiological and evolutionary ecology
Source: Gigascience. 2021 Oct 1;10(10):giab066. doi: 10.1093/gigascience/giab066 (PMC8486681; doi:10.1093/gigascience/giab066)
Supplement: giab066_Reviewer_1_Report_Original_Submission — Jeramiah J Smith -- 7/31/2020 Reviewed [file giab066_reviewer_1_report_original_submission.pdf]

Reviewer Report

Title: A chromosome-level genome assembly for the Eastern fence lizard (Sceloporus undulatus), a reptile model for physiological and evolutionary ecology

Version: Original Submission **Date:** 7/31/2020

Reviewer name: Jeramiah J Smith

Reviewer Comments to Author:

The manuscript "A chromosome-level genome assembly for the Eastern fence lizard (Sceloporus undulatus), a reptile model for physiological and evolutionary ecology" by Westfall et al, reports a genome assembly that is likely to be of use to labs working in this and other reptilian systems. There are a few aspects of the presentation that are a bit confusing or are otherwise in need of revision, but I suspect these can be resolved. Specific comments are outlined below.

1) It in the context of the way the manuscript is laid out it would have been interesting to see the results of a PacBio only assembly, and/or an assembly that intergrated PacBio at an earlier stage, similar to the VGP assembly pipeline (https://vertebrategenomesproject.org/phase-one). Given the timescale of resource development and use of DoveTail (see also comment 2) I can understand why the reported approach was used, though it may not have been optimal.

2) The use of HiRise (Dovetail) in the assembly pipeline raises some issues with reproducibility as that program is maintained as closed source code. As such it will be impossible for anyone to independently replicate the published assembly using the same methods reported in the paper. This may change in the future if the code is released, and I encourage the authors to request its release. If this request is not granted the authors should make sure to include the software version used for this assembly and all relevant assembly/filtration parameters, as well as a .agp (or similar) file that relays mapping evidence and weights that were used in the scaffolding process.

3) It is unclear to me if the assignment of the sex chromosome is valid. It appeals that the only evidence used to support this is homology to the anole sex chromosome. In a lineage with variable sex chromosomes it would seem that this would provide scant evidence in favor of a particular chromosomes being the sex chromosome. Is there any other information that can be leveraged here? If not I would recommend dropping this section and the paragraph immediately preceeding the discussion.

4) The presentation of data from the 34 additional species could also use some shoring up, particularly given the fact that these mostly rely on reduced representation data that should subsample ~2% of the genome (notably, the guess of 2% may be wrong here, can the authors provide an estimate). How much overlap is there in the physical regions sampled in each of the 34 species? Is it nearly the same 2% or are they sampling wildly different intervals? This is likely to be of interest to several groups that are using reduced representation sequencing for comparative studies. The new genome assembly uniquely positions the authors to address this and they seem to already have the necessary data in hand.

5) Related to this, for cases where substantially more than 2% of the genome is corrected. How is this distributed? In repetitive elements? Single-copy sequence? And why does this deviate from the

expected ~2% in the cases that is does (e.g. 44% in one case).
6) The authors cite reference 17 in support of the need to sample additional squamates. This reference focuses on repetitive elements, and is therefore notable that the authors do not report and data on the distribution or density of repeats. An analysis of repeat content/classification was likely done as part of the annotation pipelines and I think a brief summary would be a welcome addition to the report.
Minor comments:
Line 102 - the species name is misspelled
Line 381 - species name is not italicized
Sincerely,
Jeramiah Smith

Level of Interest

Please indicate how interesting you found the manuscript: Choose an item.

Quality of Written English

Please indicate the quality of language in the manuscript: Choose an item.

Declaration of Competing Interests

Please complete a declaration of competing interests, considering the following questions:

- Have you in the past five years received reimbursements, fees, funding, or salary from an organisation that may in any way gain or lose financially from the publication of this manuscript, either now or in the future?
- Do you hold any stocks or shares in an organisation that may in any way gain or lose financially from the publication of this manuscript, either now or in the future?
- Do you hold or are you currently applying for any patents relating to the content of the manuscript?
- Have you received reimbursements, fees, funding, or salary from an organization that holds or has applied for patents relating to the content of the manuscript?
- Do you have any other financial competing interests?
- Do you have any non-financial competing interests in relation to this paper?

If you can answer no to all of the above, write 'I declare that I have no competing interests' below. If your reply is yes to any, please give details below.

I interviewed for a position at Penn State in 2018 and was reimbursed for costs related to that interview. Some of the authors are from that institution.

I agree to the open peer review policy of the journal. I understand that my name will be included on my report to the authors and, if the manuscript is accepted for publication, my named report including any attachments I upload will be posted on the website along with the authors' responses. I agree for my report to be made available under an Open Access Creative Commons CC-BY license (http://creativecommons.org/licenses/by/4.0/). I understand that any comments which I do not wish to be included in my named report can be included as confidential comments to the editors, which will not be published.

Choose an item.

To further support our reviewers, we have joined with Publons, where you can gain additional credit to further highlight your hard work (see: https://publons.com/journal/530/gigascience). On publication of this paper, your review will be automatically added to Publons, you can then choose whether or not to claim your Publons credit. I understand this statement.

Yes Choose an item.
