# Supplementary material for: A chromosome-level genome assembly for the eastern fence lizard (Sceloporus undulatus), a reptile model for physiological and evolutionary ecology
Source: Gigascience. 2021 Oct 1;10(10):giab066. doi: 10.1093/gigascience/giab066 (PMC8486681; doi:10.1093/gigascience/giab066)
Supplement: giab066_Reviewer_1_Report_Revision_1 — Jeramiah J Smith -- 5/25/2021 Reviewed [file giab066_reviewer_1_report_revision_1.pdf]

Reviewer Report

Title: A chromosome-level genome assembly for the Eastern fence lizard (Sceloporus undulatus), a reptile model for physiological and evolutionary ecology

Version: Revision 1 **Date:** 5/25/2021

Reviewer name: Jeramiah J Smith

Reviewer Comments to Author:

The authors seem to have appropriately addressed previous concerns where possible and I think there are only three concerns that should be addressed prior to publication.
1) The assembly methods are not reproducible. Because Dovetail uses a proprietary assembly algorithm that is closed access and closed source, and moreover failed to retain information necessary to evaluate the assembly, it would be nearly impossible for others (or the authors themselves) to replicate the assembly. This is not the authors' fault, but it is hard to ignore in a world where rigor and reproducibility are paramount. I am happy to defer to the editors with respect to how this can be resolved, but I feel it should not simply be ignored.
Minor Comments
1) Ling 154 "fulcan densitometry" - This should be "Feulgen densitometry" with appropriate capitalization.
2) Figure 5 - coloration of Anolis sex and microchromosomes is obscured by the bounding boxes. This should be fixed and the "ma" "mi" labels formally defined in the legend.

Level of Interest

Please indicate how interesting you found the manuscript: Choose an item.

Quality of Written English

Please indicate the quality of language in the manuscript: Choose an item.

Declaration of Competing Interests

Please complete a declaration of competing interests, considering the following questions:

- Have you in the past five years received reimbursements, fees, funding, or salary from an organisation that may in any way gain or lose financially from the publication of this manuscript, either now or in the future?
- Do you hold any stocks or shares in an organisation that may in any way gain or lose financially from the publication of this manuscript, either now or in the future?

- Do you hold or are you currently applying for any patents relating to the content of the manuscript?
- Have you received reimbursements, fees, funding, or salary from an organization that holds or has applied for patents relating to the content of the manuscript?
- Do you have any other financial competing interests?
- Do you have any non-financial competing interests in relation to this paper?

If you can answer no to all of the above, write 'I declare that I have no competing interests' below. If your reply is yes to any, please give details below.

I declare that I have no competing interests

I agree to the open peer review policy of the journal. I understand that my name will be included on my report to the authors and, if the manuscript is accepted for publication, my named report including any attachments I upload will be posted on the website along with the authors' responses. I agree for my report to be made available under an Open Access Creative Commons CC-BY license (http://creativecommons.org/licenses/by/4.0/). I understand that any comments which I do not wish to be included in my named report can be included as confidential comments to the editors, which will not be published.

Choose an item.

To further support our reviewers, we have joined with Publons, where you can gain additional credit to further highlight your hard work (see: https://publons.com/journal/530/gigascience). On publication of this paper, your review will be automatically added to Publons, you can then choose whether or not to claim your Publons credit. I understand this statement.

Yes Choose an item.
