# Supplementary material for: A chromosome-level genome assembly for the eastern fence lizard (Sceloporus undulatus), a reptile model for physiological and evolutionary ecology
Source: Gigascience. 2021 Oct 1;10(10):giab066. doi: 10.1093/gigascience/giab066 (PMC8486681; doi:10.1093/gigascience/giab066)
Supplement: giab066_Reviewer_2_Report_Original_Submission — Shanlin Liu -- 8/4/2020 Reviewed [file giab066_reviewer_2_report_original_submission.pdf]

Reviewer Report

Title: A chromosome-level genome assembly for the Eastern fence lizard (Sceloporus undulatus), a reptile model for physiological and evolutionary ecology

Version: Original Submission **Date:** 8/4/2020

Reviewer name: Shanlin Liu

Reviewer Comments to Author:

The authors represented us a genome assembly of Sceloporus undulates, which utilized three sequencing technologies including 10X linked reads, Hi-C data, and long reads from the PacBio sequencing platform. I totally agree with the authors to one of their main conclusions that the SuperNova assembly was sufficient for mapping RNAseq and whole-genome resequencing. Although such a conclusion could have long been recognized, it is helpful that the authors can prove it using this new dataset. In addition, a high-quality genome of S. undulates is a good addition to what we have for the squamate genomic data. Overall the manuscript is well developed. However, there are a couple of points that the authors should acknowledge and discuss:

Major:

(1) Please provide several important assessments to validate the genome assembly accuracy. For instance, genome size should be estimated to check whether the assembly can represent the entire genomic info or contains redundancies; A GC-depth plot should be included to demonstrate whether the assembly contains contaminations from other species; Contig N50 (different form scaffold N50) should be added to demonstrate the assembly contiguity level.

(2) The authors claimed that they conducted reference-based assemblies for 34 Sceloporus species to improve draft nuclear genomes assemblies from 1% coverage to 43% coverage. This information is rather misleading. Firstly, De novo assembly can hardly represent a widely accepted method when dealing with the reduced presentation data (also known as RAD-Seq or GBS data). Therefore, such a comparison doesn't mean much. Secondly, the authors should tone down this part, as readers would expect to make use of them directly when the authors mentioned them as draft genome assemblies. However, I can hardly find a scenario in which those assemblies can be reused directly. I would choose the S. undulates genome as a reference, instead of any those "draft genome assemblies", to conduct, for example, resequencing-based population studies.

(3) the authors claimed that the addition of PB long reads data provided negligible gains. PBJelly mainly works for gap-closing, so there might be a significant improvement in Contig N50 value, which can only be confirmed after the authors provide such data. In addition, the authors could use some other software, such as this one (http://biorxiv.org/content/10.1101/831248v1.full), which claimed to perform ten times better than PBJelly.

Minor:

(4) Line 131: it is better to provide the genome coverage information (for example, 40X) in the main text as well.

(5) line 163: scaffolds as long as 7 MB might not be mentioned as tiny scaffolds.

(6) Line 179 - 181: the authors may obtain useful information from this paper, 10.1093/gigascience/giy163, which demonstrates the potential problems regarding the 10X genome assembly - generate lots of gaps and produce redundancies.
(7) line 259: the authors may want to give some clues for how to improve the genome annotation. Plus, the authors may provide some details on why those missing BUSCO genes failed to be annotated, was it attributed to sequence errors which cause nonsense mutations? If so, genome polishing may correct them.
(8) Line 278: For the mitogenome assembly, there is a big chance that de novo RNAseq assembly can obtain whole or part of the mitochondrial genome. It won't be hard work, and it is interesting to examine whether reference-based mito-genome assembly can introduce some unexpected bias.
(9) Line 318: can the taxonomic uncertainty be resolved using the resequencing data, e.g. you can obtain their mito-genomes via software like Novoplasty, MitoZ, et al.
(10) Line 328: Can FLAGSTAT work on sam files? Or do you mean bam files?
(11) Line 345 - 347: How to evaluate the correctness of those increased SNPs?
(12) Line 370: 41Gb -> 40.8Gb or ~ 41 Gb
(13) Line 397: please clarify the criteria and methods that produce those markers of 1,000 bp in length.
(14) Line 400: > 80% identity?
(15) Line 443: PBJelly cannot aid in chromosome-level assembly.

Level of Interest

Please indicate how interesting you found the manuscript: Choose an item.

Quality of Written English

Please indicate the quality of language in the manuscript: Choose an item.

Declaration of Competing Interests

Please complete a declaration of competing interests, considering the following questions:

- Have you in the past five years received reimbursements, fees, funding, or salary from an organisation that may in any way gain or lose financially from the publication of this manuscript, either now or in the future?
- Do you hold any stocks or shares in an organisation that may in any way gain or lose financially from the publication of this manuscript, either now or in the future?
- Do you hold or are you currently applying for any patents relating to the content of the manuscript?
- Have you received reimbursements, fees, funding, or salary from an organization that holds or has applied for patents relating to the content of the manuscript?
- Do you have any other financial competing interests?

- Do you have any non-financial competing interests in relation to this paper?

If you can answer no to all of the above, write 'I declare that I have no competing interests' below. If your reply is yes to any, please give details below.

I declare that I have no competing interests

I agree to the open peer review policy of the journal. I understand that my name will be included on my report to the authors and, if the manuscript is accepted for publication, my named report including any attachments I upload will be posted on the website along with the authors' responses. I agree for my report to be made available under an Open Access Creative Commons CC-BY license (http://creativecommons.org/licenses/by/4.0/). I understand that any comments which I do not wish to be included in my named report can be included as confidential comments to the editors, which will not be published.

Choose an item.

To further support our reviewers, we have joined with Publons, where you can gain additional credit to further highlight your hard work (see: https://publons.com/journal/530/gigascience). On publication of this paper, your review will be automatically added to Publons, you can then choose whether or not to claim your Publons credit. I understand this statement.

Yes Choose an item.
